# Artesunate Combined With Metformin Ameliorate on Diabetes-Induced Xerostomia by Mitigating Superior Salivatory Nucleus and Salivary Glands Injury in Type 2 Diabetic Rats via the PI3K/AKT Pathway

**DOI:** 10.3389/fphar.2021.774674

**Published:** 2021-12-20

**Authors:** Siqin Zhang, Jiarui Li, Xiaolin Nong, Yuxiang Zhan, Jiazhi Xu, Danni Zhao, Chubin Ma, Yuchen Wang, Yixing Li, Zhan Li, Jiaquan Li

**Affiliations:** ^1^ College of Stomatology, Guangxi Medical University, Nanning, China; ^2^ Guangxi Key Laboratory of Oral and Maxillofacial Surgery Disease Treatment, Nanning, China; ^3^ Medical Science Research Center, Guangxi Medical University, Nanning, China; ^4^ Life Science Institute, Guangxi Medical University, Nanning, China

**Keywords:** artesunate, metformin, diabetic xerostomia, salivary gland, superior salivatory nucleus, PI3K/Akt pathway

## Abstract

Polydipsia and xerostomia are the most common complications that seriously affect oral health in patients with diabetes. However, to date, there is no effective treatment for diabetic xerostomia. Recent studies have reported that artesunate (ART) and metformin (Met) improve salivary gland (SG) hypofunction in murine Sjögren’s syndrome. Therefore, aim of this study was to investigate the effect and underlying mechanism of artesunate (ART) alone and in combination with metformin (Met) on hyposalivation in type 2 diabetes mellitus (T2DM) rats. T2DM rats were induced using a high-fat diet and streptozotocin. SPF male Sprague–Dawley rats were divided into the following five groups: normal control group, untreated diabetic group, ART-treated diabetic group (50 mg/kg), Met-treated diabetic group (150 mg/kg), and ART/Met co-treated diabetic group (50 mg/kg ART and 150 mg/kg Met). ART and Met were intragastrically administered daily for 4 weeks. The general conditions, diabetes parameters and serum lipids were evaluated after drug treatment. Furthermore, we observed changes in the central superior salivatory nucleus (SSN) and SG, and changes in the AQP5 expression, parasympathetic innervation (AChE and BDNF expression), and PI3K/AKT pathway- (p-AKT, and p-PI3K), apoptosis- (Bax, Bcl-2, and Caspase3), and autophagy- (LC3 and P62) related markers expression in T2DM rats after treatment. Our results showed that ART or Met alone and ART/Met combination attenuated a range of diabetic symptoms, including weight loss, urine volume increase, water consumption increase, hyperglycemia, insulin resistance, glucose intolerance and dyslipidemia. More importantly, we found that these three treatments, especially ART/Met combination, mitigated hyposalivation in the T2DM rats via improving the central SSN and SGs damage in hyperglycemia. Our data also indicated that ART/Met attenuated SG damage though regulating the PI3K/Akt pathway to inhibit apoptosis and autophagy of SGs in the T2DM rats. Moreover, ART/Met preserved parasympathetic innervation (AChE and BDNF expression) in SGs to alleviate diabetes-induced hyposalivation likely through rescuing central SSN damage. Taken together, these findings might provide a novel rationale and treatment strategy for future treatment of diabetes-induced xerostomia in the clinic.

## Introduction

Diabetes mellitus (DM) is a chronic multiple disease characterized by polyuria, polydipsia, polyphagia and weight loss and usually causes a systemic and multi-organ damage. Among them, diabetic xerostomia is one of the most common oral manifestations that seriously impacts the quality of life of diabetes patients (López-Pintor et al., 2016; Molania et al., 2017; Huang et al., 2018b); however, only few of the current studies have explored its causes. Studies have reported that diabetic patients with xerostomia are more susceptible to dental caries, oral candidiasis, taste disorders, gingivitis, fissured tongue, and other neurosensory disorders (Kudiyirickal and Pappachan, 2015; Mauri-Obradors et al., 2017), particularly in elderly people (Albert et al., 2012; Lima et al., 2017). In addition, accumulating evidence suggests that long-term oral infections secondary to diabetic xerostomia, such as periodontitis and dental caries, affect glycaemic control in diabetic patients, and also facilitate onset or progression of diabetes and its complications (Darré et al., 2008; Liccardo et al., 2019). However, the available treatment strategies for relieving or improving the symptoms of dry mouth are generally unsatisfactory and remain palliative. Therefore, this calls for development of new treatment strategies for diabetic patients with xerostomia.

Metformin (Met), a synthetic biguanide, is a first-line drug used in the clinic to treat and prevent type 2 diabetes mellitus (T2DM) and its various complications (Sanchez-Rangel and Inzucchi, 2017). Decades of clinical applications have proved that Met is safe and well-tolerated (Hostalek et al., 2015). Artesunate (ART) is a water-soluble derivative of artemisinin that is characterized by low toxicity, excellent tolerance, and multiple routes of administration such as oral and intravenous injection (Hien and White, 1993; Zuo et al., 2016). Furthermore, several ART pharmacological studies have demonstrated that it possesses a wide range of biological activities, with exception of anti-malarial effects, including anti-tumor, anti-microbes, anti-inflammation, antioxidant, and regulation of the immune system (Yu et al., 2016; Våtsveen et al., 2018; Zhang et al., 2018; Li et al., 2019). It has been demonstrated that Met mitigates hyposalivation and salivary gland (SG) inflammation in non-obese diabetic (NOD)/ShiLtj mice, an animal model of murine Sjögren’s syndrome, via activating 5′ adenosine monophosphate-activated protein kinase (Kim et al., 2019). In addition, one recent study has reported that ART ameliorates salivary secretion dysfunction in NOD/ShiLtj mice via regulating the TRAF6-mediated NF-κB signaling pathway (Zhan et al., 2021). Importantly, ART is not only able to pass through the blood-brain barrier and maintain a high concentration in the brain (Zhao and Song, 1989; Zuo et al., 2016), but is also able to adjust glucose homeostasis against diabetes and its complications (Sun et al., 2018; Xu et al., 2019; Maurya et al., 2021). Moreover, our previous study also showed that ART controlled blood glucose levels and prevented cardiovascular complications in diabetic rats (Chen et al., 2021). However, to date, no study has investigated effects of ART alone or in combination with Met against diabetic xerostomia and elucidated the underlying mechanisms.

Xerostomia, also known as dry mouth, is mainly characterized by reduced saliva secretion (Bhattarai et al., 2018). It is well known that salivary secretion is regulated by superior salivatory nucleus (SSN) located in the lateral reticular formation of the brainstem (Mitoh et al., 2017). Most of the currently available studies have primarily focused on pathophysiology of salivary gland injury caused by hyperglycemia (Huang et al., 2018b; Lee et al., 2020; Xiang et al., 2020), with few studies focusing on the pathological changes of SSN that regulates saliva secretion in hyperglycemia. Therefore, one of the primary objectives of this study was to fill the gap in the literature, which can aid in the development of therapeutic strategies to ameliorate diabetes-induced hyposalivation. We hypothesized that diabetic xerostomia may be associated with the SSN damage, SG injury, or both caused by hyperglycemia. Besides, the PI3K/AKT signaling pathway, which widely exists in eukaryotes, is associated with a variety of biological processes, including metabolism, cytoskeleton reorganization, macromolecular synthesis, cell proliferation, and differentiation (Keppler-Noreuil et al., 2016; Very et al., 2018). Recent studies have also demonstrated that deregulation of the PI3K/AKT pathway is a key feature of diabetes and its complications due to the pathway’s involvement in the regulation of insulin and glucose homeostasis (Huang et al., 2016; Maffei et al., 2018). Therefore, this study further investigated the effects of administering ART alone or in combination with Met on central SSN and SG in the T2DM rat model based on the PI3K/AKT pathway. Furthermore, we evaluated whether a combination of ART with Met would be more efficacious than administration of either drug alone with regard to diabetes-induced hyposalivation.

## Materials and Methods

### Animals

SPF male Sprague–Dawley rats aged 5 weeks (150–180 g) were obtained from the animal center of Guangxi Medical University (Nanning, China). All rats were housed under controlled conditions with room temperature at 22 ± 2°C, 12/12 h light/dark cycle, and 55 ± 5% relative humidity. The rats were acclimatized for 1 week by providing food and water ad libitum. All procedures involving animal experiments were approved by the Animal Ethics Committee of Guangxi Medical University (No. 202006013).

### Animal Grouping and Experimental Design

**FIGURE 1:**
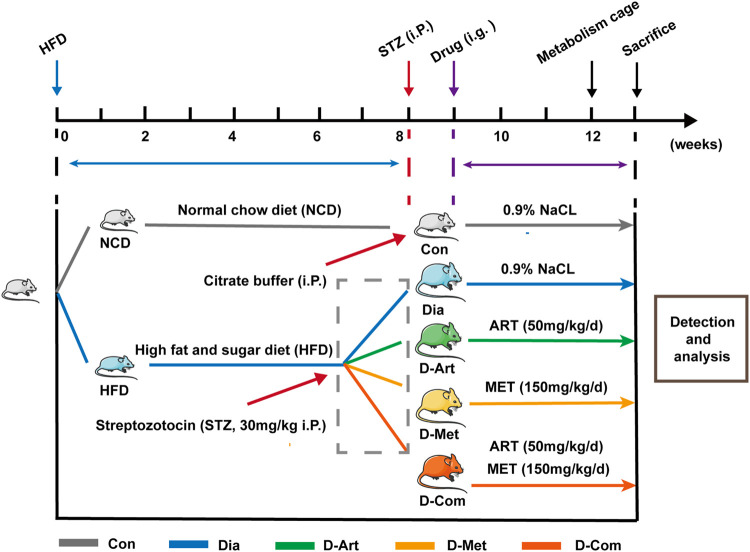
Experimental design. STZ was intraperitoneally injected at 8 weeks after NCD or HFD feeding. Rats with HFD/STZ treatment respectively received intragastric administration of ART, Met, and ART/Met combination for four consecutive weeks at 9 weeks after feeding. Animals were placed in metabolism cages, followed by measurement of metabolic parameters (urine output, food consumption, and water intake) at 12 weeks after feeding. Finally, rats were sacrificed at 4 weeks post drug treatment for various analyses. ART, artesunate; Met, metformin. Con, Normal control group; Dia, Untreated diabetic group; D-Art, ART-treated diabetic group; D-Met, Met-treated diabetic group; D-Com, ART/Met co-treated diabetic group.

The high-fat high-sugar diet (HFD, boaigang-B1135DM, Boaigang Biotechnology Co. Ltd., Beijing, China) and low dose of streptozotocin (STZ; Sigma, St. Louis, MO) were used to establish a rat model of type 2 diabetes mellitus (T2DM) according to a previously described protocol (Kelany et al., 2016; Nie et al., 2018). After acclimation for 1 week, the rats were randomly assigned into two different dietary formulas: standard chow diet and HFD diet post (Figure 1). All animals were fasted overnight (16 h) after 8 weeks of HFD-feeding. Rats in the HFD dietary group received a single dose of STZ (30 mg/kg body weight, BW) dissolved in a freshly prepared pH 4.2 citrate buffer by intraperitoneal injection (i.p), while an equivalent volume of citrate buffer was administered to the rats fed with a normal standard diet. Fasting blood glucose (FBG) was determined at the tail vein using Accu-Chek Active test strips (Roche Diabetes Care GmbH, Mannheim, Germany) at 3 and 7 days after STZ administration. Notably, rats with a FBG of >11.1 mmol/L were considered as T2DM models (Zeng et al., 2019). Next, T2DM rats were randomly divided into four different experimental groups (*n* = 8/group): Untreated diabetic group (Dia), ART-treated diabetic group (D-Art), Met-treated diabetic group (D-Met), and ART/Met co-treated diabetic group (D-Com). In addition, rats fed on the standard chow diet were classified into the normal control group (Con). One week after STZ injection, the treatments were administrated by gavage to rats in each group, once a day, consecutively for 4 weeks (Figure 1). Rats in ART-treated, Met-treated, and ART/Met co-treated diabetic groups were treated with ART alone (50 mg/kg; Guilin Pharmaceutical Co., Ltd., Guilin, China), Met alone (150 mg/kg; Shenzhen Neptune Pharmaceutical Co., Ltd., Shenzhen, China), and ART (50 mg/kg) combined with Met (150 mg/kg), respectively, whereas the non-diabetic control and untreated diabetic rats received equivalent doses of saline. Notably, the administered ART and Met doses were in accordance with previous studies (Cai et al., 2018; Bradley et al., 2019; Inyang et al., 2019; Chen et al., 2021).

### Evaluation of General Conditions

BW was measured weekly after drug treatments. Moreover, the 24 h urine volume, daily food intake, and daily water intake were examined using metabolic cages at 4 weeks post drug treatments.

### Measurement of FBG, Oral Glucose Tolerance and Insulin Resistance

FBG levels were detected post drug administration. Oral glucose tolerance test (OGTT) was performed to evaluate oral glucose tolerance of each group after 12 weeks. Animals were given intragastric administration of glucose solution (2 g/kg BW) following a 12 h fasting period. Blood glucose levels were respectively measured at 0, 30, 60, 90, and 120 min post glucose administration using the tail prick method. Serum insulin levels were assayed using the Rat Insulin (INS) ELISA Kit (Cat#CSB-E05070r, Cusabio Biotech Co. Ltd., Wuhan, China) according to the manufacturer’s instructions. Finally, homeostatic model assessment of insulin resistance (HOMA-IR), used to quantify insulin resistance, was calculated using the HOMA-IR formula: HOMA-IR = FBG (mmol/L) × Fasting blood insulin (μU/mL)/22.5.

### Measurement of Saliva Secretion

Saliva secretion was estimated at 4 weeks after drug treatment. The volume of saliva secretion was collected using several small degreased cotton balls and pieces according to a previously described protocol (Kojima et al., 2011). Briefly, rats were anesthetized with 1% sodium pentobarbital (40 mg/kg) and then subcutaneously injected with 0.2% pilocarpine (0.2 mg/kg). Next, pre-weighed small cotton balls were placed into the sublingual region of oral cavity post pilocarpine stimulation. After collection for 30 min, all cotton balls were removed from the oral cavity and immediately weighed using a weigh balance. Finally, saliva secretion was calculated and normalized to BW.

### Evaluation of Biochemical Parameters

Levels of serum lipids, including triglycerides (TG), total cholesterol (TC), low-density lipoprotein cholesterol (LDL-C), and high density lipoprotein cholesterol (HDL-C) were analyzed using the automatic biochemical analyzer (Mindray Co. Ltd., Shenzhen, China) at the end of experiment.

### Sample Collection and Preparation

On the final day of the experiment, animals were anaesthetized and blood samples were collected from the inferior vena cava, followed by centrifugation at 3,000 rpm for 10 min at 4°C. After centrifugation, the serum was retained for use in further corresponding analyses. Next, submandibular glands (SMGs), brainstem, and pancreas tissue samples of rats were quickly isolated, weighed, and rinsed after euthanasia. The samples were immediately placed into liquid nitrogen and then stored at −80°C or fixed in 4% paraformaldehyde solution until further analysis.

### Observation of Ultrastructure in SMG and SSN

At the end of the experiment, a transcardial perfusion using 2.5% glutaraldehyde and 4% paraformaldehyde mixed fixative in 0.1 M mol phosphate buffer (PB, pH 7.4) was performed on anesthetized rats. Next, SMG and SSG tissues were rapidly isolated and fixed in 2.5% glutaraldehyde at 4°C for 24 h, followed by 1% osmium tetroxide at 4°C for 3 h. After gradient alcohol dehydration, specimens were infiltrated and embedded using epoxy resin. A transmission electron microscope (TEM) (HITACHI H-7000, Tokyo, Japan) was then used to observe the ultrastructure in SMG and SSN tissues, including acinar cells, ductal cells, and secretory granules of the SMG and axon of the SSN. Moreover, at least 100 axon diameters of the SSN were determined in each group using ImageJ software in accordance with a previous study (Ito et al., 2016). Subsequently, the G-ratio was calculated using the following formula: G-ratio = inner axonal diameter (d)/(outer axonal diameter) (D), as shown in Figure 5C.

### Histopathological Analysis

The tissues (SMGs, pancreas, and brain stem) were fixed in 4% paraformaldehyde solution for 48 h, dehydrated, and embedded in paraffin. Paraffin-embedded specimens were cut into 4 μm-thick sections using paraffin slicing machine (Leica, Braunschweig, German). Sections obtained from SMGs and pancreas tissues were stained with hematoxylin and eosin (H&E) and Alcian Blue Periodic acid Schiff (AB-PAS), while brain tissue sections were stained with H&E and Nissl. Finally, the sections were observed and images were captured using an optical microscope (Olympus, Tokyo, Japan). According to published studies (Sefi et al., 2011; Huang et al., 2018b; Tian et al., 2021), quantitative analyses of histology in the pancreas, SMGs and SSN were respectively performed using ImageJ software. Notably, five different images were captured per rat and at least three different rats were used from each group for comparative analysis.

### Immunohistochemistry and Immunofluorescence Staining

Tissue sections (4 μm-thick) were dewaxed in dimethylbenzene, followed by high-pressure treated antigen retrieval for 5 min in a pH 6.0 citrate buffer heated at boiling temperature. Next, sections were immersed into 3% hydrogen peroxide to block endogenous peroxidase for 15 min and then washed three times in a pH 7.4 phosphate buffer saline (PBS, 0.01 mol/L) for 5 min each time. After blocking in serum buffer at room temperature for 20 min, the sections were incubated with the primary antibodies, including anti-aquaporin5 (AQP5) (1:250, ab78486, Abcam, Cambridge, United Kingdom) and anti-acetylcholinesterase (AChE) (1:200, Bioss Biological Technology Co., Ltd., Beijing, China) at 4°C overnight. Next, sections were rinsed in PBS three times for 5 min each time, followed by incubation with HRP-conjugated or fluorescence labeled (Alexa Fluor 594® anti-rabbit IgG, Cell Signaling Technology, Danvers, MA, United States) secondary antibodies at room temperature for 1 h. The sections were then respectively stained with 3,3′-diaminobenzidine (DAB) or counterstained with DAPI. The stained sections were observed and images captured using an optical microscope or a laser scanning confocal microscope (Olympus, Tokyo, Japan). Finally, the images were evaluated using Image-Pro Plus 6.0 Software (Media Cybernetics, CA, United States) according to a protocol described in a previous study (Li et al., 2020). The mean density was calculated as follows: mean density = integral optical density (IOD)/area. Notably, the reported values correspond to the average of at least five representative images obtained from three different animals from each group.

### Terminal Deoxynucleotidyl Transferase-Mediated dUTP-Biotin Nick End Labeling

TUNEL staining in SMG was conducted using *In Situ* Cell Death Detection Kit (Roche, Mannheim, Germany), according to manufacturer’s instruction. Apoptosis levels in each group were evaluated by counting TUNEL positive cells with ImageJ.

### Quantification of Brain-Derived Neurotrophic Factor in SMG Using Enzyme-Linked Immunosorbent Assay

Tissue samples were centrifuged at 12,000 rpm for 10 min at 4°C to collect supernatants, followed by assessment of BDNF levels in SMG tissues using rat ELISA kits according to the manufacturer’s instructions (Cat#D731142, Sangon Biotech Co. Ltd., Shanghai, China). Next, the resulting colors were quantified using a plate reader (Bio Tech Instruments, United States) at 450 nm.

### Western Blot Analysis

Protein samples were extracted using RIPA lysis buffer with phosphatase and protease inhibitors according to the manufacturer’s protocol. Western blot analysis was conducted according to a protocol described in a previous study (Ferenczyova et al., 2020). Briefly, non-closure sodium dodecyl sulfate polyacrylamide gel electrophoresis (SDS-PAGE) color preparation kits (Cat#C681100, C681101, and C681103, Sangon Biotech Co., Ltd., Shanghai, China) were used to resolve the protein samples extracted from all groups. Next, protein samples were transferred to polyvinylidene difluoride (PVDF) membranes using the wet electrotransfer method. Non-specific binding sites of the membranes were blocked with 5% non-fat milk, followed by incubation with primary antibodies at 4°C overnight. Membranes were then incubated with secondary antibodies in a swing bed at room temperature for 1 h. Finally, enhanced chemiluminescence substrate (ECL) (Cat#BL520, Lanjike Technology Co., Ltd., Biosharp, Hefei, China) was used to detect horseradish peroxidase (HRP) signal.

### Molecular Docking of ART to Akt and PI3K Protein

Firstly, the three-dimensional structure of ART molecule in Conformer SDF format (Compound CID: 6917864) was obtained from the NCBI database (https://pubchem.ncbi.nlm.nih.gov/), followed by construction of the homology model of Akt (PDB ID: 2UZT) and PI3K (PDB ID: 1E7V) protein molecules based on the Protein Data Bank (PDB) database (https://www.rcsb.org/). Next, desolventization of above molecules was performed using the PyMOL software in accordance with the published literatures. The AutoDock1.5.6 software was then used to conduct molecular dockings, specifically setting the docking algorithm to Lamarckian Genetic Algorithm. Finally, the binding site was defined by selecting the appropriate size around the bound co-crystal ligand.

### Statistical Analysis

All statistical analyses were performed using GraphPad Prism 7.0 software (GraphPad Software Inc. San Diego CA, United States). The results were expressed as mean ± standard deviation (SD). One-/two-way analysis of variance (ANOVA) followed by Tukey’s multiple comparison tests were used to compare differences among groups. *p* < 0.05 was considered statistically significant.

## Results

### ART or Met Alone and ART/Met Combination Alleviate General Conditions in the T2DM Rats

**FIGURE 2:**
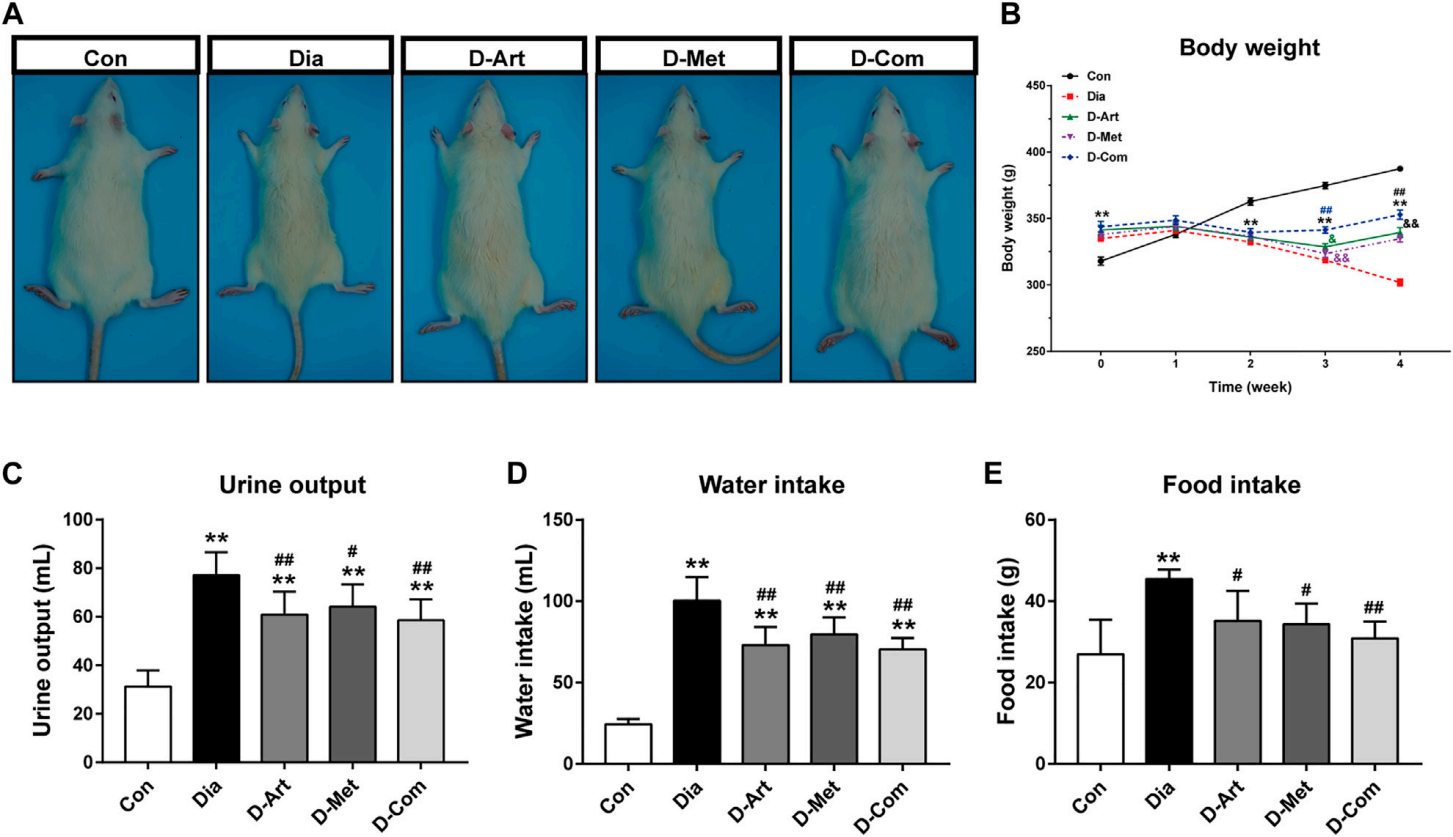
ART or Met alone and ART/Met combination alleviate general conditions in the T2DM rats. **(A)** Gross appearance of each group of rats. **(B–E)** Body weight, urine output, water intake, and food intake in rats of each group (8 rats per group). Data are expressed as the mean ± SD. **p < 0.01 versus Con group; #*p* < 0.05 and ##*p* < 0.01 versus Dia group. Con, Normal control group; Dia, Untreated diabetic group; D-Art, ART-treated diabetic group; D-Met, Met-treated diabetic group; D-Com, ART/Met co-treated diabetic group.

To investigate the effects of ART alone or in combination with Met on general conditions in T2DM rats, the BW of rats was measured every week after drug administration, and urine output, water intake, and food intake were measured 1 week before the terminal experiment. Results showed that BW in the untreated and drug-treated diabetic groups was significantly increased compared to the control group after 8 weeks of HFD feeding, while weight loss was observed in the untreated and drug-treated diabetic groups from 3 weeks post streptozotocin (STZ) administration (Figure 2B). ART/Met combination significantly increased BW of diabetic rats from 3 weeks after drug administration, while ART or Met alone significantly increased BW from 4 weeks after drug administration. In addition, BW was lower in rats receiving ART or Met alone treatment than in rats with ART/Met combination at 3 weeks after drug administration, and no significant difference in BW was observed between the ART- and Met-treated groups. At the end of the experiment, an obvious decrease in BW (Figures 2A,B), and a significant increase in urine output (Figure 2C), and food and water intake (Figures 2D,E) were observed in the untreated diabetic rats compared to the control rats. Conversely, the drug-treated (ART, Met, and ART/Met combination) diabetic rats showed a significant decrease in urine output, food consumption, and water intake compared to the untreated diabetic rats, and there was no significant difference in these parameters among the three treatment groups.

### ART or Met Alone and ART/Met Combination Alleviate Typical T2DM Characteristics and Pancreatic Injury in the T2DM Rats

**FIGURE 3:**
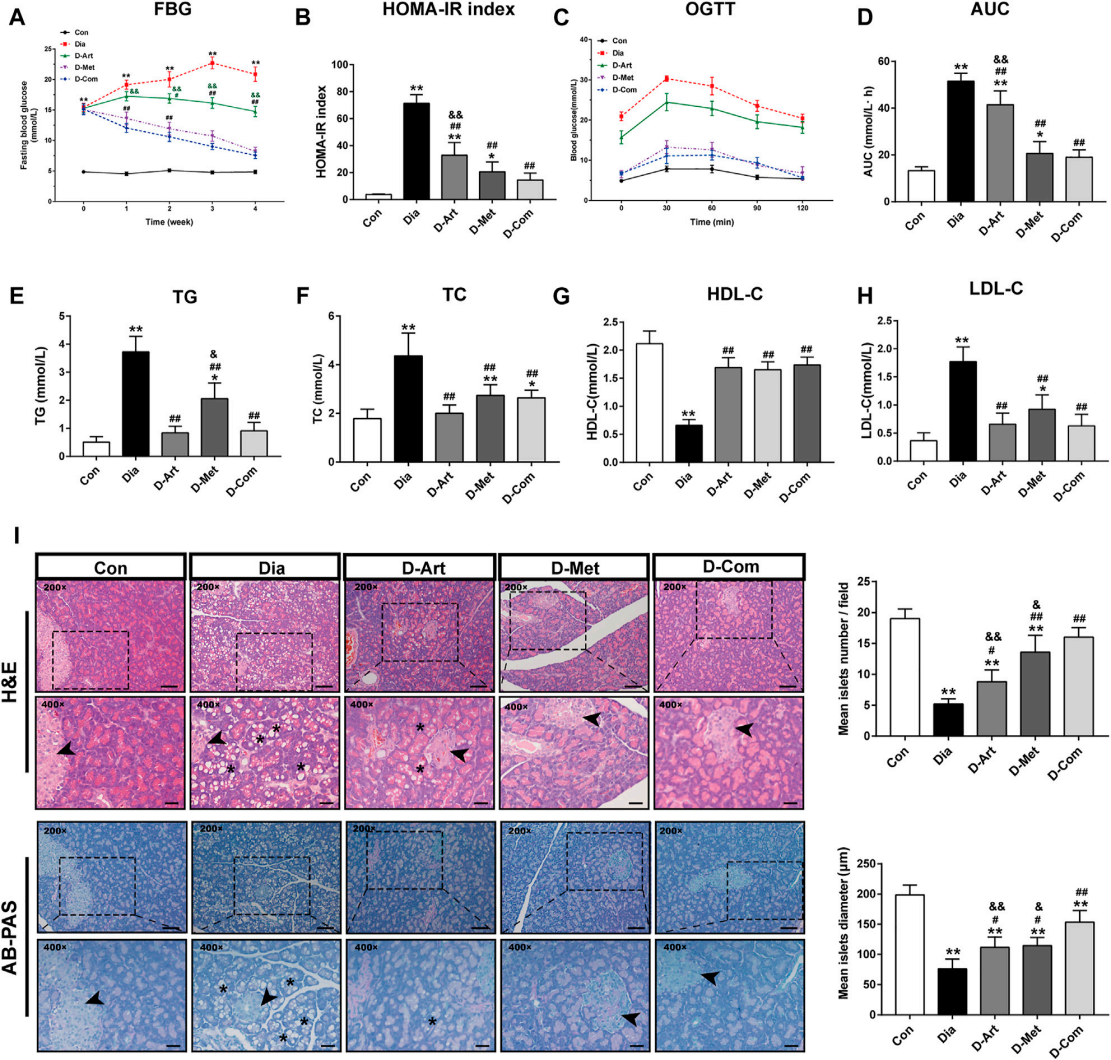
ART or Met alone and ART/Met combination alleviate typical T2DM characteristics and pancreatic injury in the T2DM rats. **(A)** FBG, **(B)** HOMA-IR index, **(C)** OGTT, **(D)** AUC of the OGTT, **(E)** TG, **(F)** TC, **(G)** HDL-C, and **(H)** LDL-C of rats in each group (8 rats per group). **(I)** H&E and AB-PAS staining of pancreas tissues in each group (5 rats per group), scale bar = 50 μm (200×); scale bar = 20 μm (400×). Pancreas islet sites are labeled using black arrows, while vacuolation is labeled using black *. Data are expressed as the mean ± SD. *p < 0.05 and **p < 0.01 versus Con group; #*p* < 0.05 and ##*p* < 0.01 versus Dia group; &*p* < 0.05 and &&*p* < 0.01 versus D-Com group. OGTT, oral glucose tolerance test; AUC, area under the curve; TG, total glyceride; TC, total cholesterol; LDL-C, low-density lipoprotein cholesterol; HDL-C, high-density lipoprotein cholesterol; H&E, Hematoxylin & eosin; AB-PAS, Alcian Blue Periodic acid Schiff. Con, Normal control group; Dia, Untreated diabetic group; D-Art, ART-treated diabetic group; D-Met, Met-treated diabetic group; D-Com, ART/Met co-treated diabetic group.

To examine whether ART alone or in combination with Met could ameliorate hyperglycemia, hyperlipidemia, insulin resistance and pancreatic injury in T2DM rats, we assessed glycemic control, glucose tolerance (OGTT), insulin resistance (HOMA-IR), serum lipid profiles (TG, TC, LDL-C, and HDL-C), and pancreas histology post drug administration. Results showed that FBG levels were markedly increased in diabetic rats compared to the Con group from 1 week after STZ injection (Figure 3A). A significant decline over time in FBG level was observed in three drug treatment groups. The results also indicated that ART significantly diminished FBG levels compared to the untreated diabetic group 2 weeks post drug administration, while Met alone and ART/Met combination decreased FBG levels 1 weeks post drug administration. However, there was no significant difference in FGB level between the Met treated group and the ART/Met co-treated group. Moreover, the HOMA-IR index and glucose area under the curve (AUC) for the OGTT value were significantly higher in diabetic rats than in non-diabetic control rats (Figures 3B–D). In contrast, the HOMA-IR index and glucose AUC were significantly reduced after ART, Met, and ART/Met combination treatments compared to the untreated group. Notably, HOMA-IR index and glucose AUC were significantly higher in ART-treated group rats than in the ART/Met co-treated group, while no significant difference was observed between the Met-treated and ART/Met co-treated groups (Figures 3B–D). In addition, diabetic rats in the untreated and Met-treated groups exhibited an obvious elevation in serum TG, TC, and LDL-C levels (Figures 3E,F,H), whereas the only untreated diabetic rats showed a reduction in HDL-C level (Figure 3G) compared to the control group rats. Conversely, rats from the three treatment groups showed a significant decrease in serum TG, TC, and LDL-C levels, and an increase in the serum HDL-C levels, but no significant difference was observed among the three treatment groups in serum TC, HDL-C, and LDL-C levels (*p* > 0.05).

Figure 3I showed the pancreatic histopathology results. Pancreatic histology in the untreated diabetic group showed a large number of diffuse vacuoles in pancreatic cells, a significant reduction in islet number and diameter, compared to the normal control group. However, these abnormal histopathological changes were significantly rescued by ART, Met, and ART/Met combination treatments, particularly in the ART/Met combination treatment group.

### ART or Met Alone and ART/Met Combination Mitigate SGs’ Dysfunction and Pathological Alterations in the T2DM Rats

**FIGURE 4:**
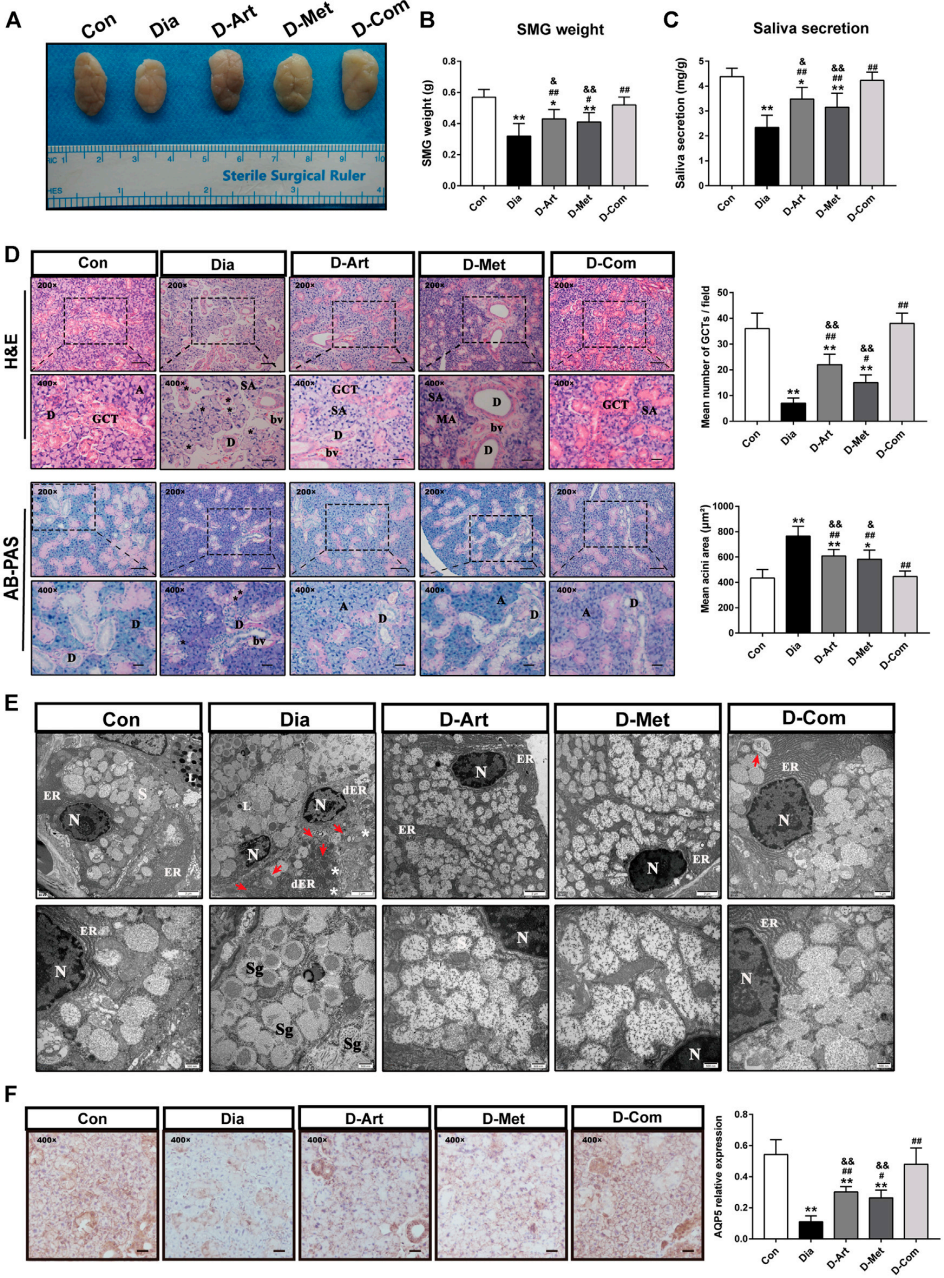
ART or Met alone and ART/Met combination mitigate SGs’ dysfunction and pathological alterations in the T2DM rats. **(A)** Gross morphology and **(B)** weight of SMGs in each group. **(C)** Saliva secretion of rats in each group (8 rats per group). **(D)** H&E and AB-PAS staining of SMGs in each group (5 rats per group), scale bar = 50 μm (200×); scale bar = 20 μm (400×). A, acini; SA, serous acini; MA, mucous acini; D, duct; GCT, granular convoluted tubules; bv, blood vessel. Vacuolation is labeled using black*. **(E)** Ultrastructure of SMGs in each group. N, nucleus; ER, endoplasmic reticulum; dER, disrupted endoplasmic reticulum; L, Lysosome; Sg, secretory granules. White * represent swollen and disrupted mitochondria and red arrows represent autophagosomes. **(F)** Immunohistochemistry analysis of AQP5 expression in SMGs of each group, scale bar = 20 μm (400×). Data are expressed as the mean ± SD. *p < 0.05, **p < 0.01 versus Con group; #*p* < 0.05, ##*p* < 0.01 versus Dia group; &*p* < 0.01, &&*p* < 0.01 versus D-Com group. H&E, Hematoxylin & eosin; AB-PAS, Alcian Blue Periodic acid Schiff. Con, Normal control group; Dia, Untreated diabetic group; D-Art, ART-treated diabetic group; D-Met, Met-treated diabetic group; D-Com, ART/Met co-treated diabetic group.

To investigate effects of ART/Met on SGs’ function and pathological alterations in T2DM rats, we measured saliva secretion, SMG’s weight, morphology, and ultrastructure of SMG. The rats from the Dia, D-ART and D-Met groups showed reduction of saliva secretion and SMG weight compared to the normal control group rats (Figures 4A–C). In addition, no obvious difference in saliva secretion and SMG weight was observed between the ART/Met co-treated and normal control groups. However, we noticed that treatment with ART or Met alone and the ART/Met combination markedly recovered saliva secretion and increased SMG weight when compared to the untreated diabetic group, among of which ART/Met combination exhibited the best effect.

H&E staining showed that SMG of the control group had a normal duct and acini structure, with neat arrangement. However, an obvious increase of acinus area and a decrease of granular convoluted tubules (GCT) number, and fibrosis around the ducts were observed in the SMG of T2DM rats (Figure 4D). The above abnormal changes were mitigated after ART, Met, and ART/Met combination treatments, with ART/Met combination exhibiting better effects than the other two treatment. Moreover, AB-PAS staining was used to determine changes in acidic mucin (colored blue) and neutral mucin (colored red) (Figure 4D). The AB-PAS staining results showed that the SMG of untreated diabetic group rats exhibited stronger staining with purple-red acini and deep blue nucleus than that of the control group, suggesting that more acidic and neutral mucin accumulated in the acini of diabetic SMG. However, the SMG of the three treatment groups were lightly stained by the AB-PAS reagents compared to SMG of the untreated diabetic group. In addition, the ultrastructure of SMG was examined using a TEM (Figure 4E). The GCT and acinar cells in the control group possessed round nucleus, clear nuclear membrane, round secretory granules, and well-developed organelles, such as rough endoplasmic reticulum, high electron density lysosomes, and mitochondria. In contrast, nuclear pyknosis, autophagosomes, disrupted endoplasmic reticulum, and swollen and disrupted mitochondria were observed in the untreated diabetic group compared to the control group. However, the three treatments alleviated the above impaired changes in the acinus of diabetic rats. Furthermore, more developed endoplasmic reticulum was observed in the ART/Met treated group compared to that in the ART- and Met-treated groups.

Besides, AQP5 is the most important aquaporin for saliva secretion (D'Agostino et al., 2020). Accordingly, we also evaluated AQP5 expression in SMGs (Figure 4F). Results also demonstrated that expression of AQP5 in the Dia group was obvious lower than the Con group. However, these three treatments upregulated AQP5 expression in the T2DM rats when compared with the Dia group. Furthermore, we noticed that the ART/Met combination had the best effect among the three treated group.

### ART or Met Alone and ART/Met Combination Alleviate Central SSN Injury and Preserved Peripheral Parasympathetic Innervation of SGs in the T2DM Rats

**FIGURE 5:**
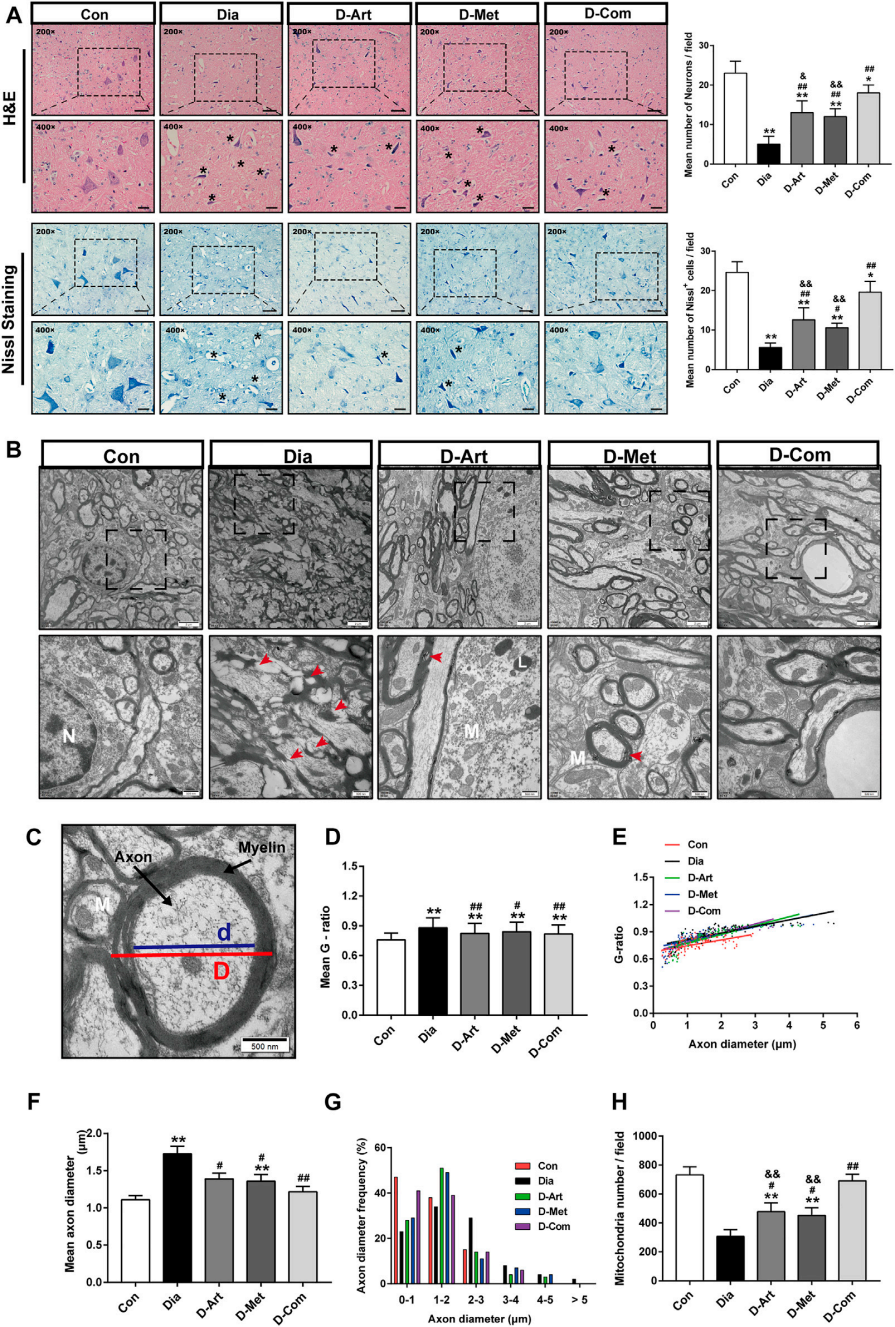
ART or Met alone and ART/Met combination alleviate central SSN injury in the T2DM rats. **(A)** H&E and Nissl staining of SSN in each group (5 rats per group), scale bar = 50 μm (200×); scale bar = 20 μm (400×). Crumpled and vacuolated neurons were labeled using black *. **(B)** Ultrastructure of SSN in each group (3 rats per group). N, nucleus; L, Lysosome; M, mitochondria. Red arrows represent damaged and disrupted myelin. **(C)** Schematic diagram of G-ratio. **(D–H)** The mean g-ratios, individual g-ratios distribution, axonal diameters, and distributions of axonal diameters, and mitochondria number in different groups. SSN, superior salivatory nucleus; Data are expressed as the mean ± SD. *p < 0.05 and **p < 0.01 versus Con group; #*p* < 0.05, ##*p* < 0.01 versus Dia group; &*p* < 0.01 and &&*p* < 0.01 versus D-Com group. Con, Normal control group; Dia, Untreated diabetic group; D-Art, ART-treated diabetic group; D-Met, Met-treated diabetic group; D-Com, ART/Met co-treated diabetic group.

It is well known that SSN innervates saliva and tear secretion (Ramos et al., 2020). To investigate the mechanism through which ART alone or in combination with Met mitigated diabetes-induced hyposalivation, we first conducted histological analysis (HE and Nissl staining) and ultrastructure examination in SSN. HE staining of SSN in the control group showed normal cell shape, well-defined and clear nuclei (Figure 5A). In contrast, markedly neuronal loss, and degenerated neurons with pyknosis and even vacuolization were observed in the untreated diabetic rats compared to that of the normal control group. However, the number of neurons was significantly increased after ART, Met, and ART/Met combination treatments, especially in the ART/Met combination group. Moreover, Nissl staining also confirmed that the density of Nissl bodies in the untreated diabetic group was obviously less than that in the normal control group. However, ART/Met combination treatment significantly increased density of Nissl bodies in the SSN of diabetic rats compared to the untreated diabetic rats and respective monotherapies. Furthermore, damaged axons, disrupted myeline, and decreased mitochondrion were observed in the diabetic rats (Figures 5B,H). In addition, mean axon diameter and G-ratio were significantly higher in the untreated diabetic group than those in the normal control group (Figures 5C–G). However, three drug treatments improved abnormal morphology of axons and myelin, significantly increased mitochondrion density, and reduced mean axon diameter and G-ratio, with ART/Met combination being superior to the other two monotherapies in increasing mitochondrion density.

**FIGURE 6:**
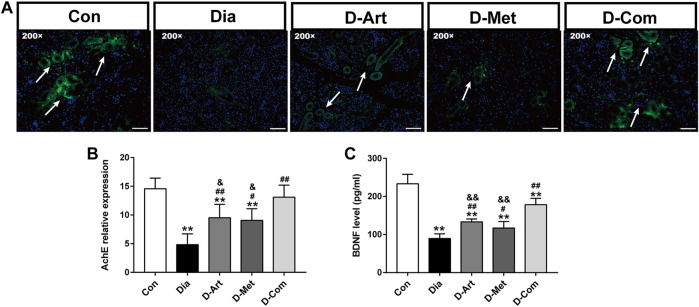
ART or Met alone and ART/Met combination preserved peripheral parasympathetic innervation of SGs in the T2DM rats. **(A)** Immunofluorescence of AChE in SMGs of each group, scale bar = 50 μm (200×). **(B)** Level of AChE expression in SMGs of each group. **(C)** ELISA analysis of BDNF in SMGs of each group (5 rats per group). Data are expressed as the mean ± SD. **p < 0.01 versus Con group; #*p* < 0.05 and ##*p* < 0.01 versus Dia group; &*p* < 0.05 and &&*p* < 0.01 versus D-Com group. Con, Normal control group; Dia, Untreated diabetic group; D-Art, ART-treated diabetic group; D-Met, Met-treated diabetic group; D-Com, ART/Met co-treated diabetic group.

Besides, evidence suggests that parasympathetic nerves from central SSN are closely associated with saliva secretion and regeneration of SGs after injury (Mitoh et al., 2017; Kim et al., 2020). It has been reported that BDNF promotes neural renewal (Knox et al., 2013), and AChE is known as an important marker of parasympathetic innervation. Therefore, we further examined the expression of AChEand BDNF in SGs. We observed that AChE and BDNF expression levels were reduced in the untreated diabetic group compared to the normal control group (Figures 6A–C). However, ART, Met, and ART/Met treatments significantly increased AChE and BDNF expression, and their expression levels were highest in the ART/Met co-treated group among the three treatment groups.

### ART or Met Alone and ART/Met Combination Activate PI3K/AKT Pathway in SMGs of the T2DM Rats

**FIGURE 7:**
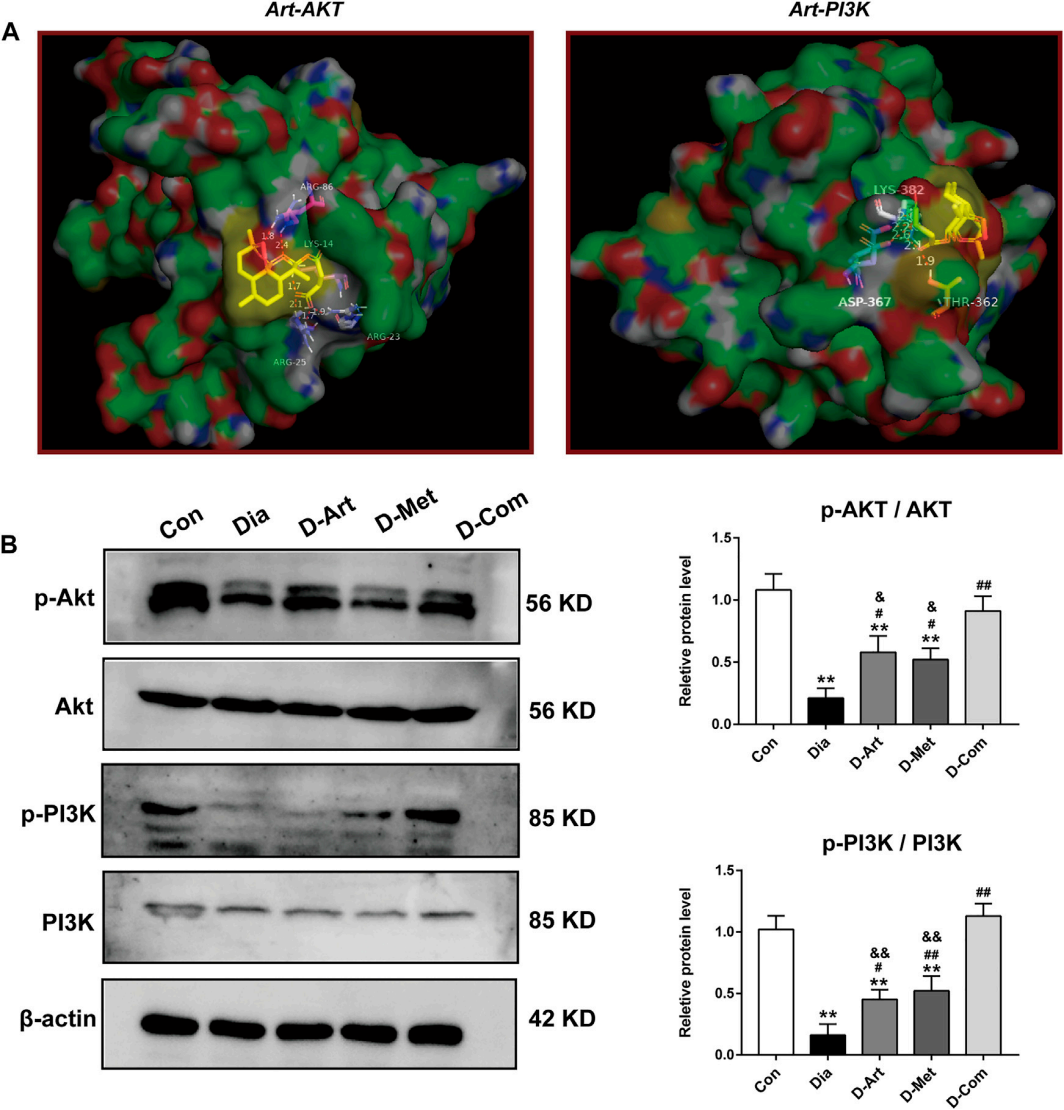
ART or Met alone and ART/Met combination activate PI3K/AKT pathway in SMGs of the T2DM rats. **(A)** Molecular docking of ART to Akt and PI3K protein. **(B)** Western blot analysis of p-AKT/AKT and p-PI3K/PI3K (5 rats per group). Protein expression is normalized to β-actin. Data are expressed as the mean ± SD. **p < 0.01 versus Con group; #*p* < 0.05 and ##*p* < 0.01 versus Dia group; &*p* < 0.05 and &&*p* < 0.01 versus D-Com group. Con, Normal control group; Dia, Untreated diabetic group; D-Art, ART-treated diabetic group; D-Met, Met-treated diabetic group; D-Com, ART/Met co-treated diabetic group.

To further explore the molecular mechanism of ART/Met effect on diabetes-induced SMGs damage, we assessed the proteins that are involved in the PI3K/AKT pathway, which has been shown to be highly associated with T2DM (Huang et al., 2018a). Molecular docking results showed that ART formed hydrogen bonds with amino acid residues at the active site of AKT (ARG-23 ARG-25, ARG-86, and LYS-14) and PI3K (LYS-382, ASP-367, and THR-362), with optimal conformational binding energies of −9.32 and −6.07 kcal/mol, respectively (Figure 7A). This suggested that the PI3K/AKT pathway had binding sites for ART. Moreover, western blot results showed that expression ratios of p-Akt/Akt and p-PI3K/PI3K in the untreated diabetic group was significantly lower than in the Con group (Figure 7B). However, the three treatment groups obviously upregulated p- AKT/AKT and p-PI3K/PI3K expression when compared to the Dia group. In addition, we noticed that expression of p-AKT/AKT and p-PI3K/PI3K in the D-ART and D-Met groups were markedly lower than that of the D-Com group.

### ART or Met Alone and ART/Met Combination Inhibit Apoptosis and Autophagy in the SMGs of T2DM Rats

**FIGURE 8:**
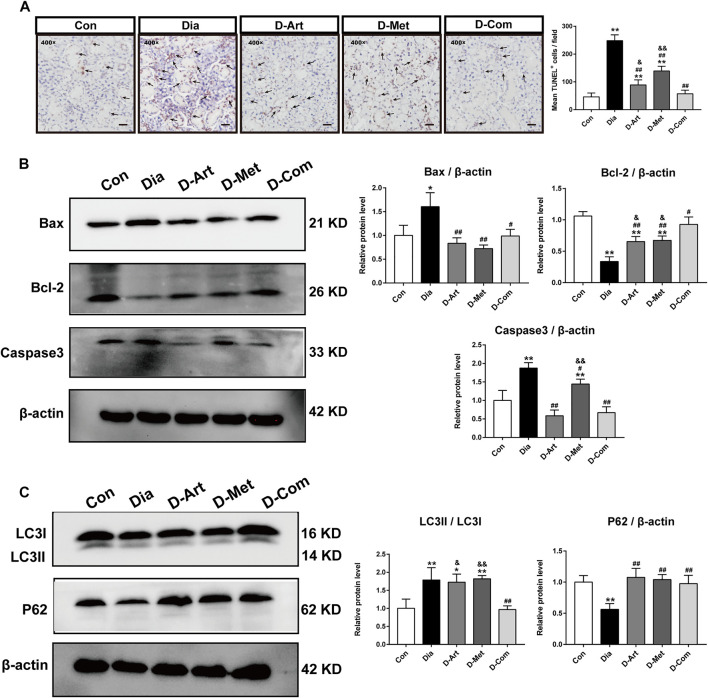
ART or Met alone and ART/Met combination inhibit apoptosis and autophagy in the SMGs of T2DM rats. **(A)** TUNEL staining in SMGs. Black arrows display TUNEL positive cells. **(B)** Western blot analyses for Bax, Bcl-2, and Caspase3 protein expression in SMGs. **(C)** Western blot analyses for LC3 and P62 protein expression in SMGs (5 rats per group). Protein expression is normalized to β-actin. Data are expressed as the mean ± SD. *p < 0.05 and **p < 0.01 versus Con group; #*p* < 0.05 and ##*p* < 0.01 versus Dia group; &*p* < 0.05 and &&*p* < 0.01 versus D-Com group. Con, Normal control group; Dia, Untreated diabetic group; D-Art, ART-treated diabetic group; D-Met, Met-treated diabetic group; D-Com, ART/Met co-treated diabetic group.

Previous studies have reported that the PI3K/Akt pathway can inhibit apoptosis and autophagy (Yang et al., 2018; Wang et al., 2019). Therefore, we employed TUNEL staining and Western blot to investigate whether these drug treatments could restrain apoptosis and autophagy in the SMGs with T2DM. TUNEL staining displayed that number of TUNEL^+^ cells in the Dia group were obviously more than that in the Con group (Figure 8A). However, increased TUNEL^+^ cells in the Dia group were inhibited by treatments with ART or Met alone and ART/Met combination, among of which ART/Met combination exhibited the best suppressive effect in TUNEL^+^ cells increase (*p* < 0.01; Figure 8A). Furthermore, expression levels of typical apoptosis-related markers (Bax, Caspase3, and Bcl-2) (He et al., 2021), were evaluated using Western blot. The results showed that the expression levels of Bax and Caspase3 protein in the untreated diabetic group were upregulated (*p* < 0.01; Figure 8B), while expression level of Bcl-2 protein was downregulated compared to the normal control group (*p* < 0.01; Figure 8B). Conversely, a significant increase in expression of Bcl-2 protein, and a reduction in Bax and Caspase3 expression were observed after ART, Met, and ART/Met combination treatments (*p* < 0.05 or 0.01; Figure 8B). Notably, ART/Met combination was superior to the other two treatments in upregulating expression of Bcl-2 protein.

Furthermore, the two main indicators of autophagy (P62 and LC3) were also detected using western blot analysis. Results showed that the expression ratio of LC3I/LC3II protein in the untreated diabetic group were markedly increased compared to that in the normal control group, whereas the expression level of P62 protein was significantly reduced (*p* < 0.01; Figure 8C). In contrast, ART, Met, and ART/Met combination treatments significantly upregulated the expression of P62 protein, while only ART/Met combination treatment markedly downregulated the expression ratio of LC3I/LC3II protein in diabetes rats (*p* < 0.01; Figure 8C). Moreover, the expression ratio of LC3I/LC3II protein was significantly lower in the ART- and Met-treated groups than that in the ART/Met co-treated group.

## Discussion

In our present study, the T2DM rat model was successfully established by using HFD and STZ, and was characterized by weight loss, increased water intake and urine output, hyperglycemia, hyperlipemia, insulin resistance, and pancreatic injury. Results showed that ART, Met, and ART/Met combination treatments significantly attenuated these typical characteristics of T2DM. Besides, we noticed that ART/Met combination had a superior effect than ART alone in controlling blood glucose levels, regulating glucose tolerance, and improved insulin resistance. Interestingly, we also observed that the above typical T2DM parameters in the rats with ART/Met combination treatment were comparable to those of the control group, whereas this effect was not achieved in the monotherapies. These results revealed that ART/Met combination treatment might possess more effective in the future in preventing development and progression of T2DM than ART or Met alone. In addition, we observed that saliva secretion and SMG weight in the Dia, D-ART and D-Met groups were significantly reduced compared to the Con group. We also employed HE staining, AB-PAS staining and TEM analysis to evaluate histology and ultrastructure of SMGs. As shown in the Figures 4D,E, diabetes-induced SMGs’ pathological alterations. Taken together, our data demonstrated that DM contributed to SGs’ hyposalivation and damage, which is consistent with results reported by Xiang et al. (2020). As main transporter protein mediating saliva secretion, AQP5 is recognized as a potential target for the treatment of xerostomia (D’Agostino, C. 2020). Accordingly, we used immunohistochemistry staining of AQP5 to further confirm that hyperglycemia induced SGs’ dysfunction (Figure 4F). Subsequently, it was observed that after treatments with ART, Met and ART/Met combination reduction of saliva production and injury of SMGs were alleviated in the T2DM rats. Notably, ART/Met combination administration was not only superior to monotherapies but also comparable to rats in the control group in restoring saliva secretion and alleviating SMGs damage. These findings suggest that ART/Met could ameliorate diabetes-induced dysfunction. However, the underlying mechanisms were not elucidated.

DM is a chronic metabolic disease characterized by hyperglycemia (Öztürk et al., 2017) and can damage multiple organs, among which, the brain is one of most common target organs in DM complications (Hamed, 2017; Zhao et al., 2019). Thus, SSN in the brainstem is unlikely to be immune to the damage caused by chronic hyperglycemia. It has been reported that the expression level of AQP5 in SMG is regulated by the parasympathetic nerve (Hosoi et al., 2020). Parasympathetic nerves are closely associated with SGs regeneration, and intact parasympathetic innervation facilitates SMGs regeneration (Wang et al., 2021). Given that the origin of the parasympathetic preganglionic is neurons, SSN plays a key role in innervating lacrimal gland and SMG (Mitoh et al., 2017). Previous studies have reported that parasympathetic denervation give rise to loss in SMG weight and decrease in AQP5 expression (Azlina et al., 2010; Wang et al., 2021), which was also observed in our study (Figures 4B,F). Therefore, we speculated that hyperglycemia may not only cause SMG injury, but also induce SSN damage, both of which lead to diabetes xerostomia.

To further clarify our speculation, we first carried out histological staining (HE and Nissl staining) in SSN. HE staining showed that SSN of diabetic rats had obvious pathological alterations, such as neurons vacuolization, neurons loss and karyopycnosis, which were consistent with Nissl staining result (Figure 5A). These results revealed that hyperglycemia resulted in SSN pathological injury, further confirmed by the ultrastructure observation of SSN. As shown in Figures 5B–H, a significant reduction in mitochondrion number, and an increase in axon diameter and G-ratio of untreated diabetic rats were observed through TEM analysis of SSN, suggesting that diabetes led to injury of SSN neurons and axons. Axons are the crucial output channels of excitatory impulses between a neuron and its target effector, such as muscle and salivary gland cells (Zhou et al., 2020). Notably, we observed that these pathological alterations of SSN in the T2DM rats were markedly mitigated after treatments with ART, Met and ART/Met combination, with ART/Met combination outperforming ART and Met alone in terms of alleviating neurons and mitochondrion loss.

Besides, previous studies have reported the important effects of BDNF, as a salivary trophic factor, on preservation of parasympathetic innervation in SGs post irradiation (Hu et al., 2018; Kim et al., 2020). Nevertheless, diabetes and its various complications generally lead to a decrease in BDNF level (Chan et al., 2019; Kim and Song, 2020). AChE has also been reported to be a marker of parasympathetic innervation (Hu et al., 2018). Thus, we detected expression levels of AChE and BDNF to evaluate peripheral parasympathetic innervation in SMGs of T2DM rats following drug administration respectively using immunofluorescence and ELISA (Figure 6). Results showed that AChE and BDNF levels were significantly decreased in SMG of the untreated diabetes rats compared to the control group. As expected, reduction of these indicators in the SMG of diabetic rats were improved by the ART, Met, and ART/Met combination therapies. Interestingly, treatment with ART/Met combination displayed superior effects in inhibiting reduction of AChE and BDNF expression in SMG with T2DM than the respective monotherapies. Collectively, these data suggested that ART/Met might preserve peripheral SGs’ parasympathetic innervation to attenuate hyperglycemia-induced SG dysfunction through rescuing SSN injury.

Next, the possible molecular mechanisms through which ART/Met ameliorates the SGs injury induced by diabetes were further explored. Evidence suggests that the PI3K/Akt signaling pathway can regulate glucose homeostasis, inhibit apoptosis, and promote cell survival through multiple pathways (Huang et al., 2018a). The regulatory role of ART on the PI3K/Akt pathway has also been reported in previous studies (Feng and Qiu, 2018; Zhang et al., 2020), and it was further demonstrated by the molecular docking analysis of ART with PI3K and Akt conducted in this study. Huang et al. (2018b) reported that high glucose induced autophagy, thereby leading to AQP5 degradation in the diabetic SMG via the PI3K/AKT pathway, which is consistent with results obtained in this study (Figures 4F, 7, 8C). Subsequent western blot analysis results showed that the relative expressions of p-Akt, and p-PI3K proteins were upregulated in SGs after ART or Met alone and ART/Met combination, with ART/Met combination exhibiting the best effect among the three therapies. This suggested that the PI3K/Akt pathway was activated in the SGs of diabetic rats by the three treatments, especially ART/Met combination therapy. Previous studies have reported that the PI3K/Akt pathway is a vital pathway in the suppression of downstream apoptosis and autophagy (Tan et al., 2020; Tungsukruthai et al., 2021). Subsequently, we carried out TUNEL staining to determine whether ART alone and in combination with Met could mitigate apoptosis in SMGs with T2DM. As shown in TUNE staining (Figure 8A), ART, Met and ART/Met combination prominently ameliorated increase of TUNEL^+^ cells when compared to that of the untreated diabetic group. Besides, we found that number of TUNEL^+^ cells in SMG of the D-Com group was significantly lower than the two other monotherapy groups. Notably, Bax (apoptosis regulator), Bcl-2 (anti-apoptotic protein), and Caspase3 (pro-apoptotic protein) are key indicators of apoptosis (Ji et al., 2021), while LC3II/LC3I and P62 are markers of autophagy (Komatsu and Ichimura 2010; Yue et al., 2019). Thus, this study also examined expression of these apoptosis and autophagy-related proteins in SMGs using the western blot method. Results demonstrated that treatments with ART, Met and ART/Met combination upregulated protein expression of Bcl-2 and P62 compared to those of the Dia group, and reduced protein expression of Bax, Caspase3. We noticed that ART/Met combination treatment group had higher expression level of Bcl-2 protein in comparison with the D-ART and D-Met groups. However, simply administration with ART/Met combination significantly expression of LC3II/LC3I protein compared with the Dia group. Collectively, these results suggest that ART/Met combination treatment mitigated diabetes-induced SMG damage via activating the PI3K/AKT pathway to inhibit apoptosis and autophagy in SMGs.

**FIGURE 9:**
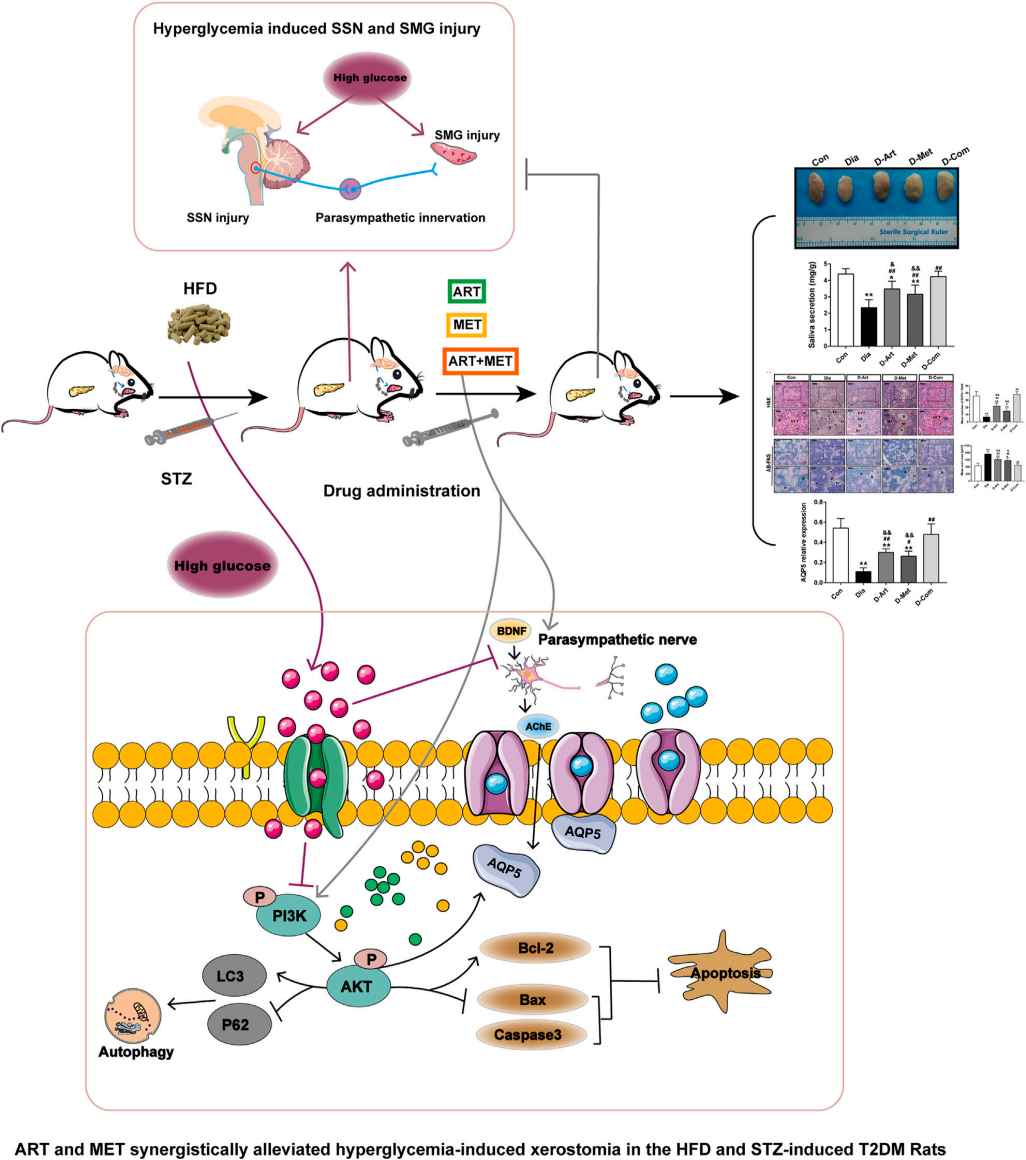
Schematic diagram of the proposed mechanisms through which ART and Met synergistically ameliorate xerostomia in HFD/STZ-induced T2DM rats via rescuing central SSN and SMG damage induced by hyperglycemia. On the one hand, ART/Met attenuated SMG damage though regulating the PI3K/Akt pathway to inhibit apoptosis and autophagy of SMGs. On the other hand, ART/Met treatment preserved peripheral parasympathetic innervation (AChE and BDNF) in SGs to alleviate diabetes-induced hyposalivation likely through alleviating central SSN damage. T2DM, type 2 diabetes mellitus.

In conclusion, this study has shown that combining ART (first-line antimalarial drug) and Met (frontline drug for T2DM treatment), two relatively non-toxic drugs, exhibited synergistic effects in improvement of xerostomia of diabetic rats induced by HFD/STZ (Figure 9). Both central SSN and SMG damage mediated by hyperglycemia contributed to SGs’ dysfunction. Consequently, our data demonstrated that ART/Met treatment attenuated SMG damage though regulating the PI3K/Akt pathway to inhibit apoptosis and autophagy of SMGs in T2DM rats. Moreover, ART/Met treatment preserved parasympathetic innervation (AChE and BDNF) in SGs to alleviating diabetes-induced hyposalivation likely through rescuing central SSN damage. Taken together, our results suggested that ART in combination with Met treatment might be a viable strategy to ameliorate the detrimental effect of hyperglycemia on SGs’ function, probably via mechanisms mentioned above (Figure 9). However, further in-depth studies and experimental evidence are required to validate and expand the results in the present. In short, the findings of this study may provide a novel insight and theoretical basis for future treating diabetes-induced xerostomia in the clinic.

## Data Availability

The original contributions presented in the study are included in the article/Supplementary Material, further inquiries can be directed to the corresponding author.

## Abbreviations

AB-PAS, Alcian Blue Periodic acid Schiff; AChE, Acetylcholinesterase; ANOVA, Analysis of variance; AQP5, Aquaporin5; ART, Artesunate; AUC, Area under the curve; Bax, Bcl-2-associated X protein; Bcl-2, B-cell lymphoma 2; BDNF, Brain-derived neurotrophic factor; FBG, Fasting blood glucose; HDL, High-density lipoprotein; H&E, Hematoxylin and eosin; LDL, Low-density lipoprotein; MET, Metformin; OGTT, Oral glucose tolerance; PI3K/AKT, Phosphatidylinositol 3ʹ-kinase/protein kinase B; SG, Salivary gland; SMGs, Submandibular glands; SSN, Superior salivatory nucleus; STZ, Streptozotocin; SQSTM1/p62, Sequestosome 1; T2DM, Type 2 Diabetes mellitus; TC, Total cholesterol; TG, Triglycerides.
