# Supplementary figures and images for: Artesunate Combined With Metformin Ameliorate on Diabetes-Induced Xerostomia by Mitigating Superior Salivatory Nucleus and Salivary Glands Injury in Type 2 Diabetic Rats via the PI3K/AKT Pathway
Source: Front Pharmacol. 2021 Dec 20;12:774674. doi: 10.3389/fphar.2021.774674 (PMC8722737; doi:10.3389/fphar.2021.774674)

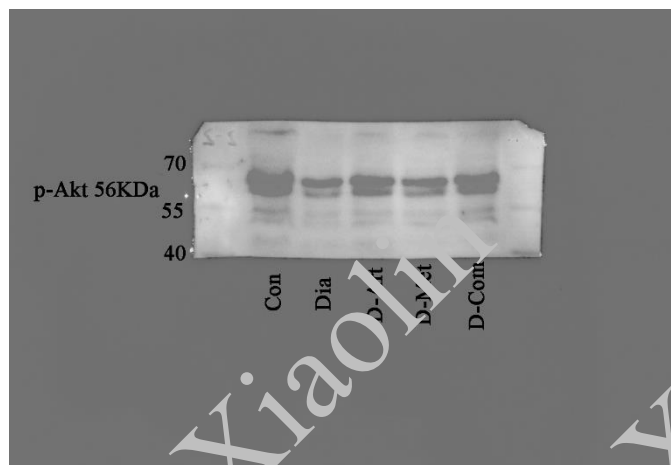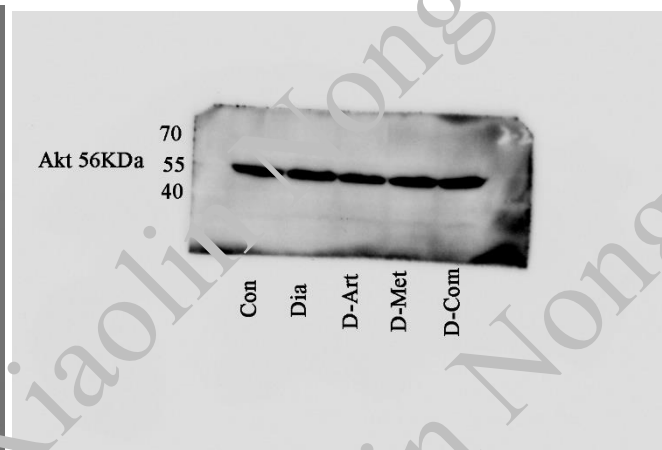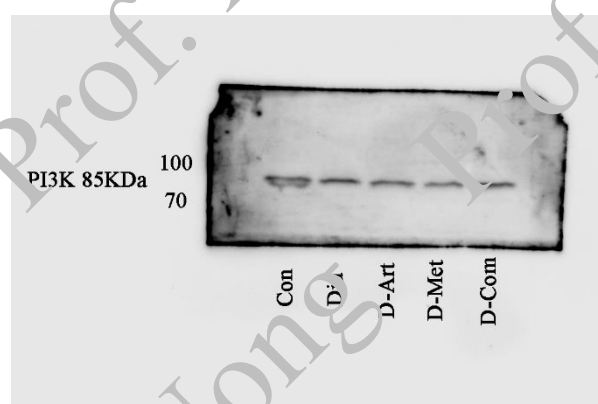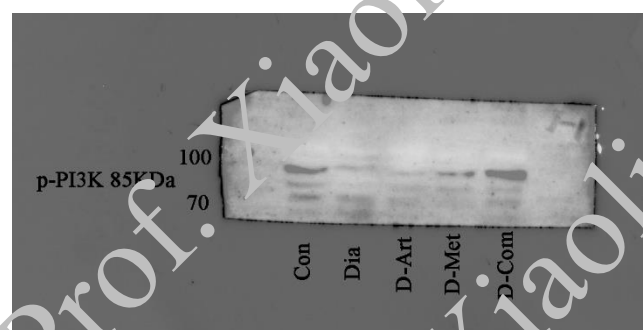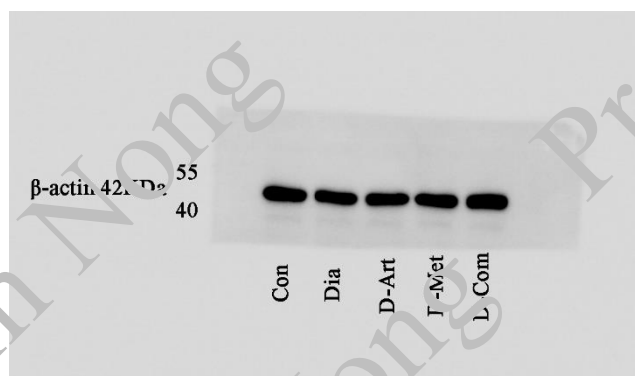

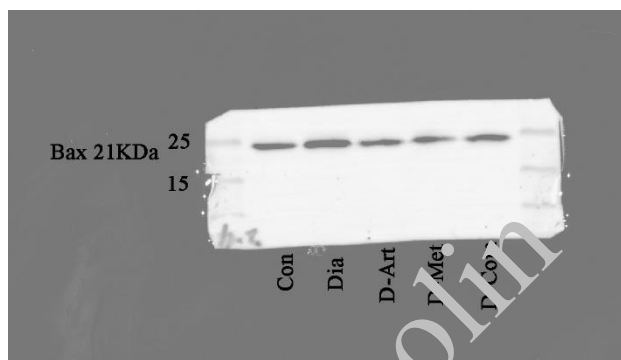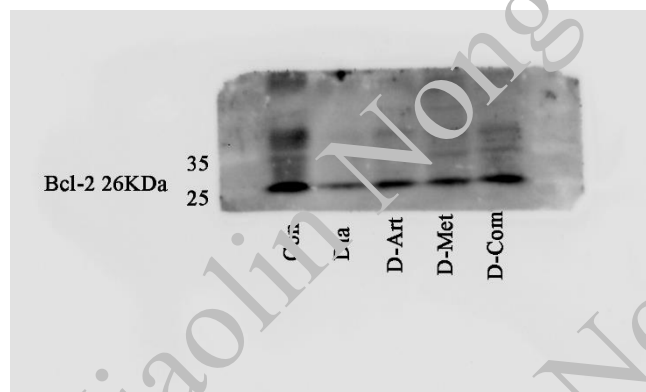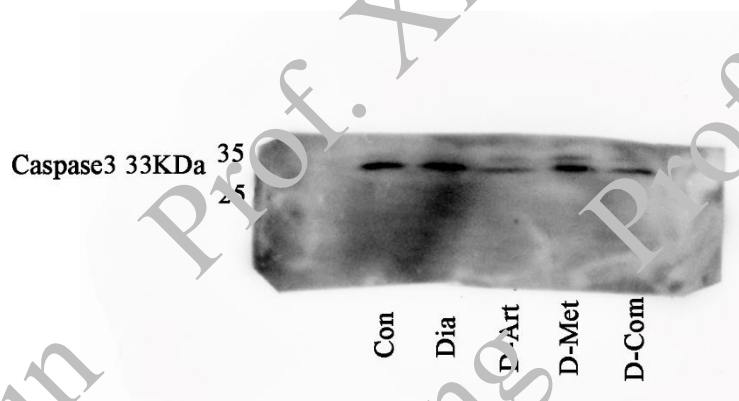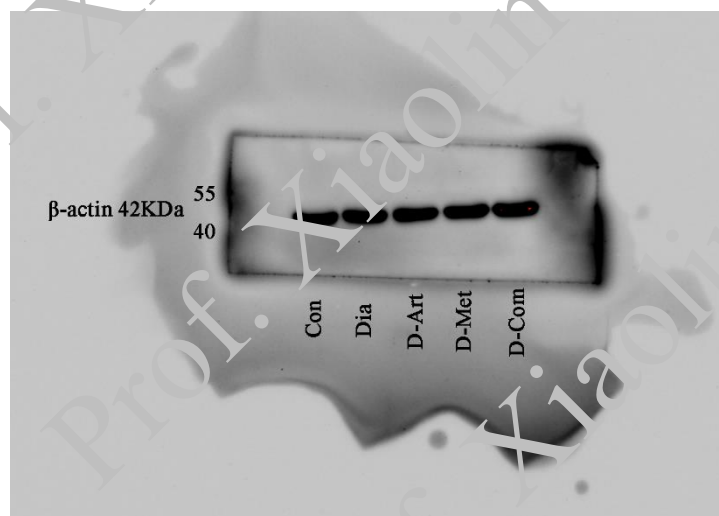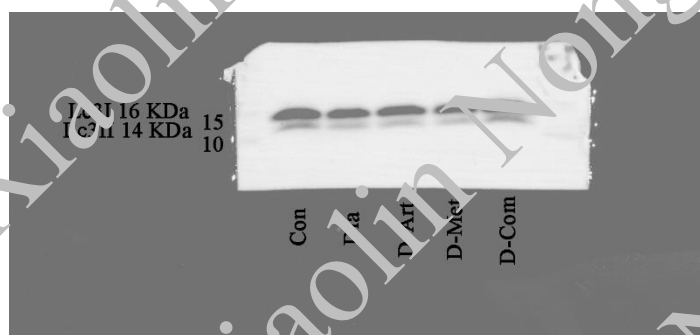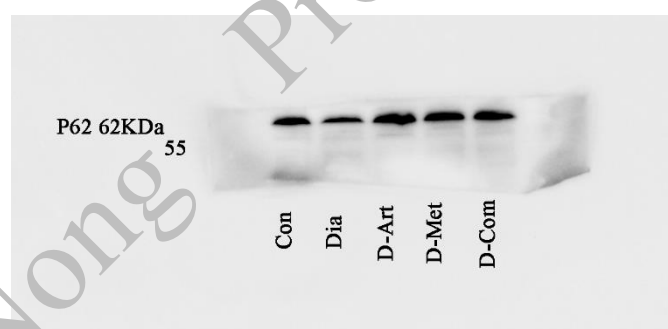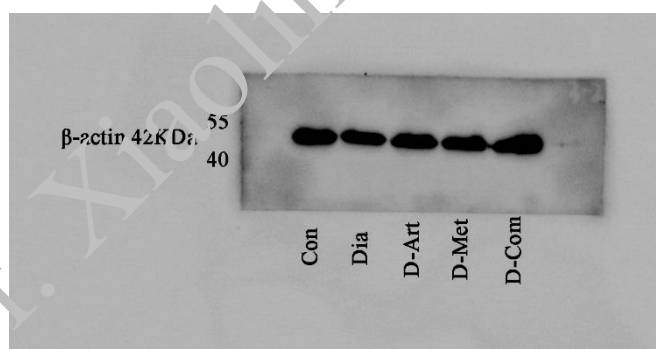

Supplement: Supplementary file 1 [file DataSheet2.PDF]
