# Supplementary material for: Artesunate Combined With Metformin Ameliorate on Diabetes-Induced Xerostomia by Mitigating Superior Salivatory Nucleus and Salivary Glands Injury in Type 2 Diabetic Rats via the PI3K/AKT Pathway
Source: Front Pharmacol. 2021 Dec 20;12:774674. doi: 10.3389/fphar.2021.774674 (PMC8722737; doi:10.3389/fphar.2021.774674)
Supplement: Supplementary file 2 [file DataSheet4.PDF]

AQP5

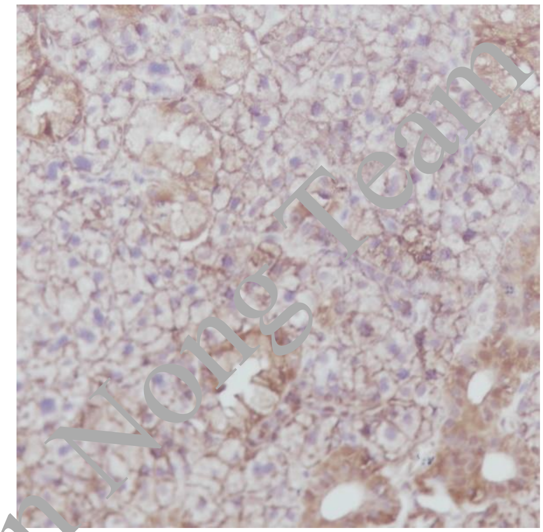

CON

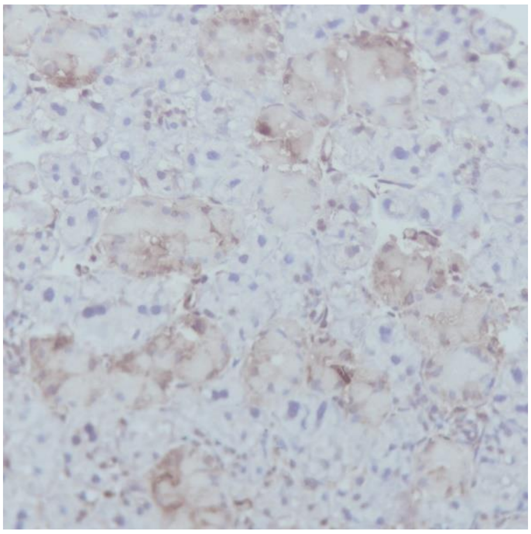

Dia

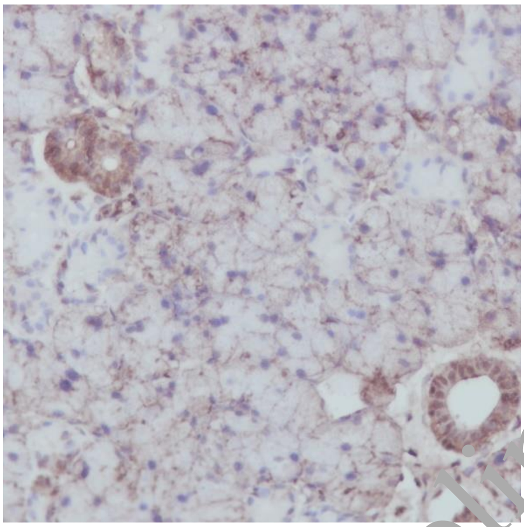

D-ART

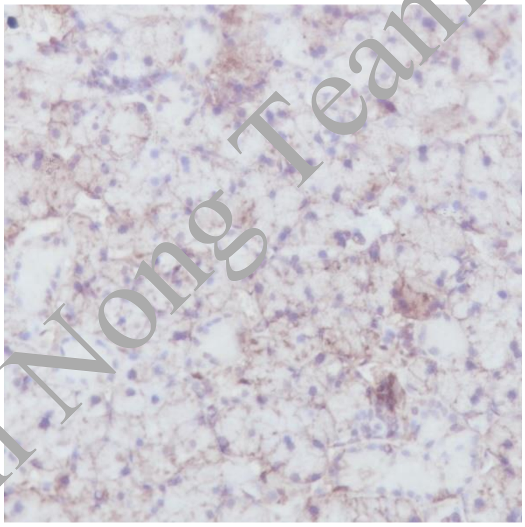

D-Met

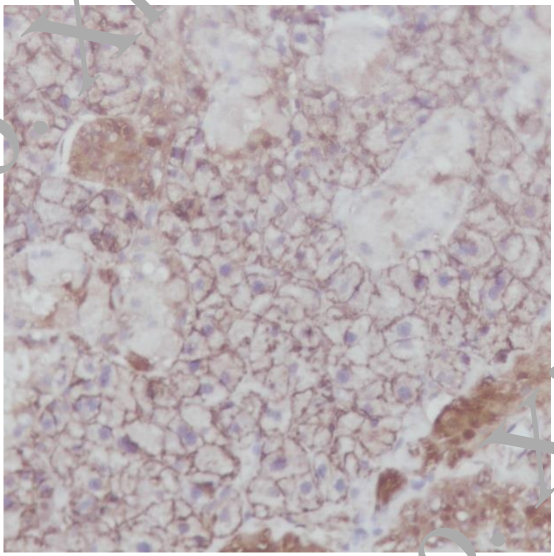

D-Com

Pro. Xiaolin Nong Team

AchE

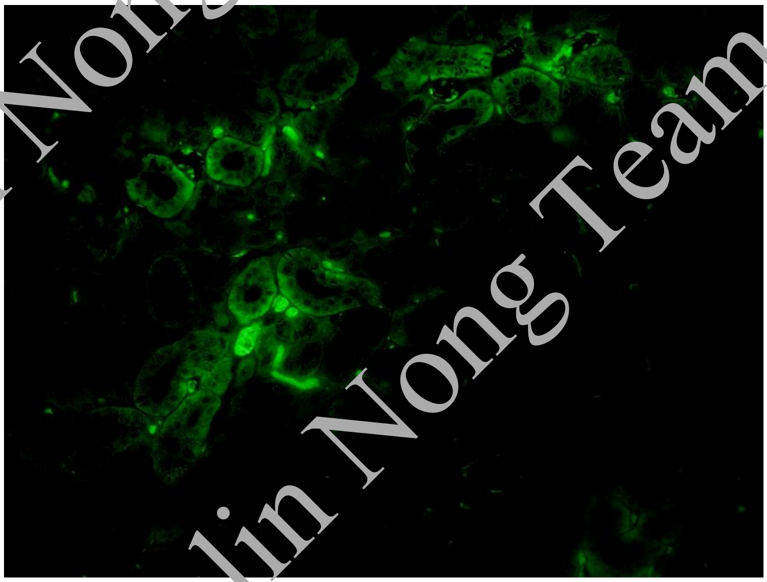

Con-AchE

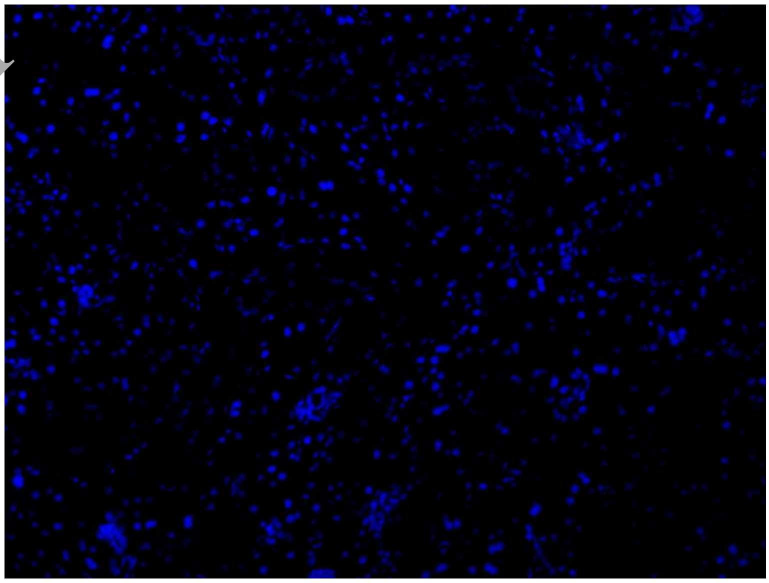

Con-DAPI

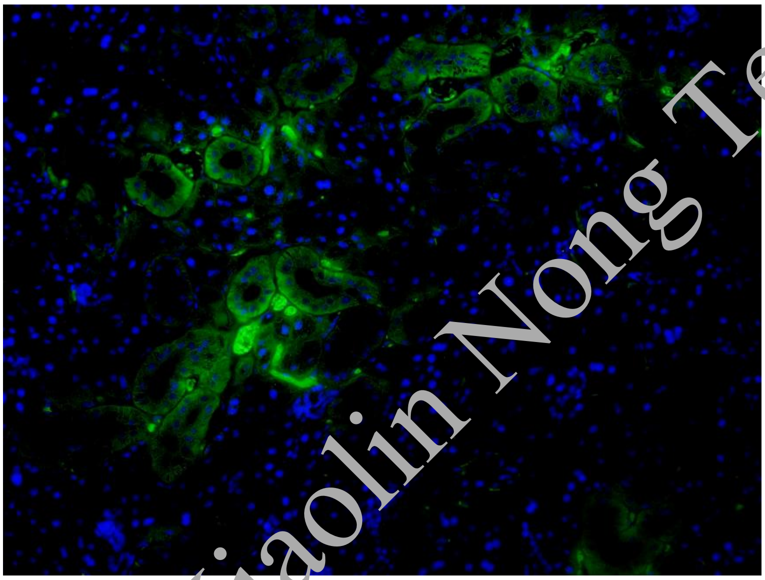

Con-Merge

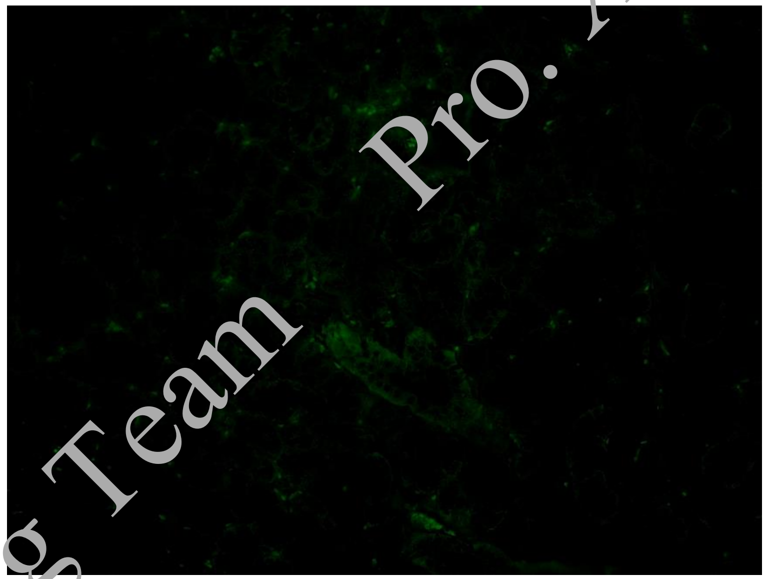

Dia-AchE

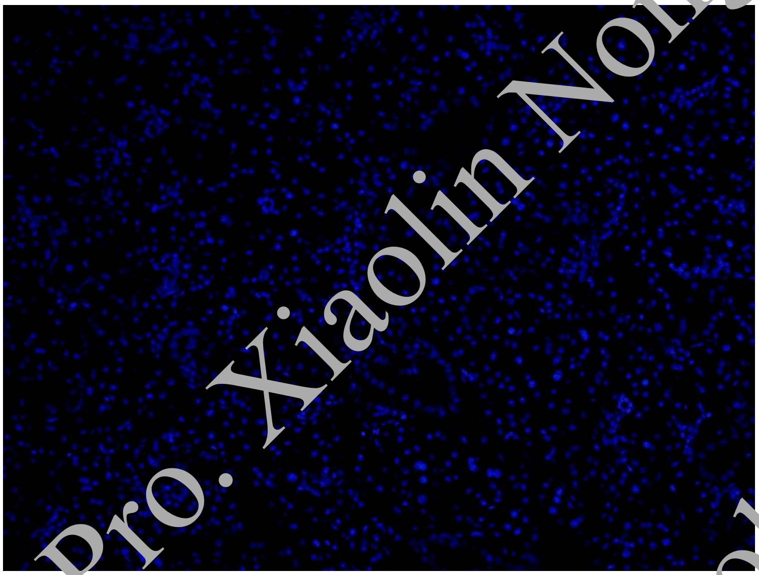

Dia-DAPI

Pro. Xiaolin Nong Team

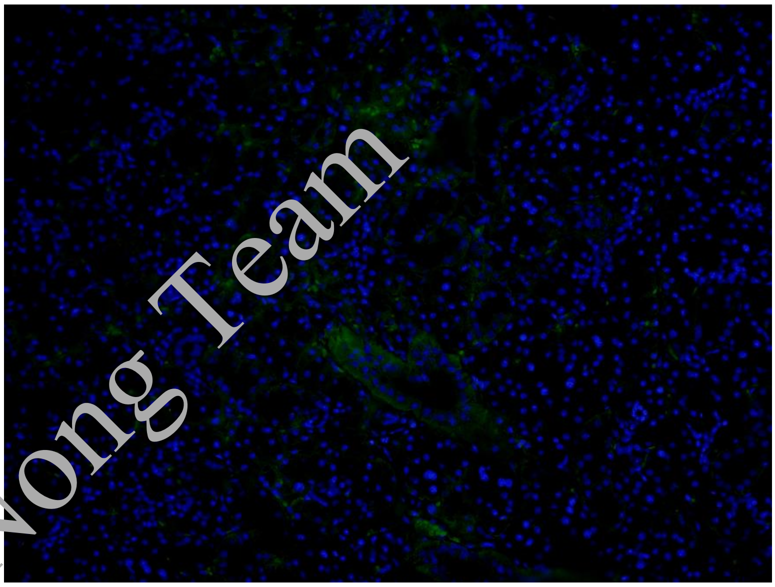
Dia-Merge

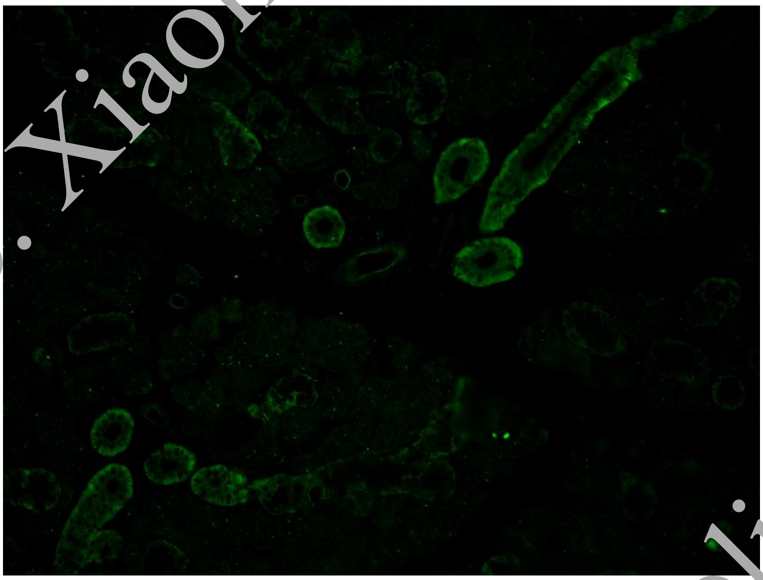
D-Art-AchE

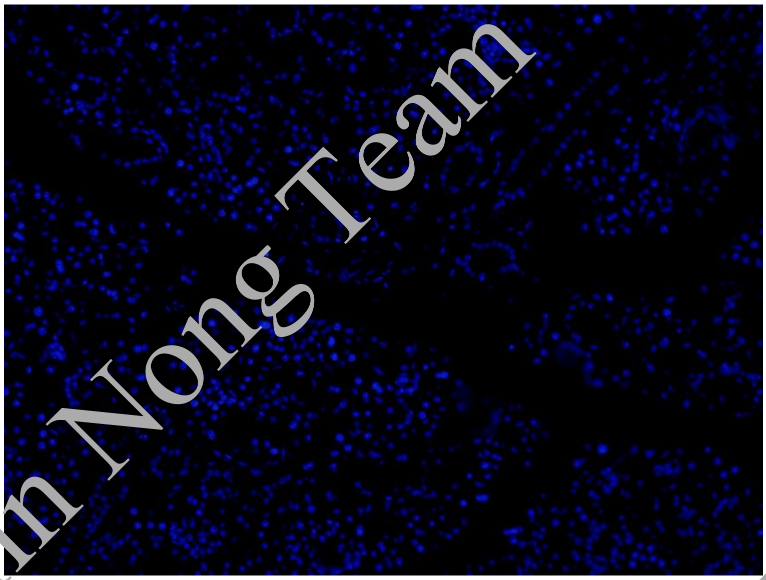
D-Art-DAPI

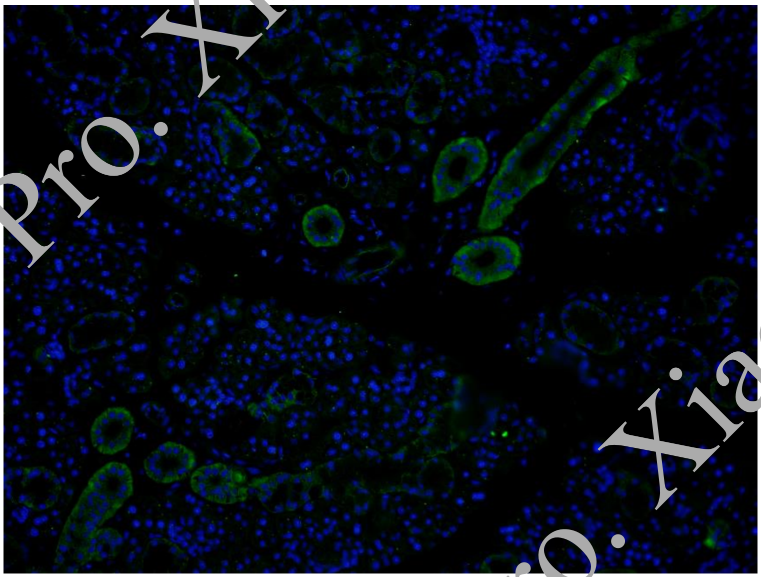
D-Art-Merge

Pro. Xiaolin Nong Team

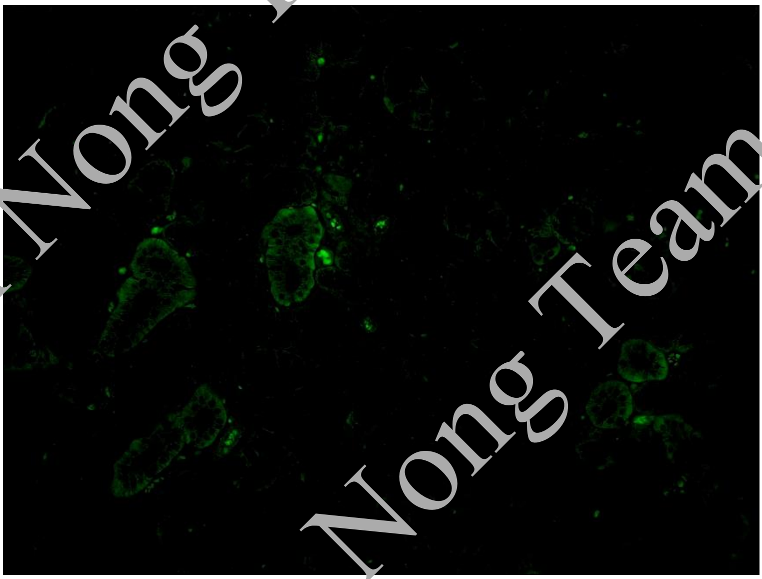

D-Met-AchE

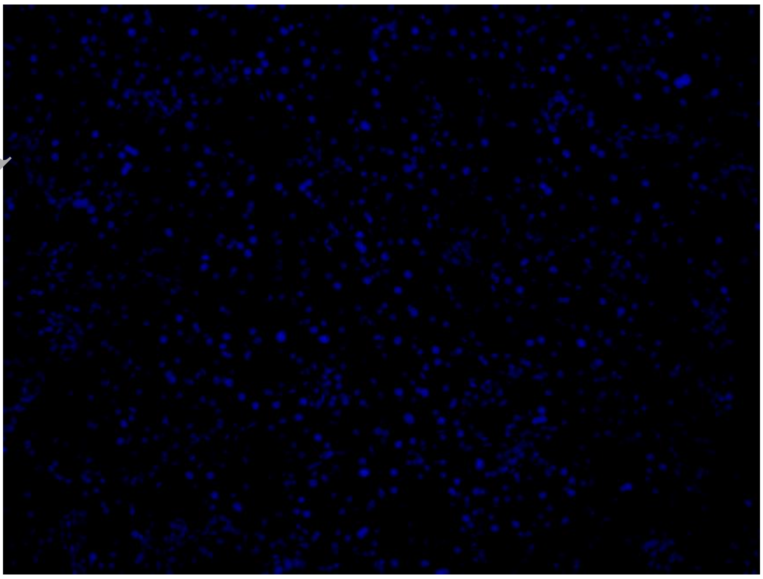

D-Met-DAPI

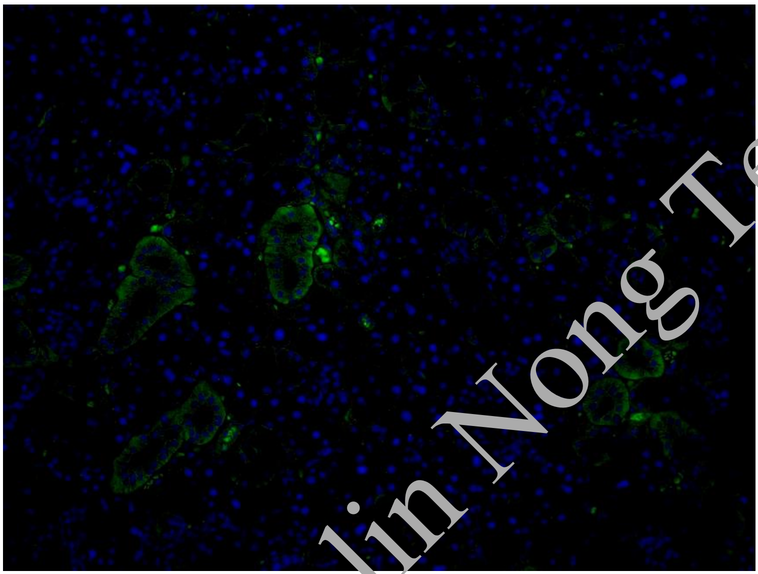

D-Met-Merge

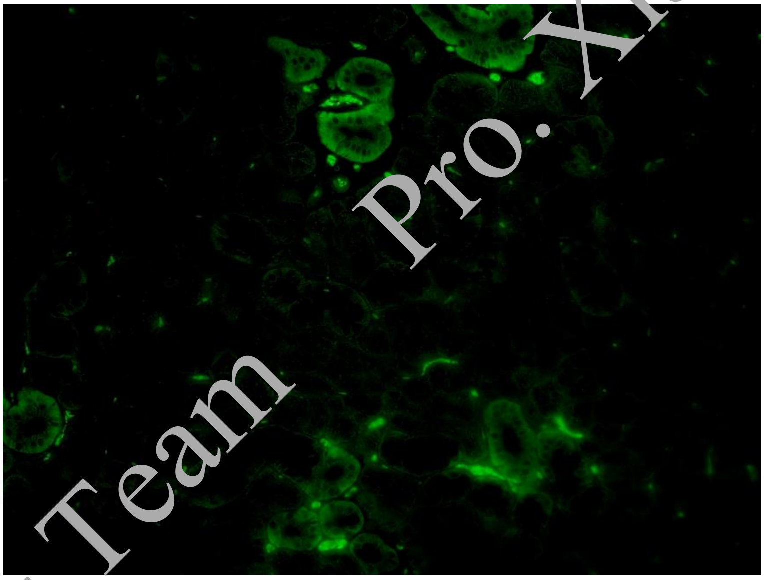

D-Com-AchE

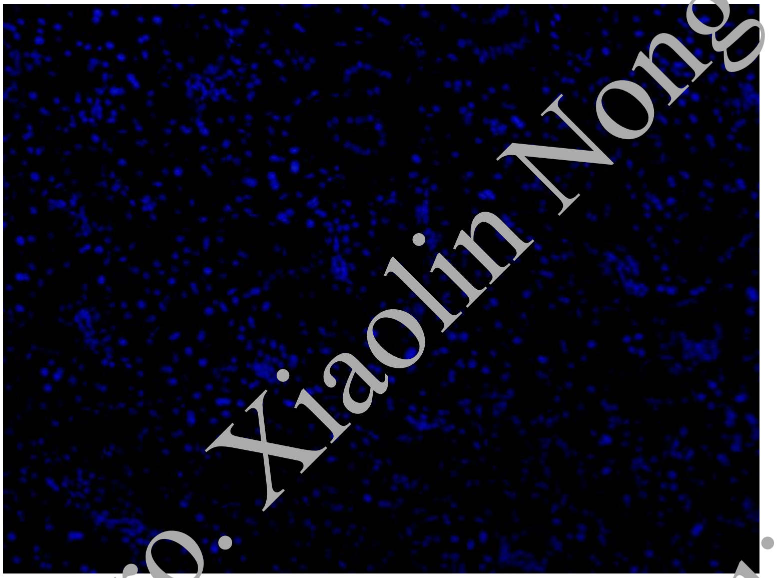

D-Com-DAPI

Pro. Xiaolin Nong Team

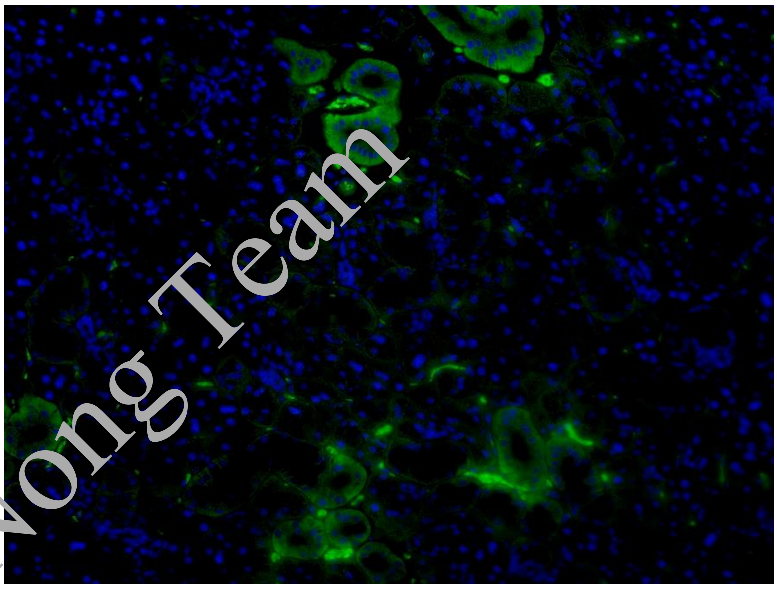

D-Com-Merge

Pro. Xiaolin Nong Team

TUNEL staining

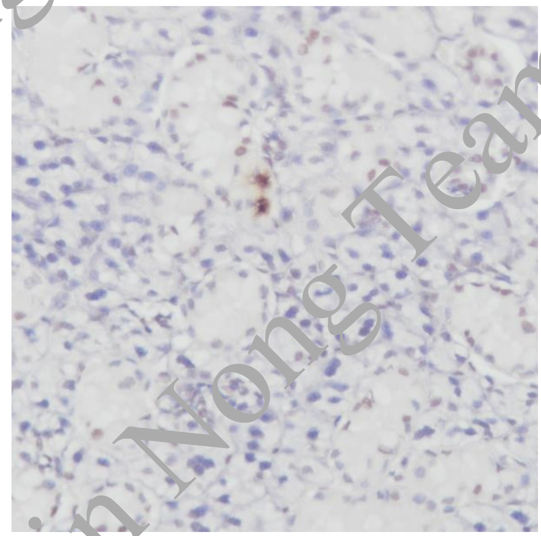

Con

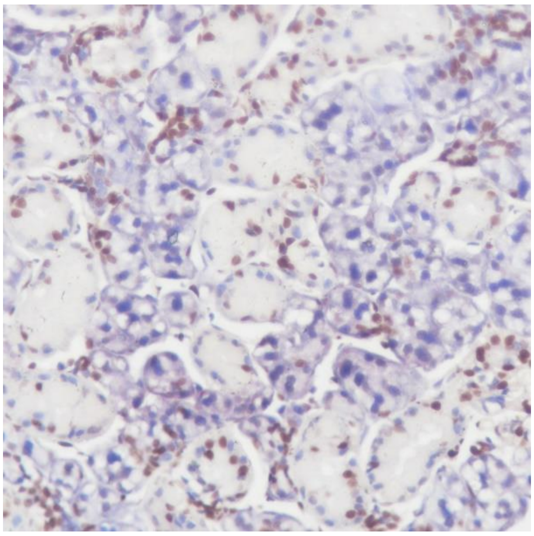

Dia

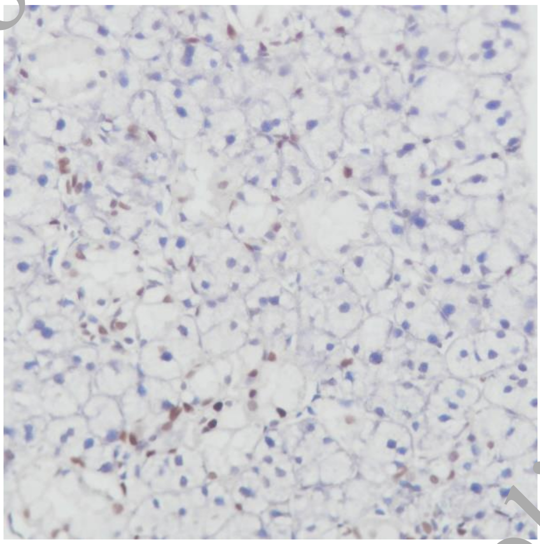

D-ART

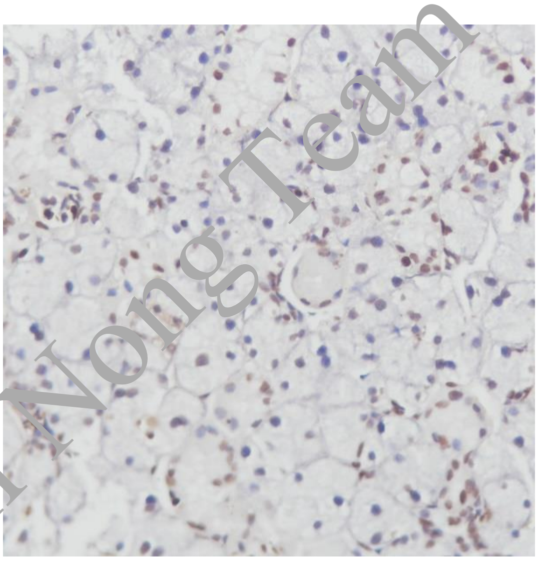

D-Met

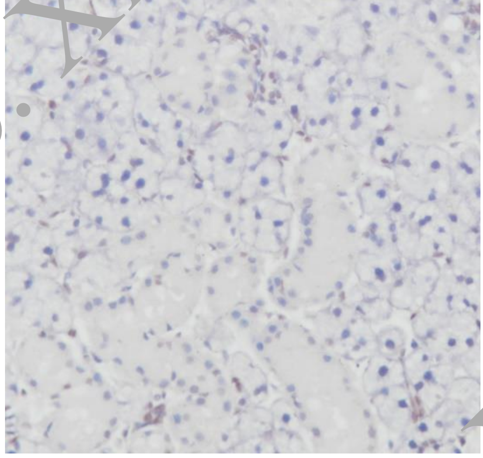

D-Com

Pro. Xiaolin Nong Team
