# Supplementary material for: Artesunate Combined With Metformin Ameliorate on Diabetes-Induced Xerostomia by Mitigating Superior Salivatory Nucleus and Salivary Glands Injury in Type 2 Diabetic Rats via the PI3K/AKT Pathway
Source: Front Pharmacol. 2021 Dec 20;12:774674. doi: 10.3389/fphar.2021.774674 (PMC8722737; doi:10.3389/fphar.2021.774674)
Supplement: Supplementary file 3 [file DataSheet3.PDF]

Pancreas（AB-PAS）

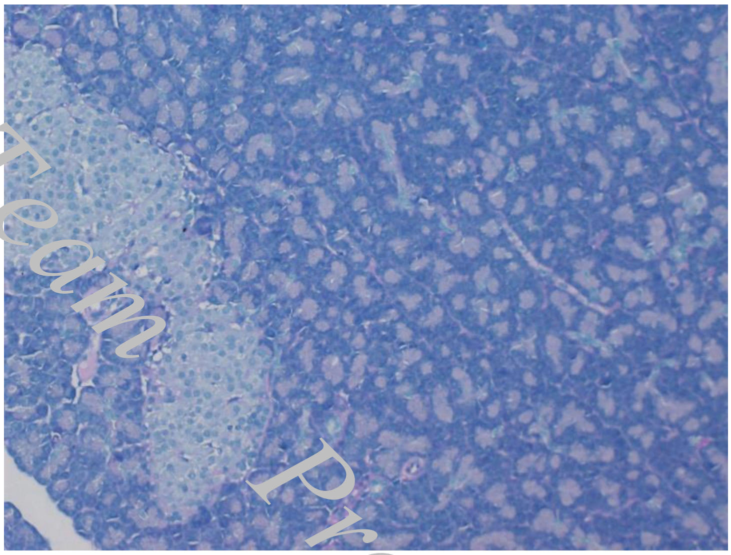

20×-CON

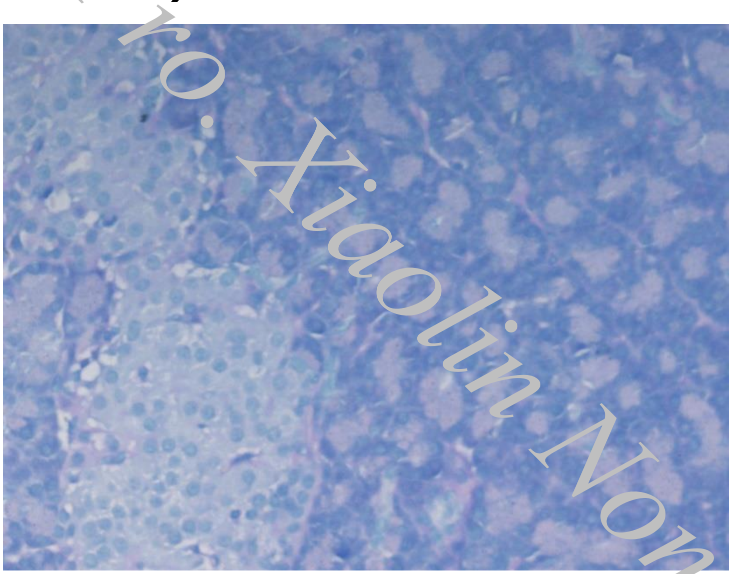

40 × -CON

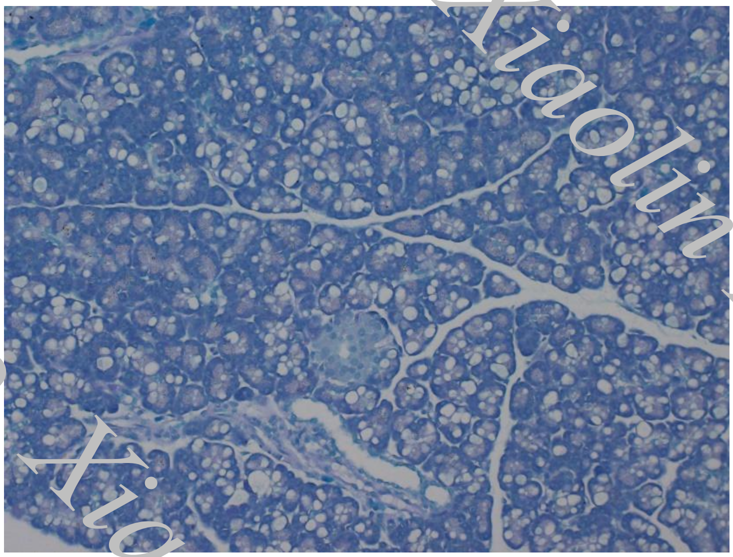

20×-Dia

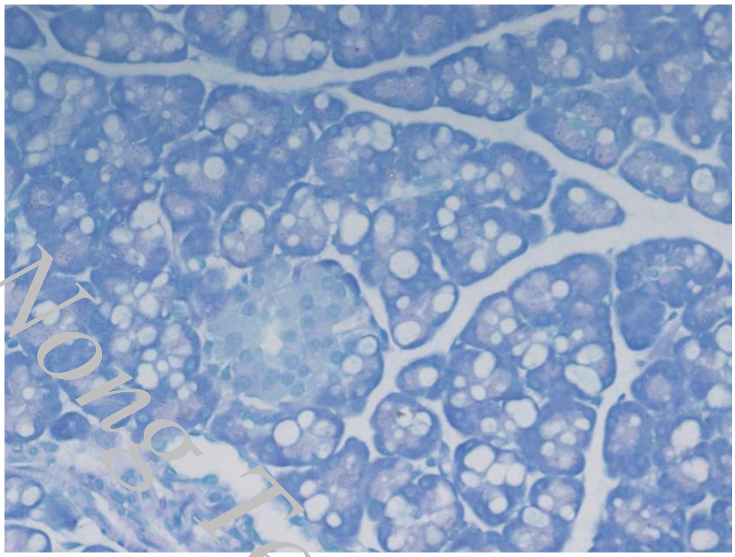

40×-Dia

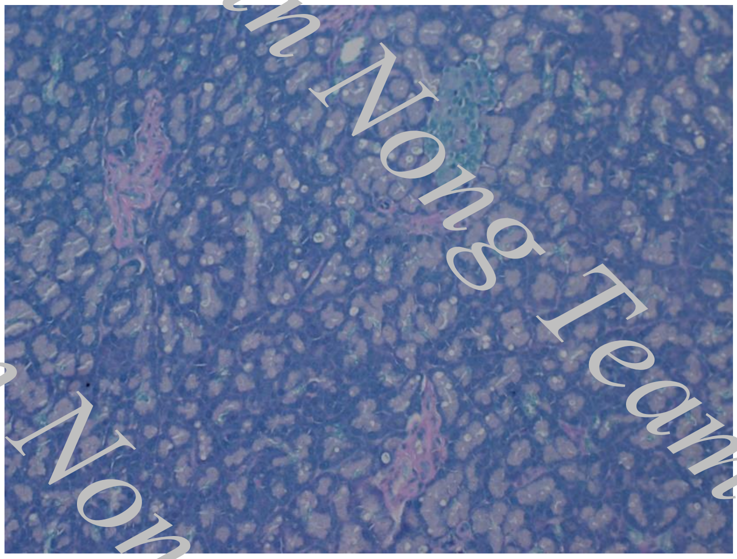

20×-D-Art

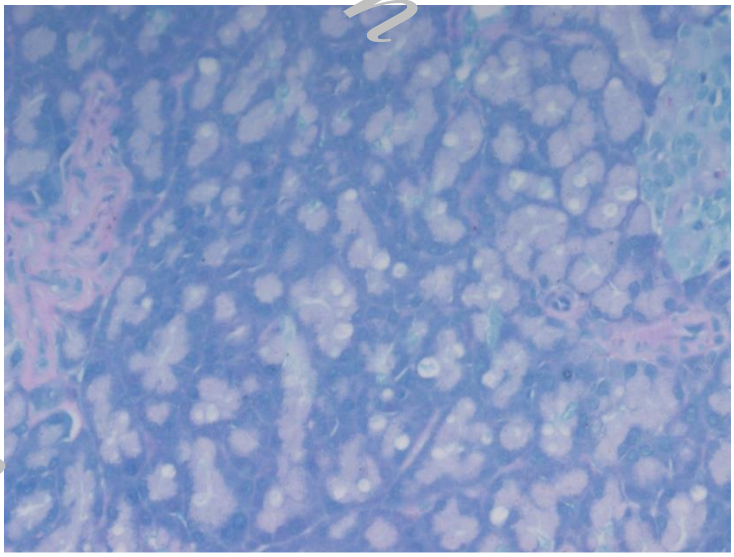

40×-D-Art

Pro. Xiaolin Nong Team

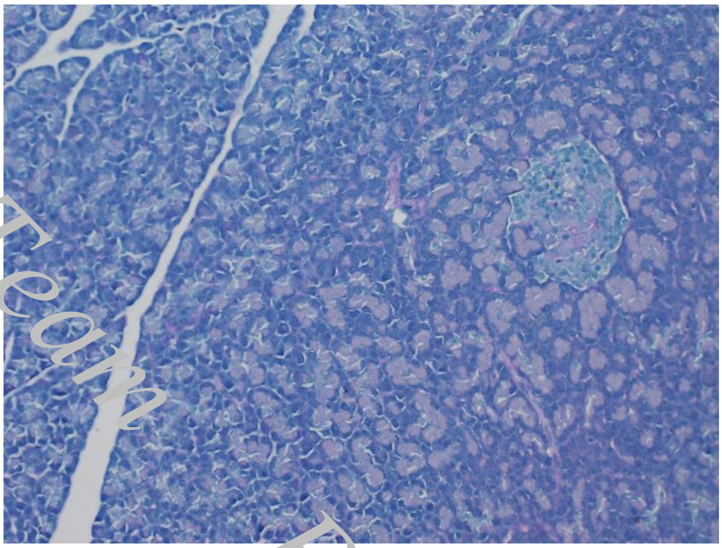

20×-D-Met

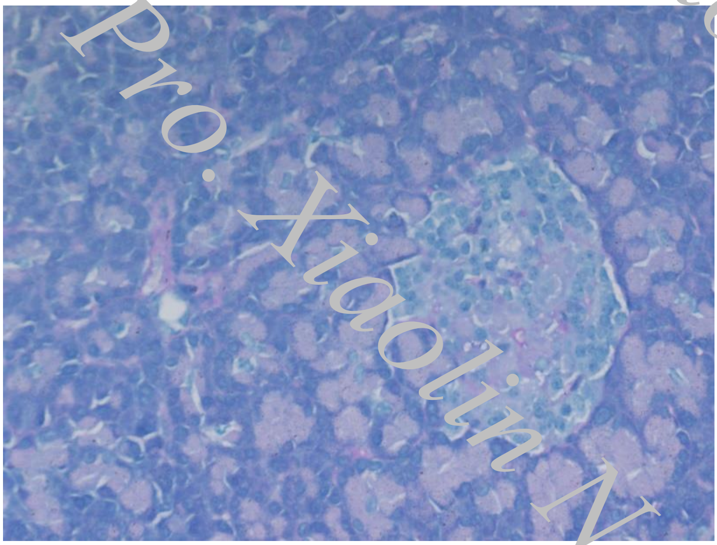

40×-D-Met

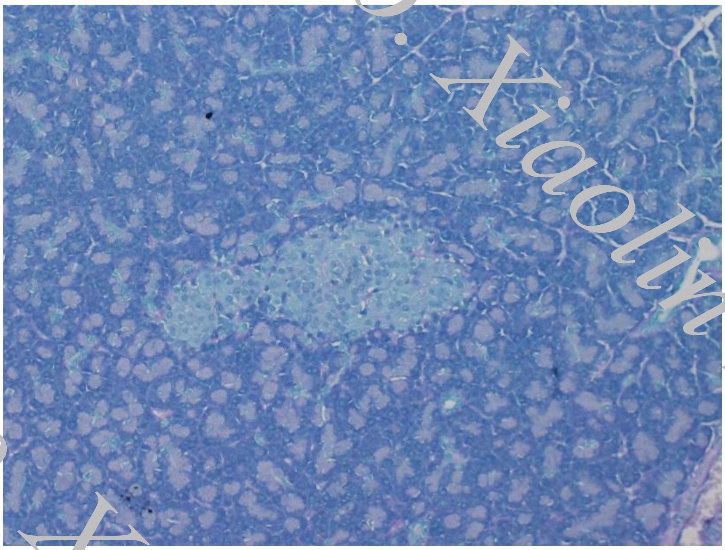

20×-D-Com

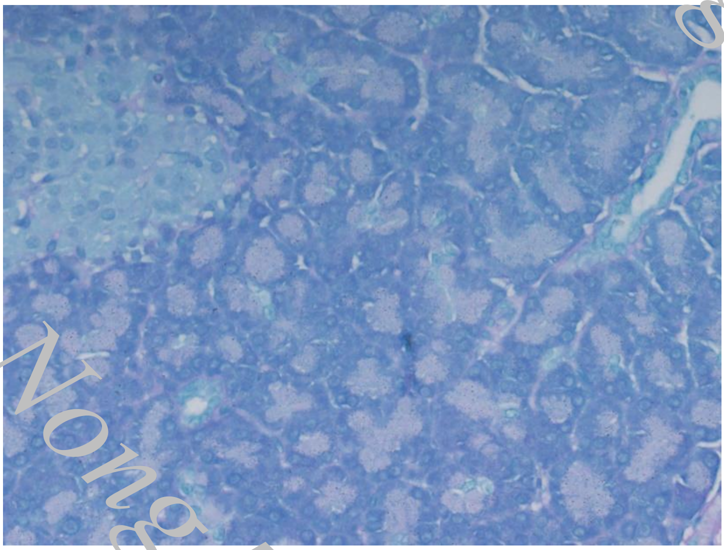

40×-D-Com

Pro. Xiaolin Nong Team

Pancreas (HE)

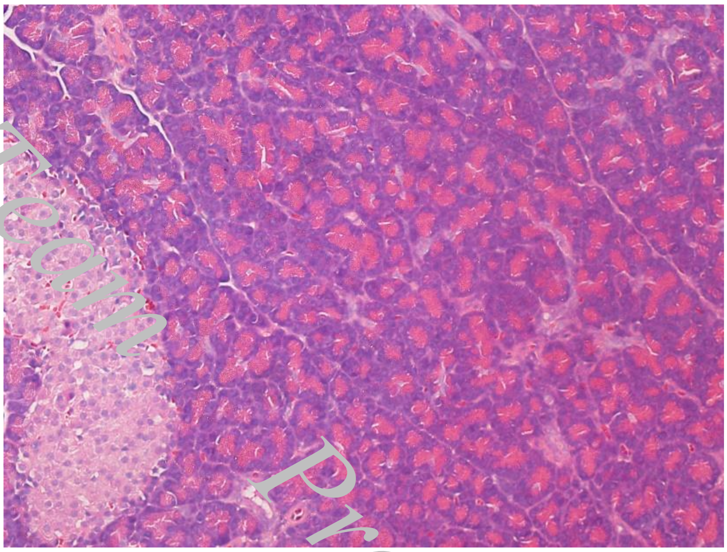

20×-Con

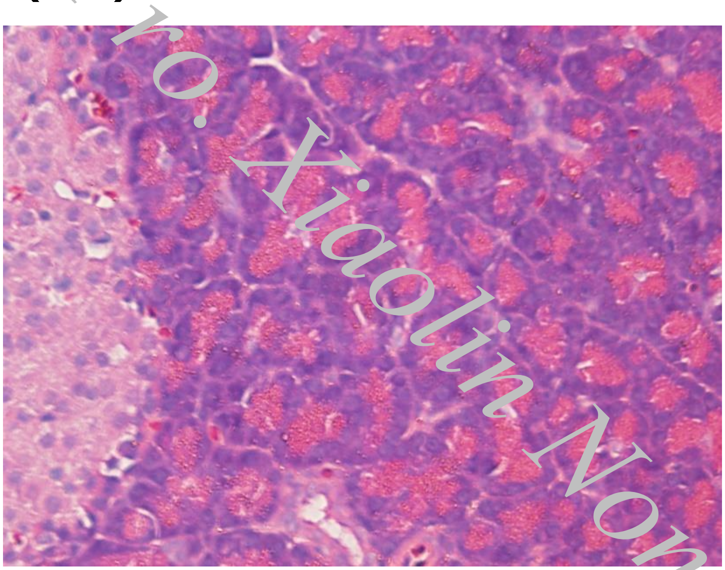

40×-Con

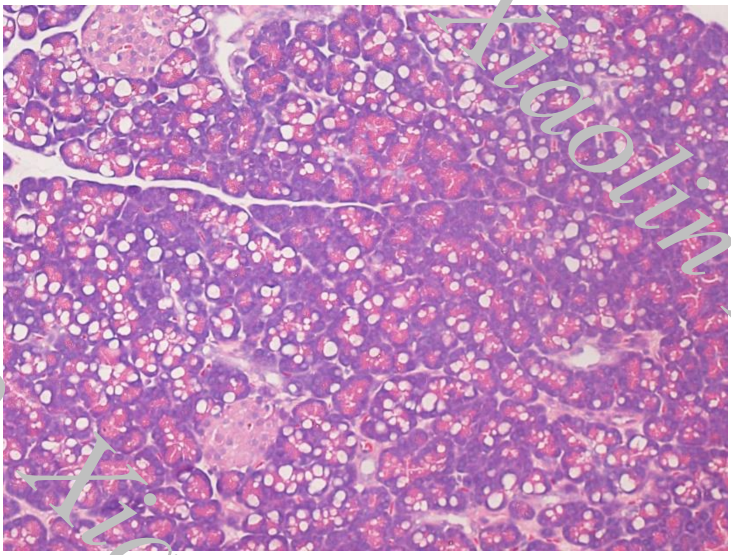

20×-Dia

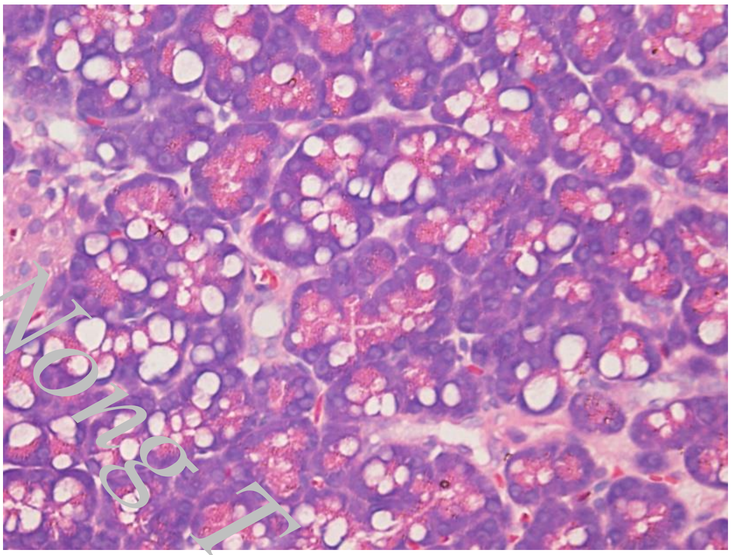

40×-Dia

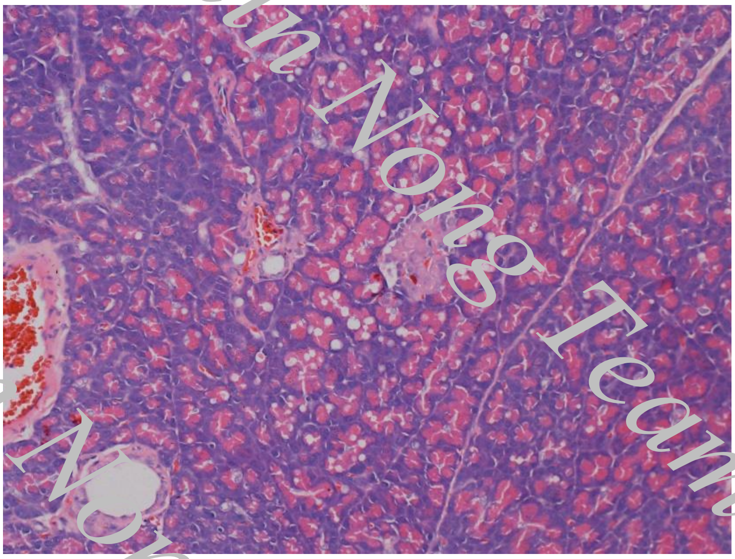

20×-D-Art

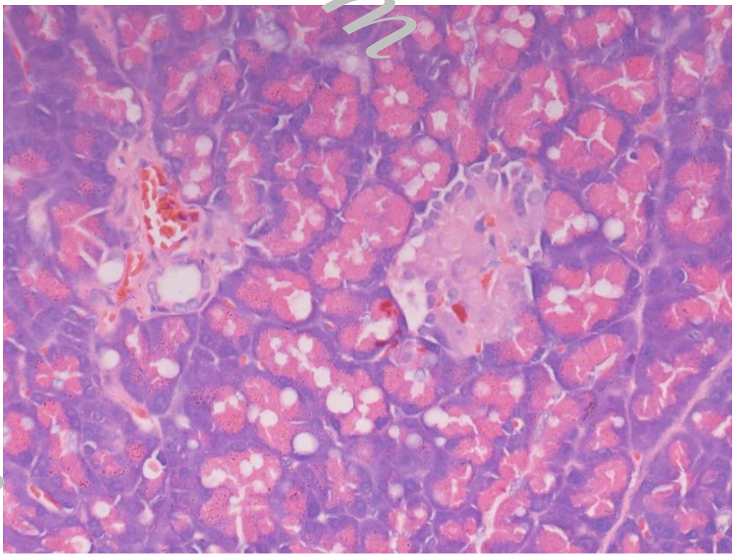

40×-D-Art

Pro. Xiaolin Nong Team

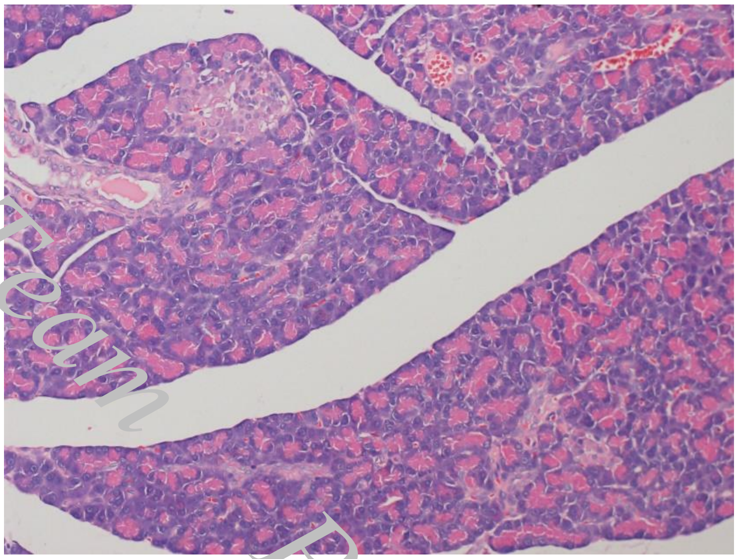

20×-D-Met

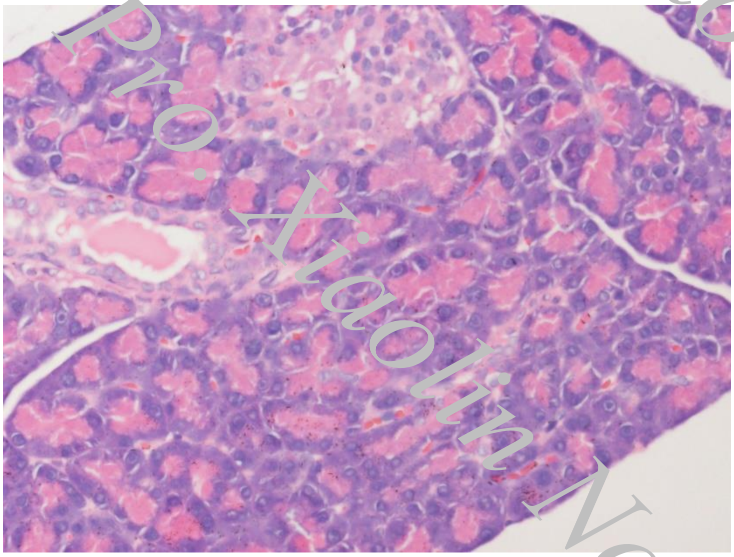

40×-D-Met

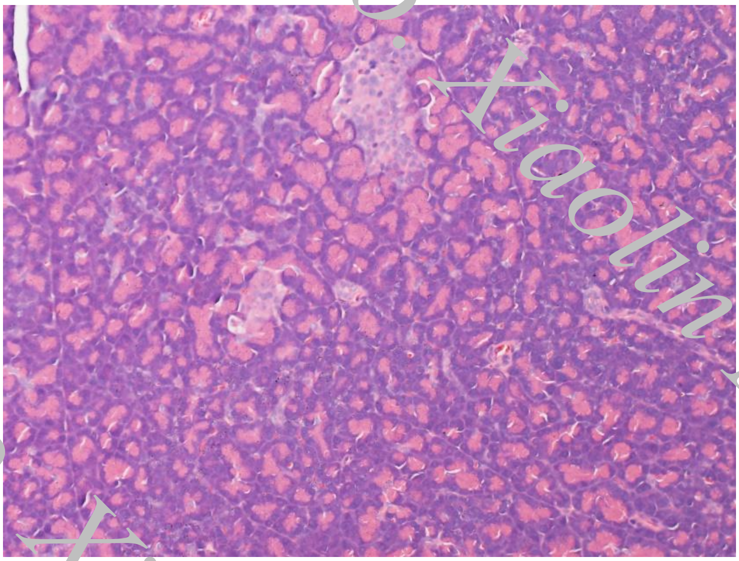

20×-D-Com

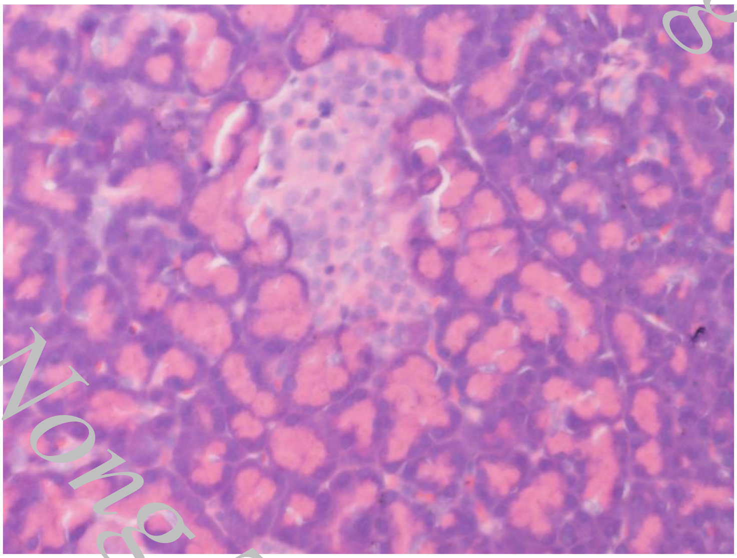

40×-D-Com

Pro. Xiaolin Nong Team

SMG（AB-PAS）

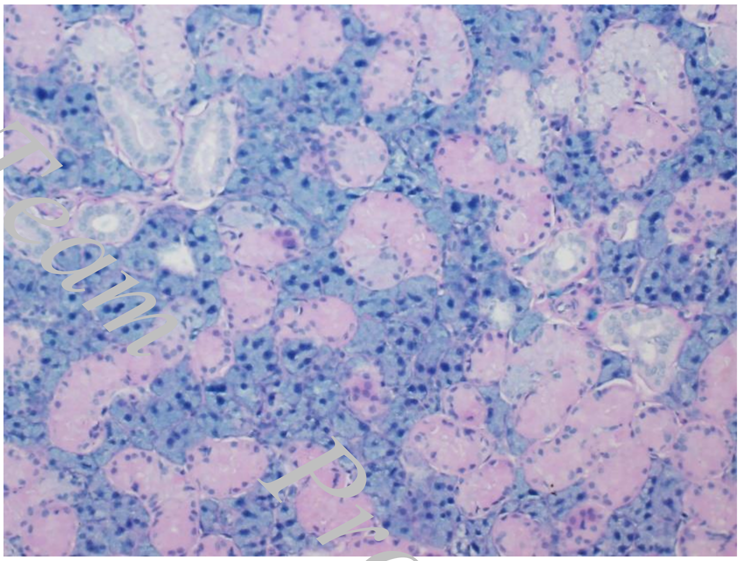

20×-Con

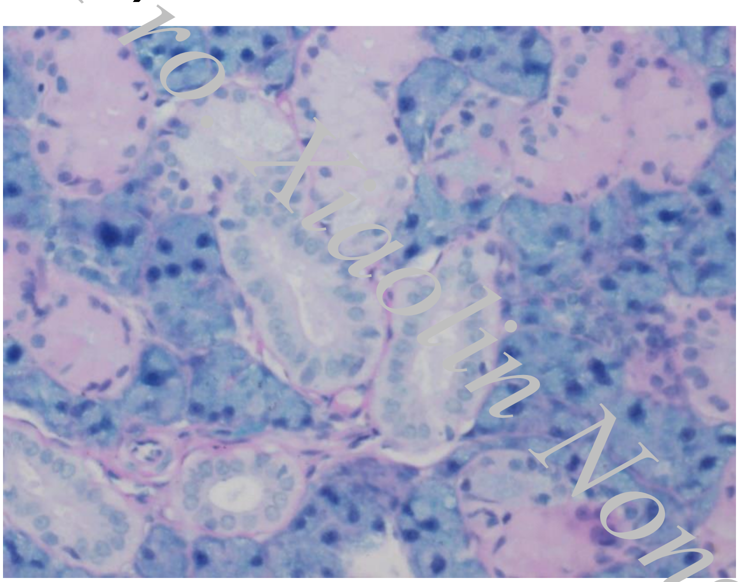

40×-Con

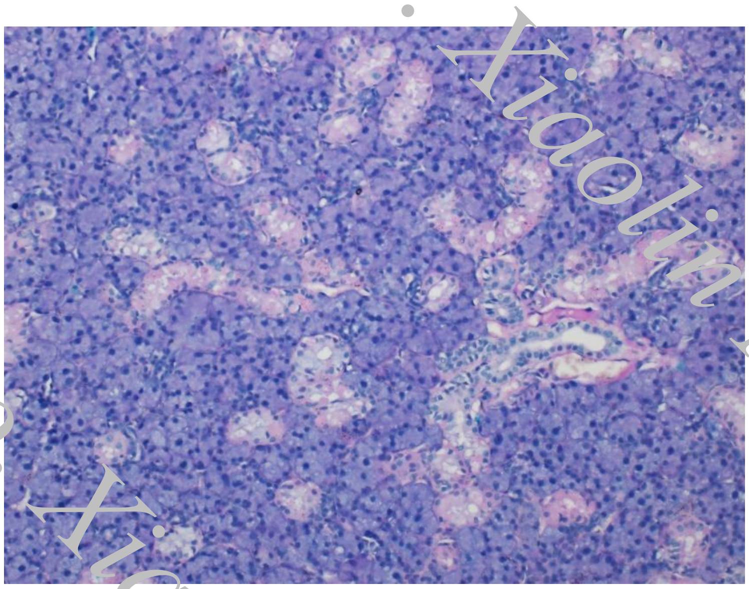

20×-Dia

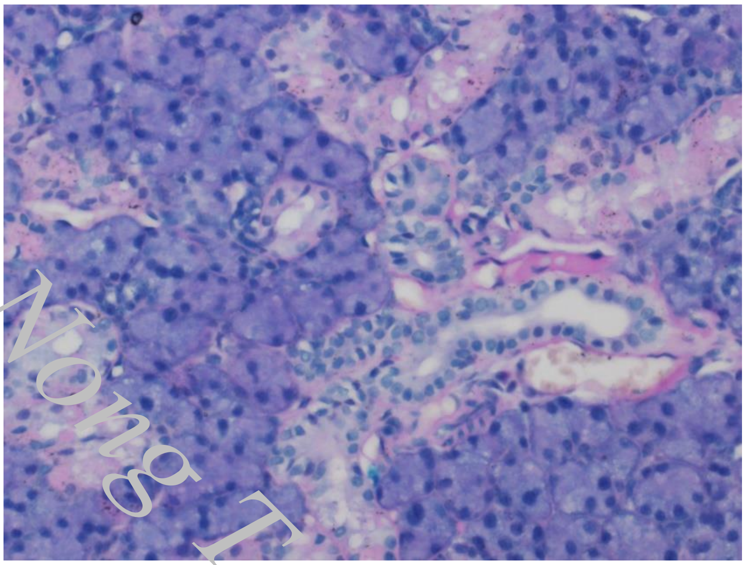

40×-Dia

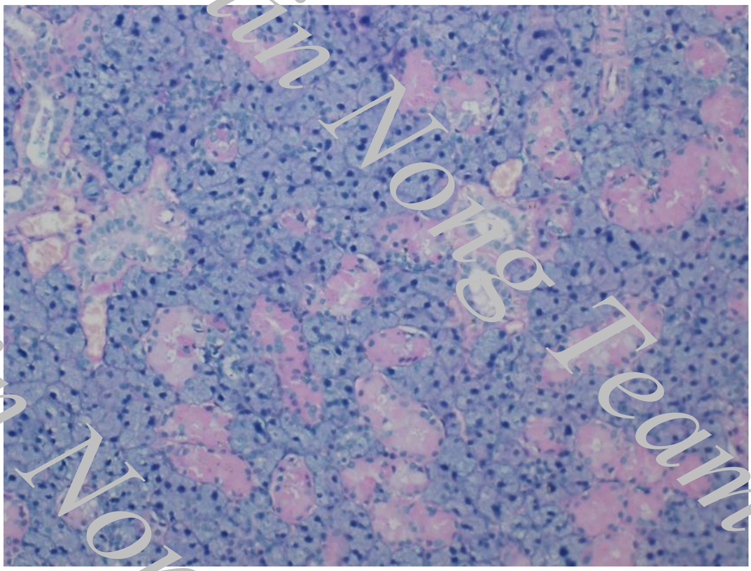

20×-D-Art

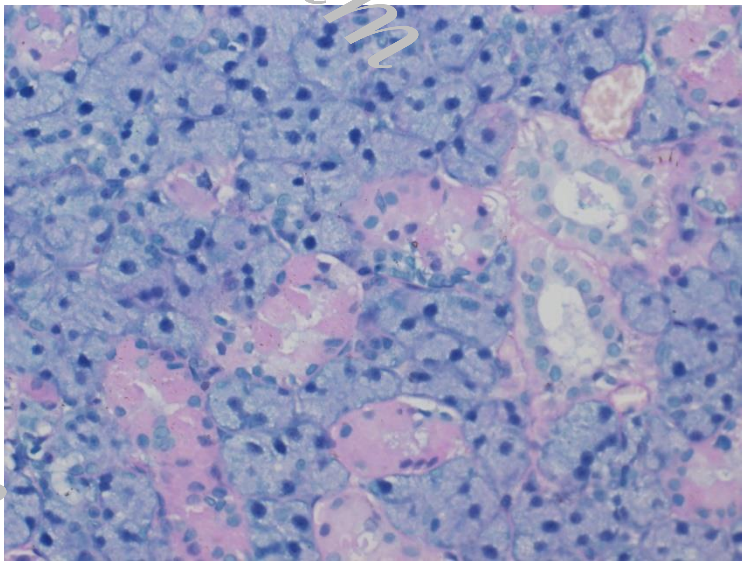

40×-D-Art

Pro. Xiaolin Nong Team

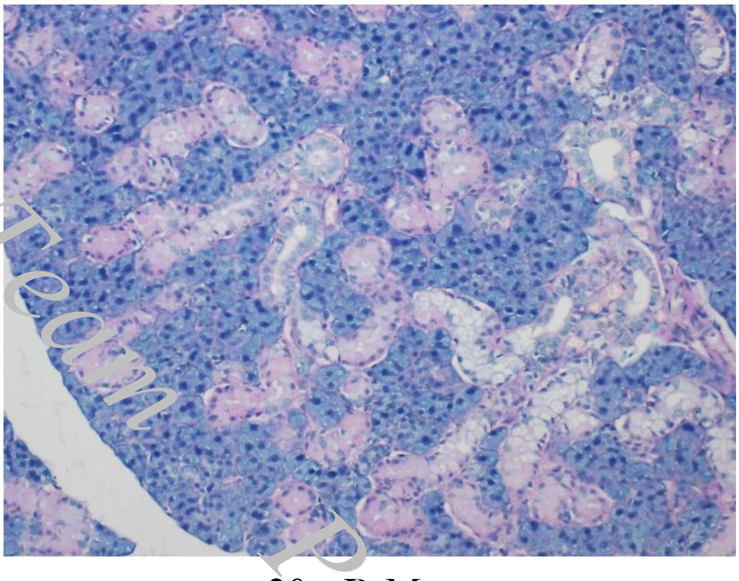

20×-D-Met

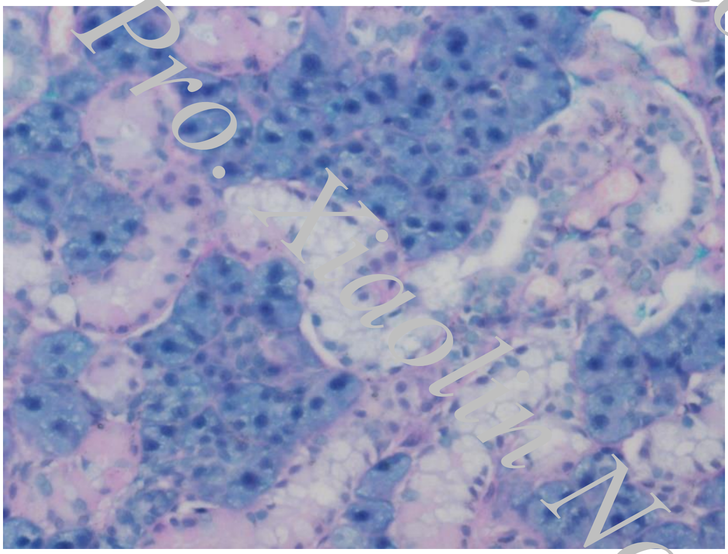

40×-D-Met

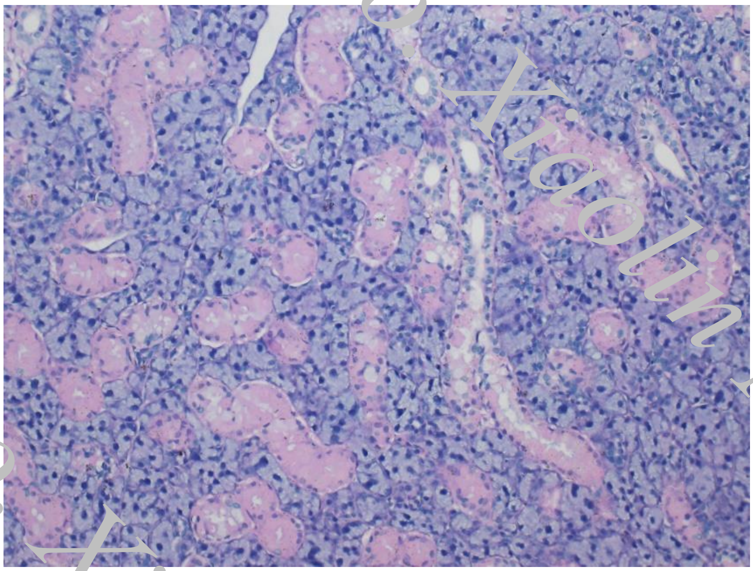

20×-D-Com

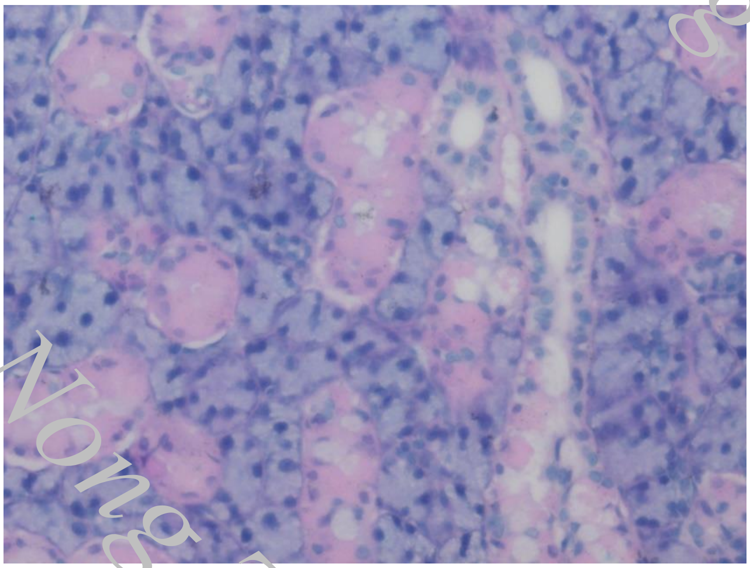

40×-D-Com

Pro. Xiaolin Nong Team

SMG（HE）

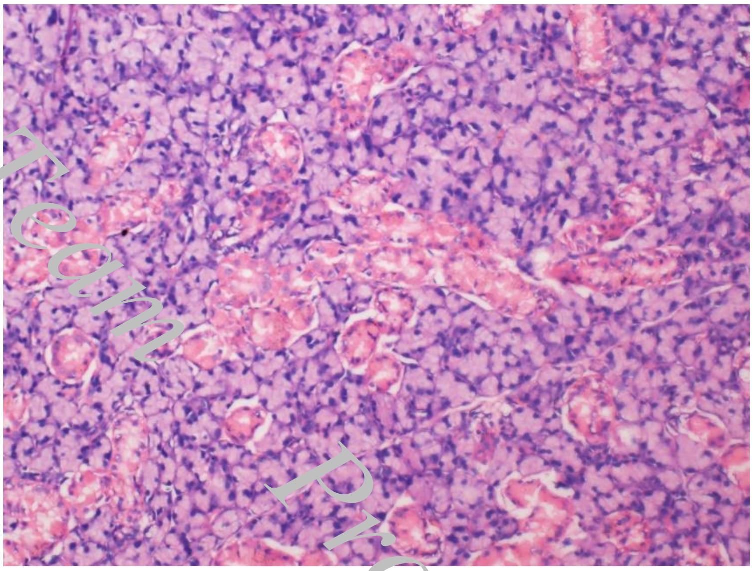

20×-Con

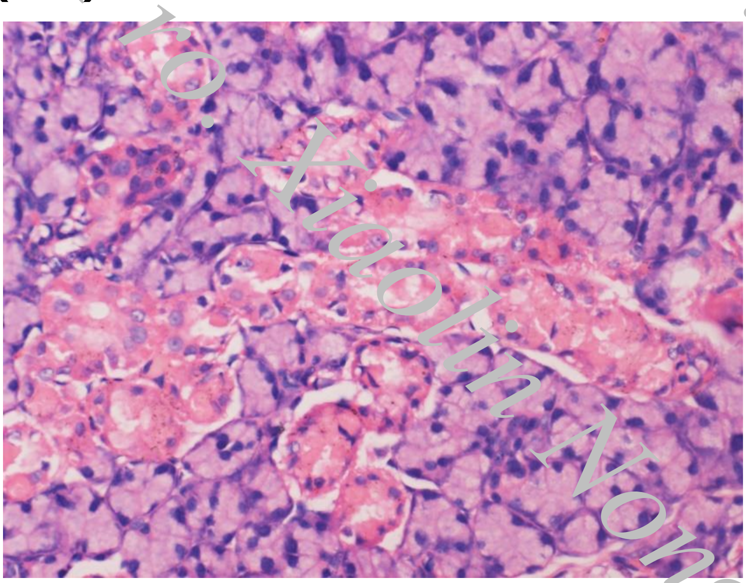

40×-Con

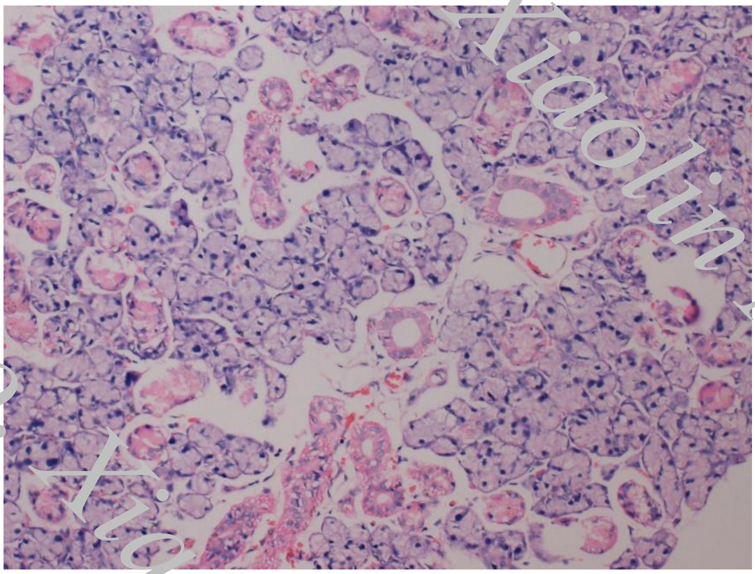

20×-Dia

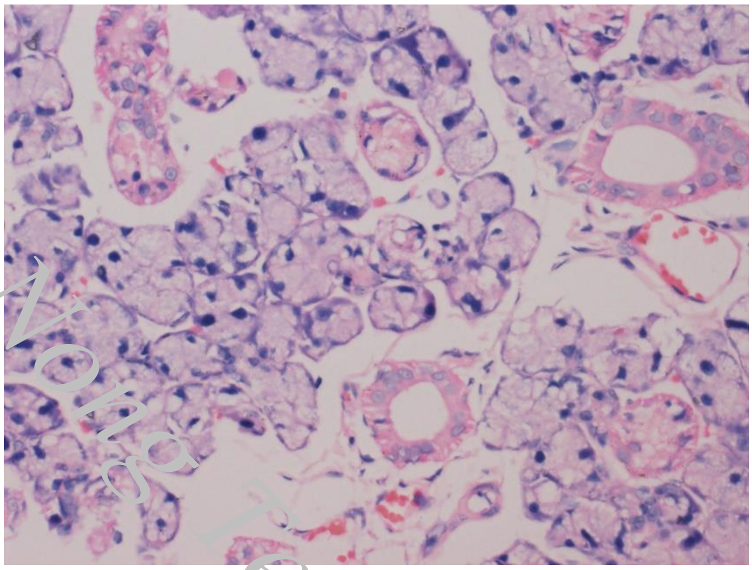

40×-Dia

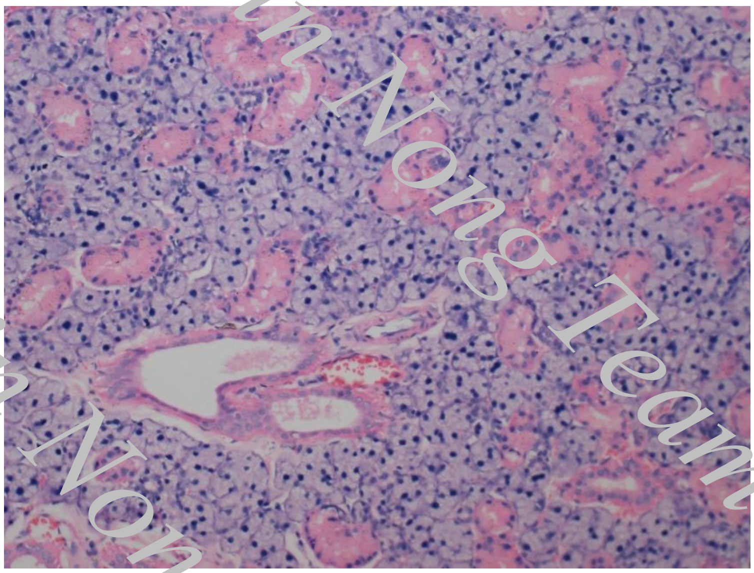

20×-D-Art

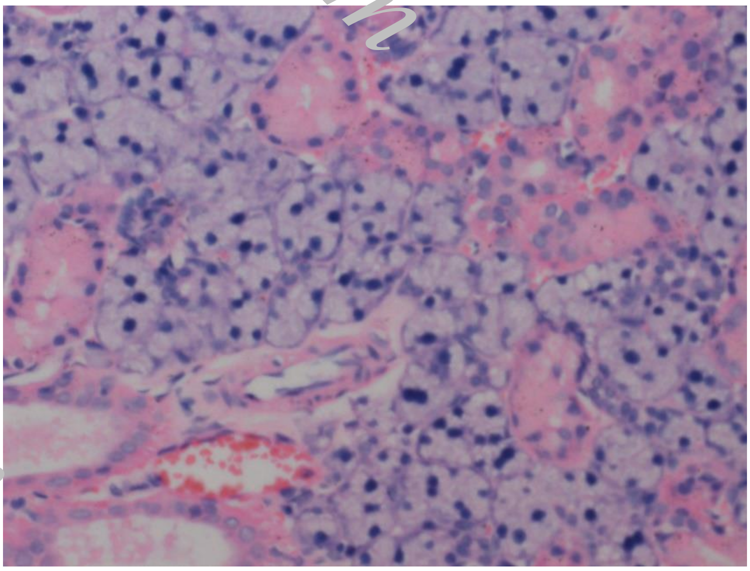

40×-D-Art

Pro. Xiaolin Nong Team

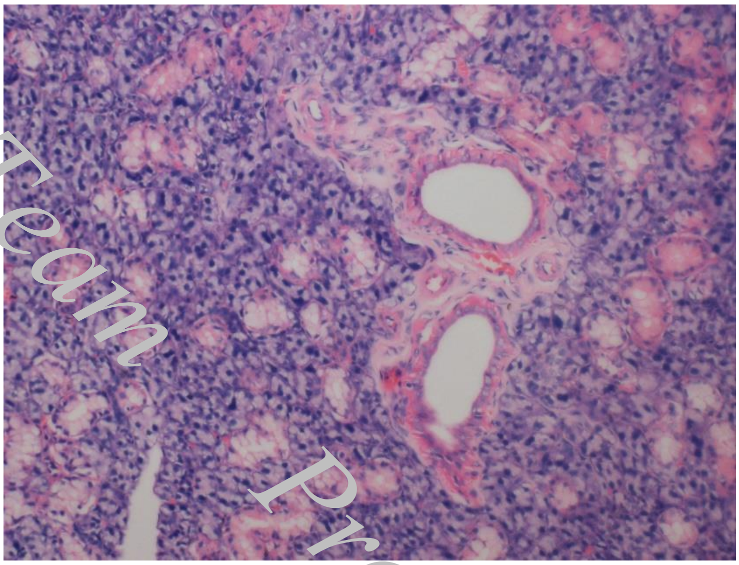

20×-D-Met

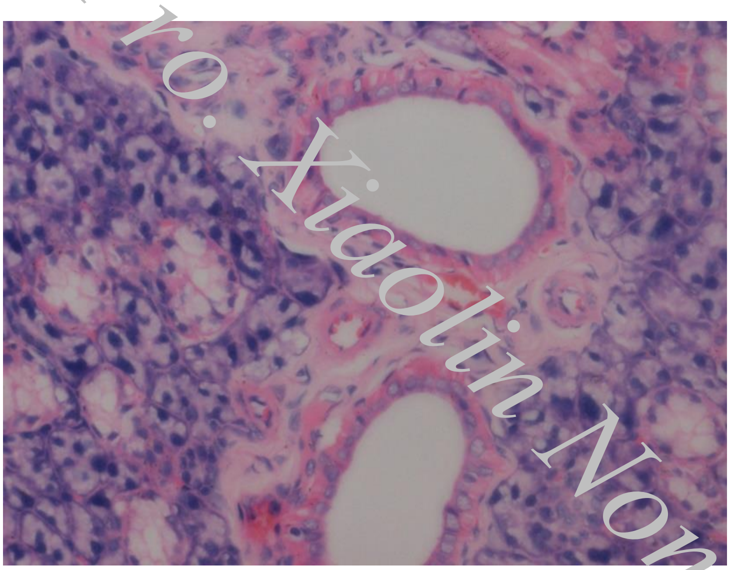

40×-D-Met

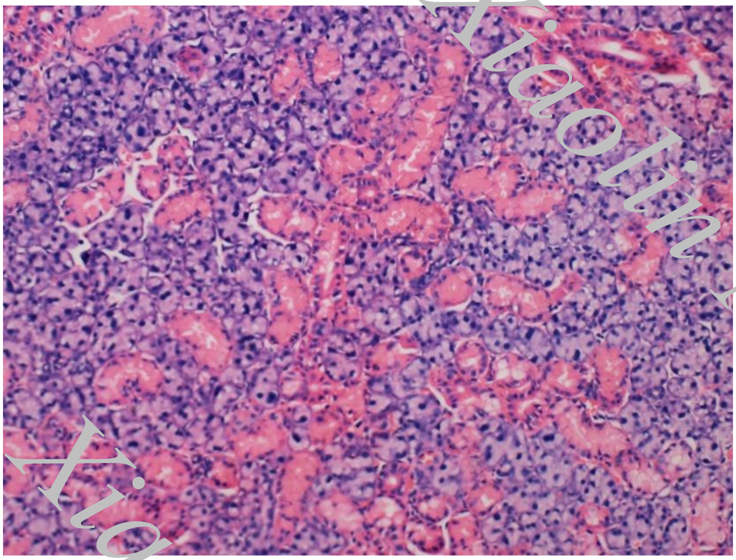

20×-D-Com

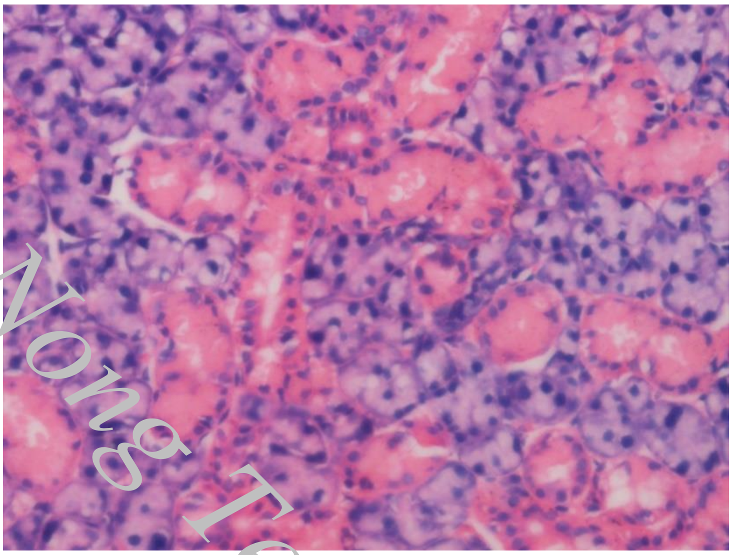

40×-D-Com

Pro. Xiaolin Nong Team

SSN（HE）

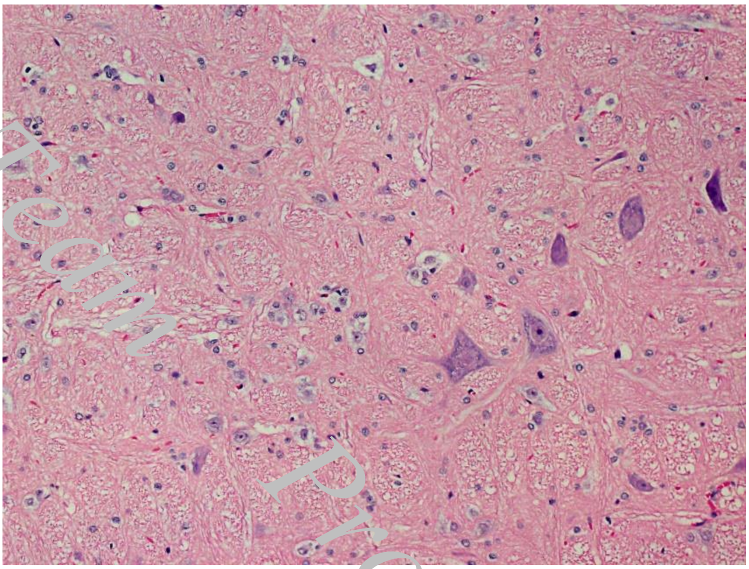

20×-Con

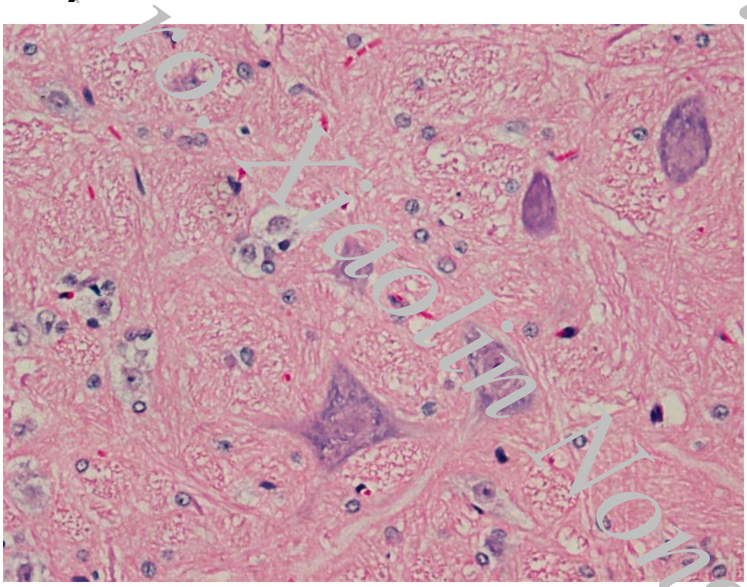

40×-Con

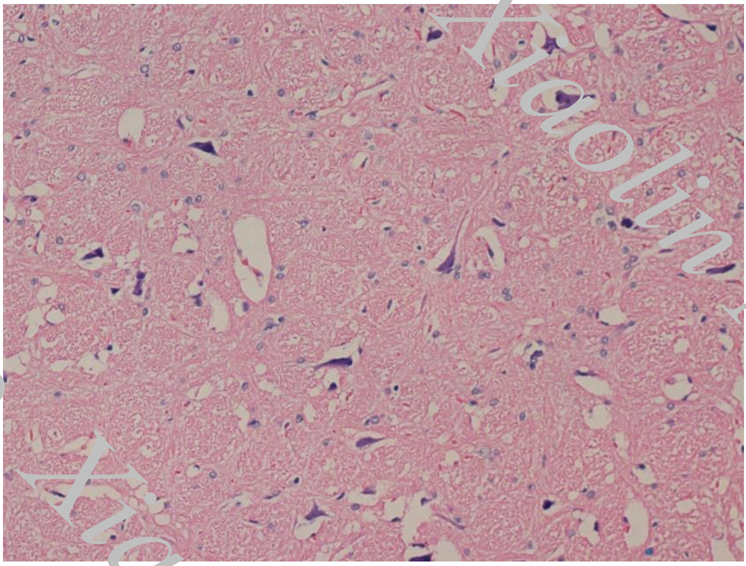

20×-Dia

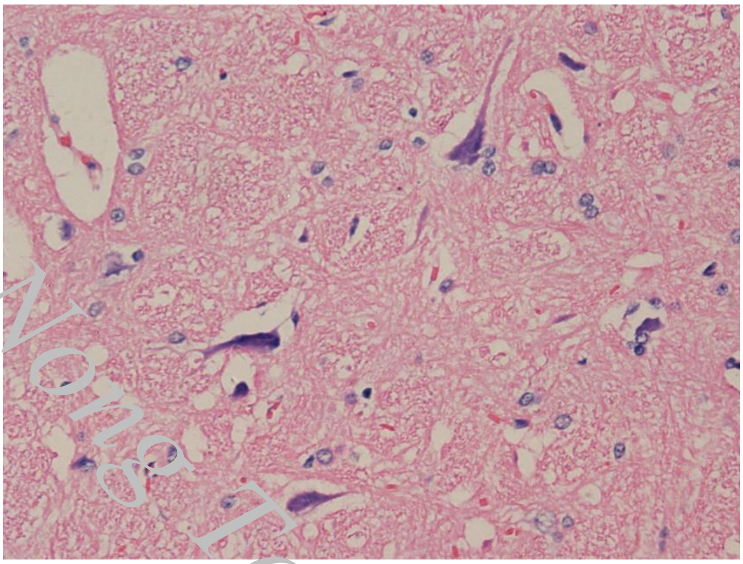

40×-Dia

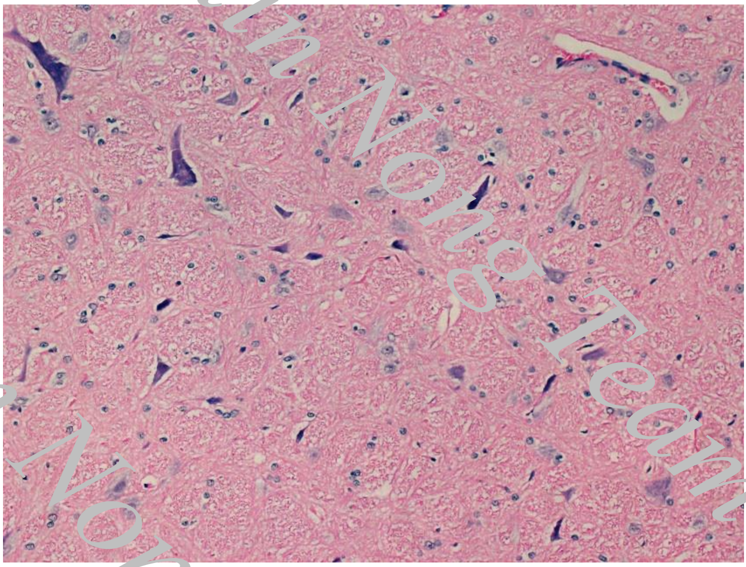

20×-D-Art

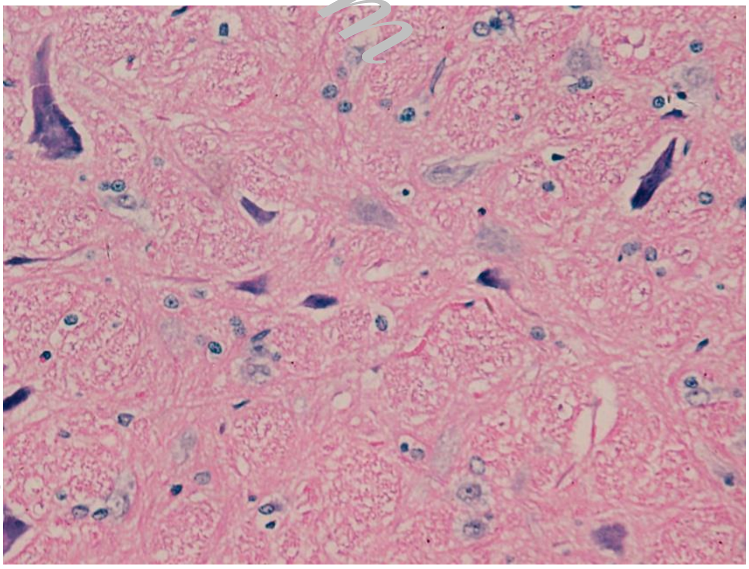

40×-D-Art

Pro. Xiaolin Nong Team

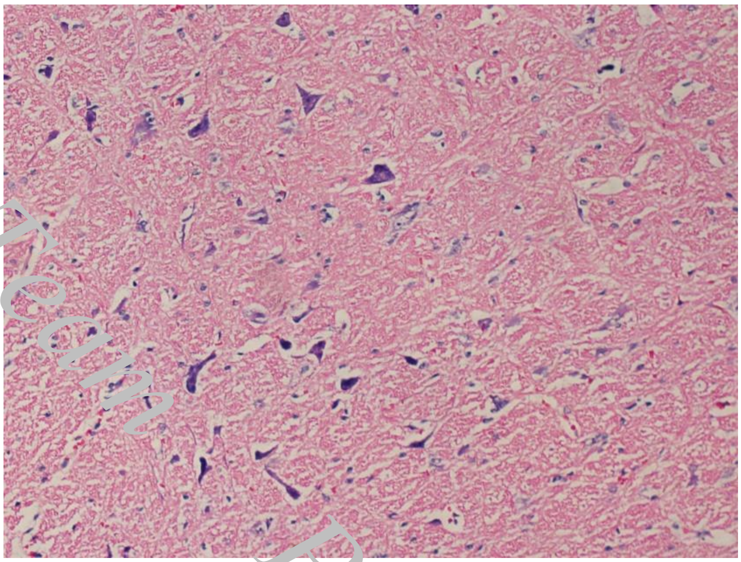

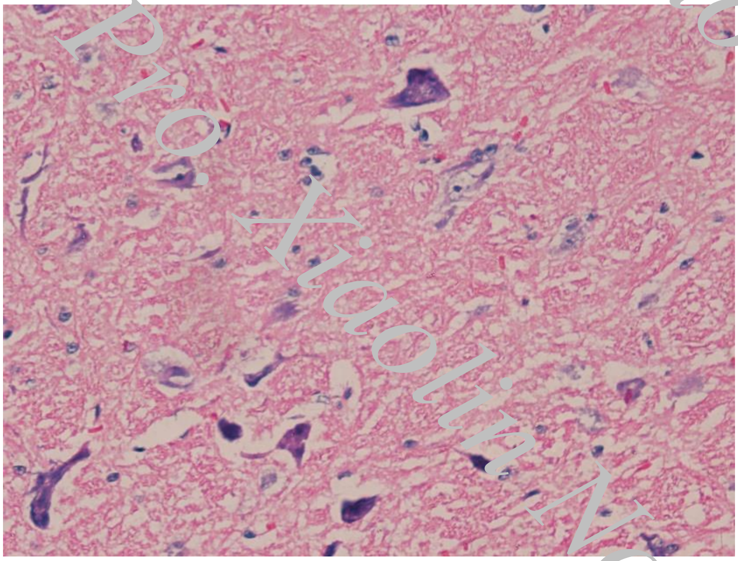

20×-D-Met

40×-D-Met

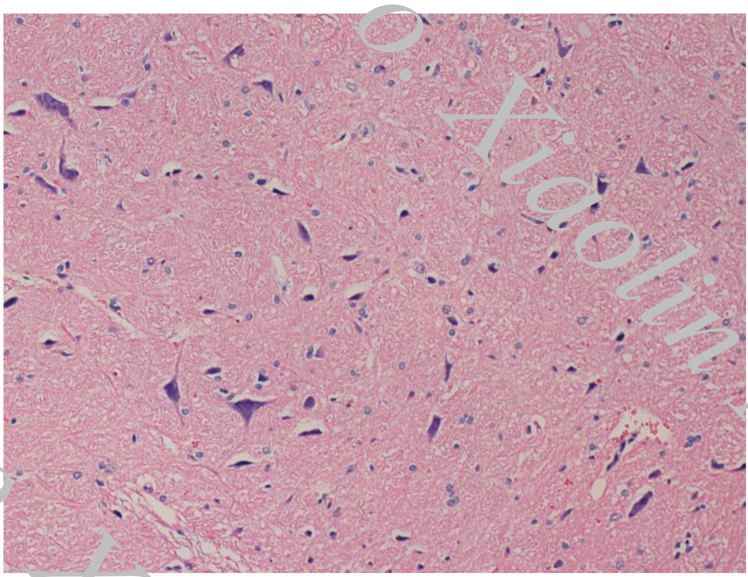

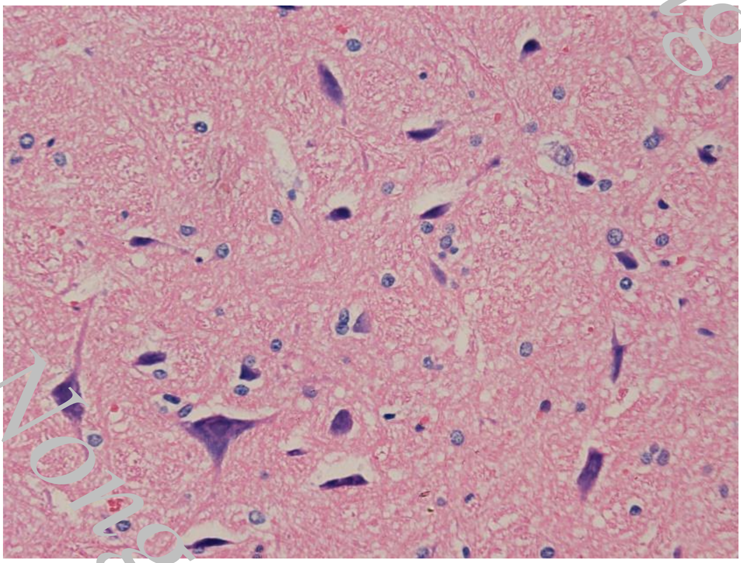

20×-D-Com

40×-D-Com

Pro. Xiaolin Nong Team

SSN（Nissl）

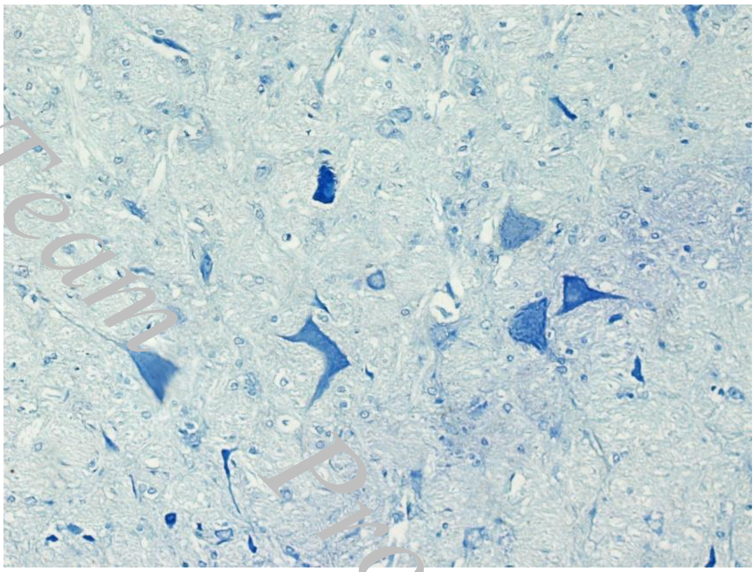
20×-Con

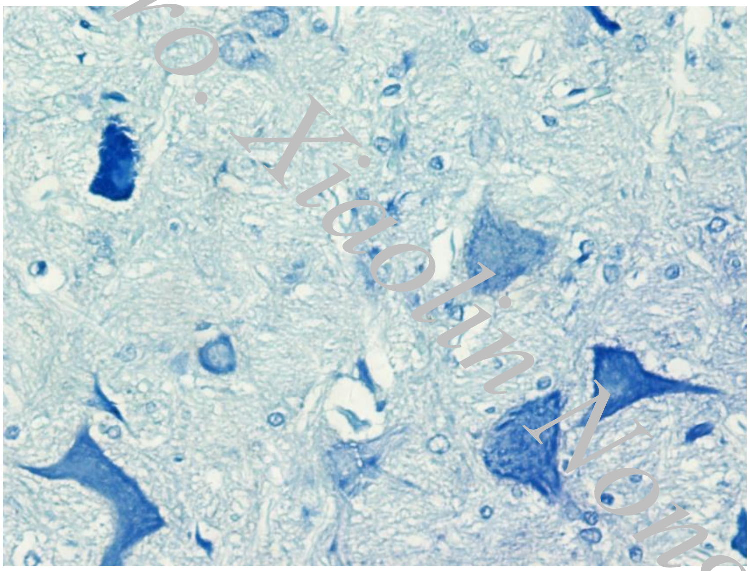
40×-Con

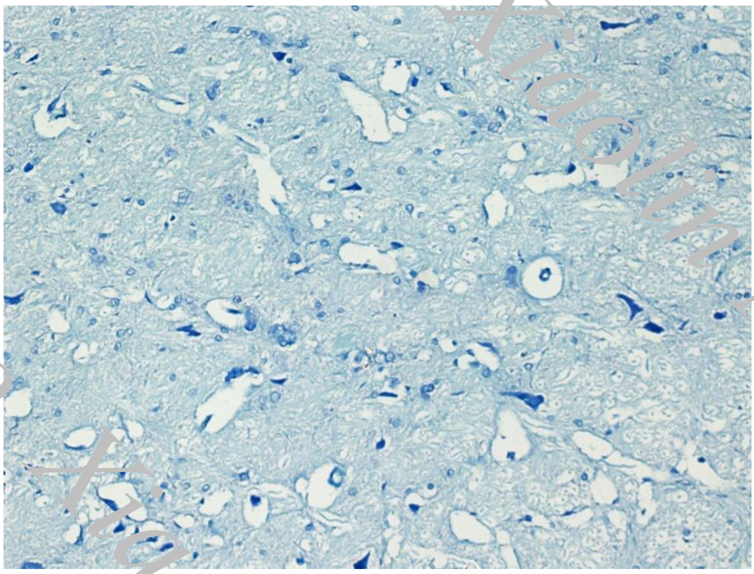
20×-Dia

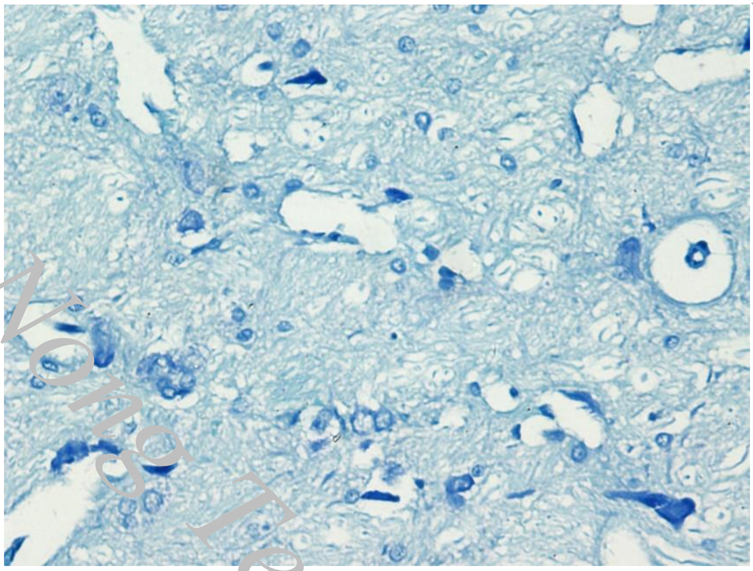
40×-Dia

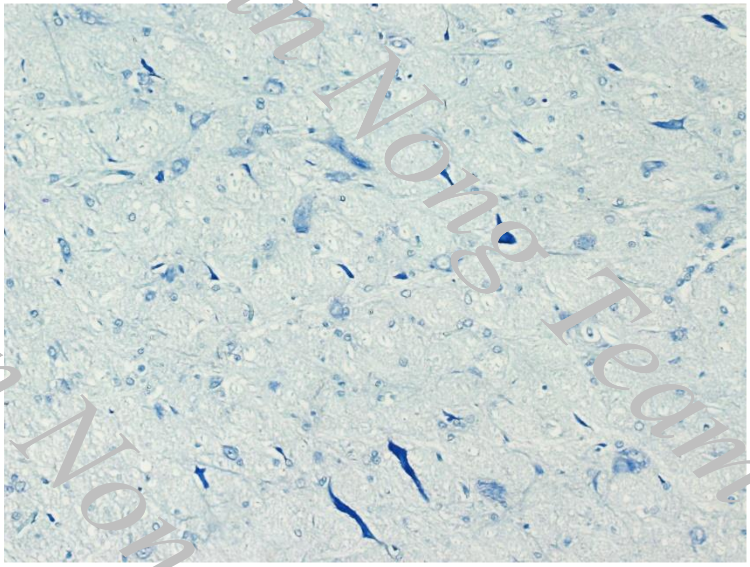
20×-D-Art

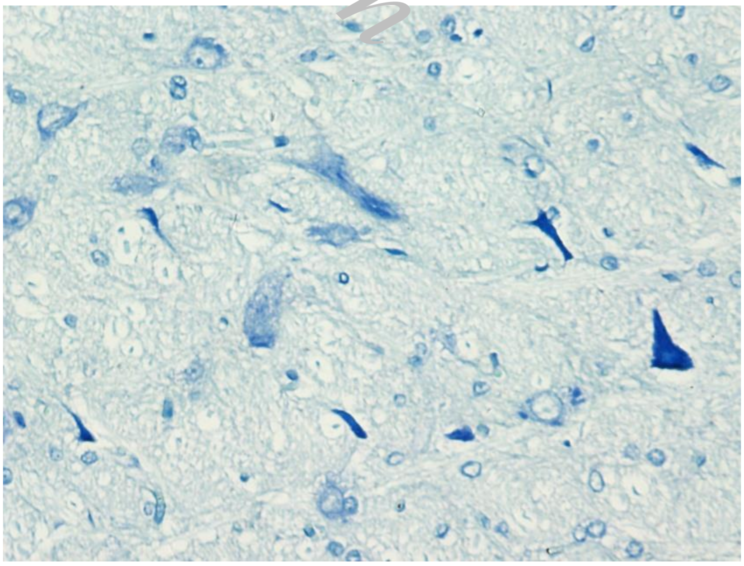
40×-D-Art

Pro. Xiaolin Nong Team

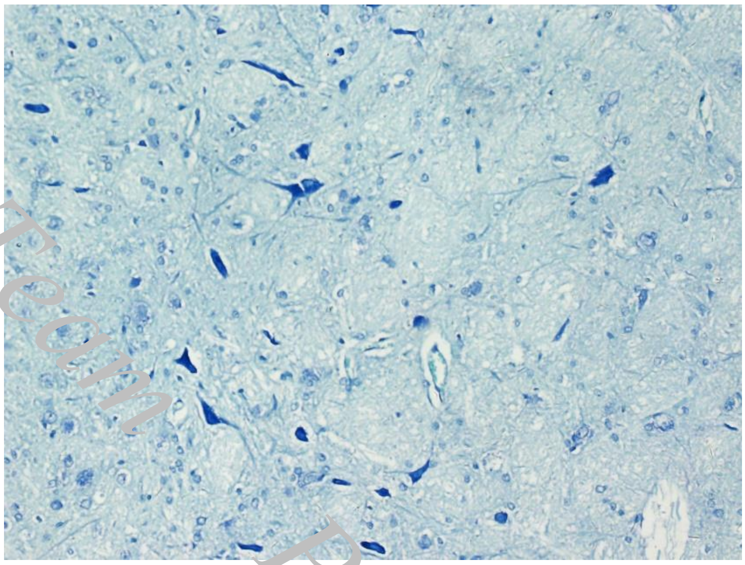

20×-D-Met

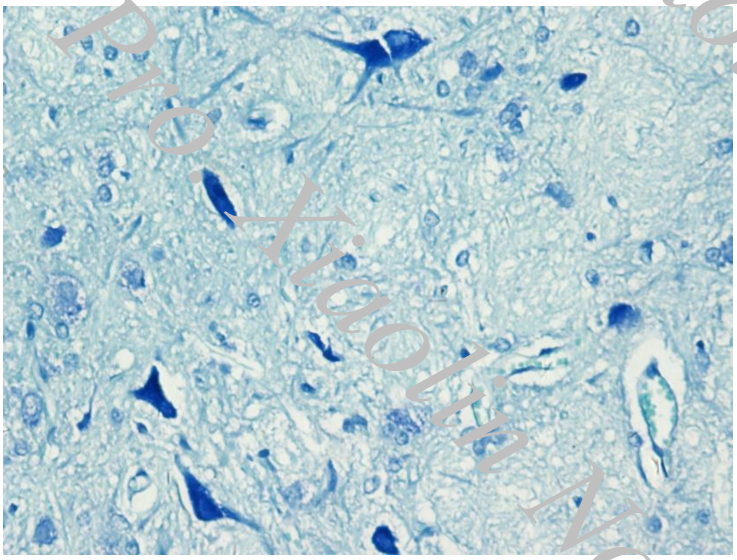

40×-D-Met

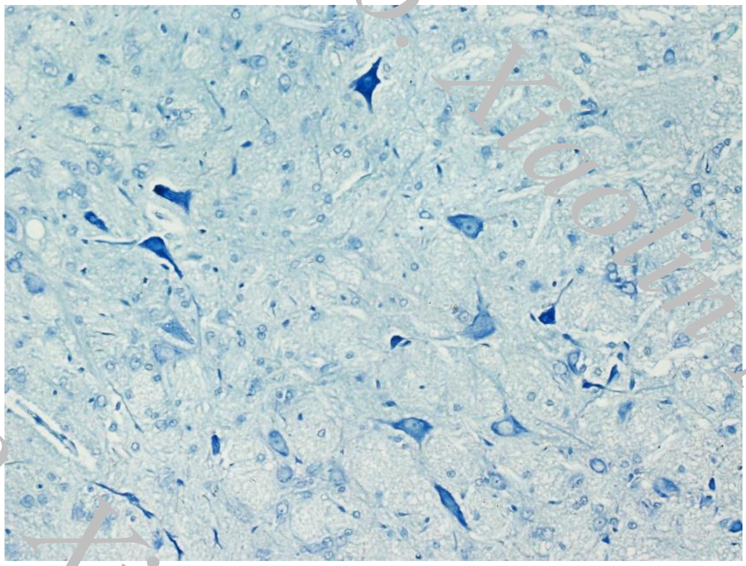

20×-D-Com

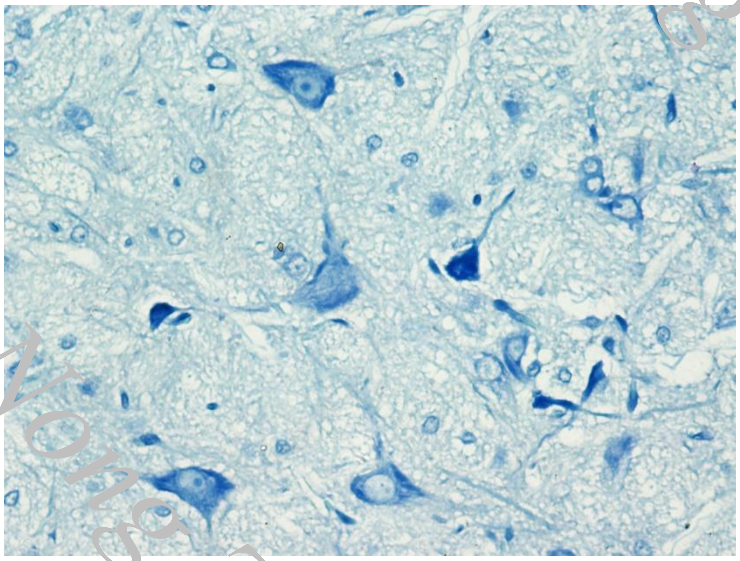

40×-D-Com

Pro. Xiaolin Nong Team
