# Supplementary material for: Artesunate Combined With Metformin Ameliorate on Diabetes-Induced Xerostomia by Mitigating Superior Salivatory Nucleus and Salivary Glands Injury in Type 2 Diabetic Rats via the PI3K/AKT Pathway
Source: Front Pharmacol. 2021 Dec 20;12:774674. doi: 10.3389/fphar.2021.774674 (PMC8722737; doi:10.3389/fphar.2021.774674)
Supplement: Supplementary file 4 [file DataSheet1.PDF]

Electron microscopy（SMG）

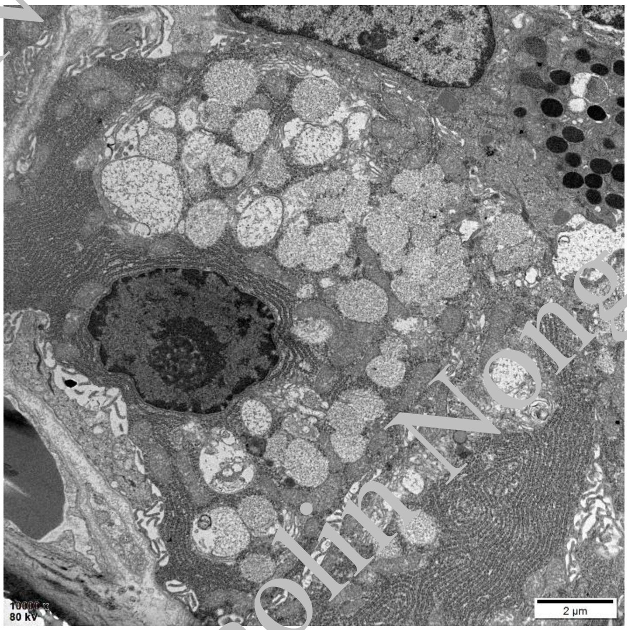

10000×-Con

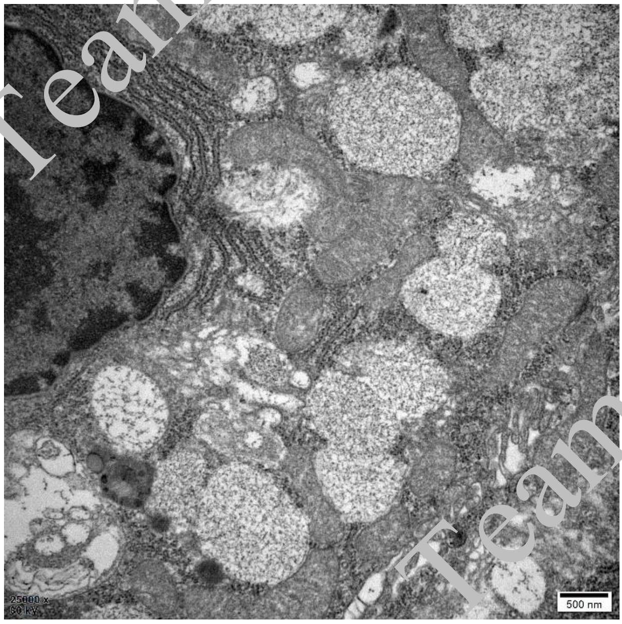

25000×-Con

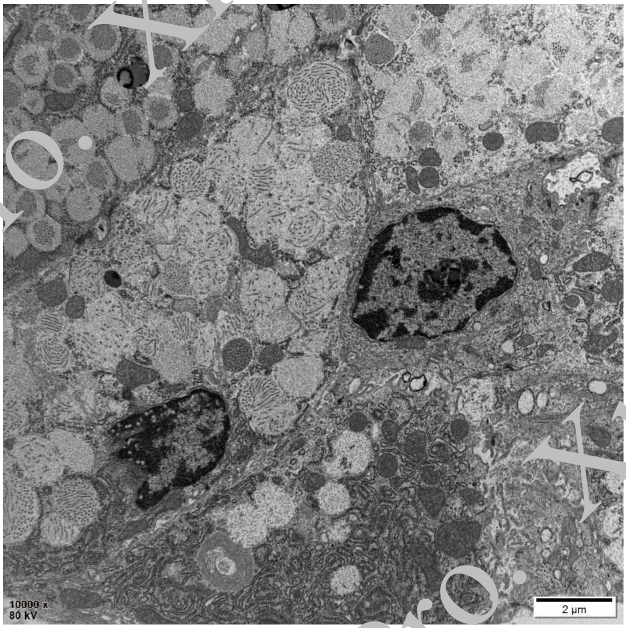

10000×-Dia

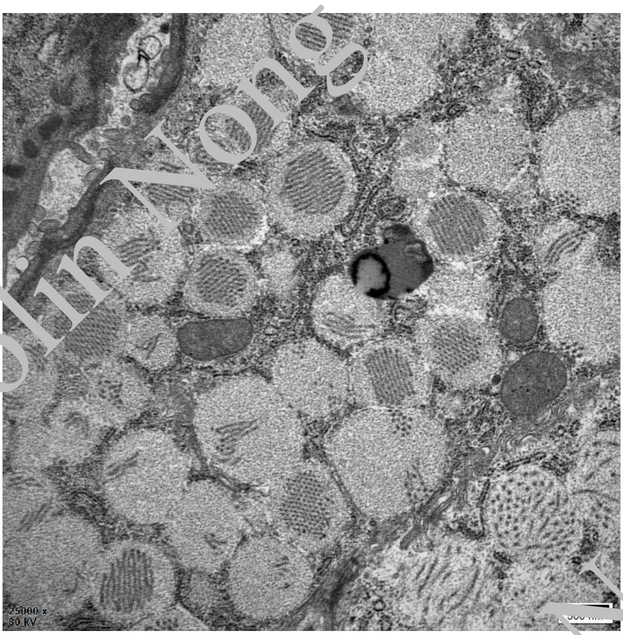

25000×-Dia

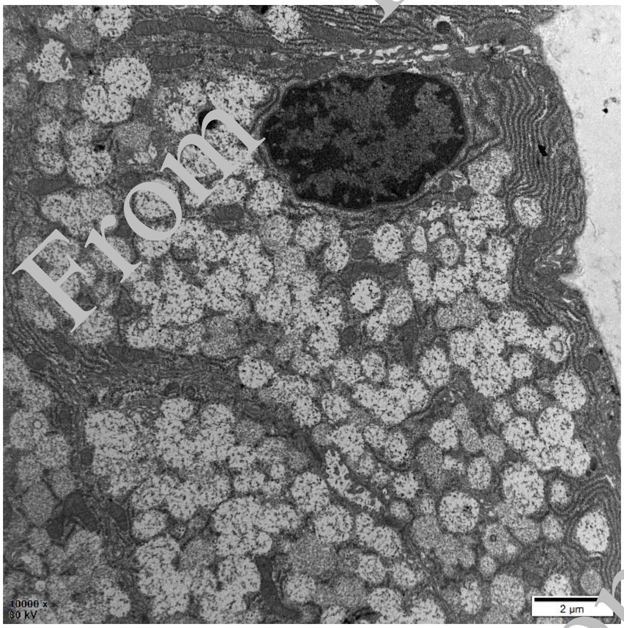

10000×-D-Art

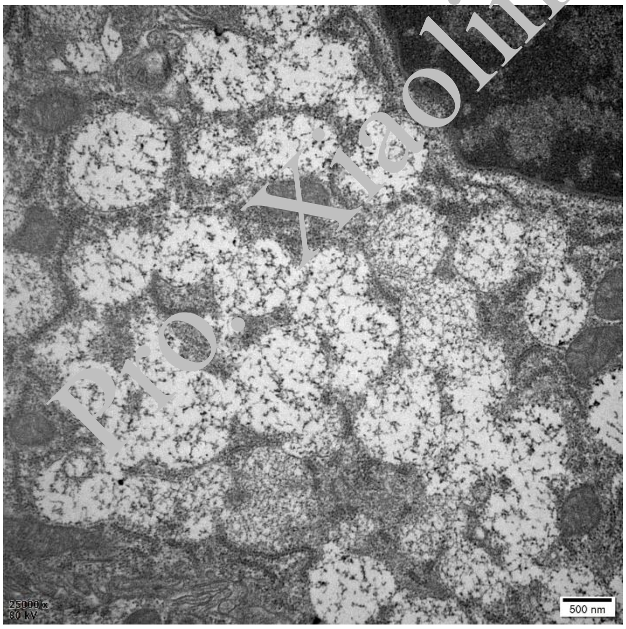

25000×-D-Art

From Pro. Xiaolin Nong Team

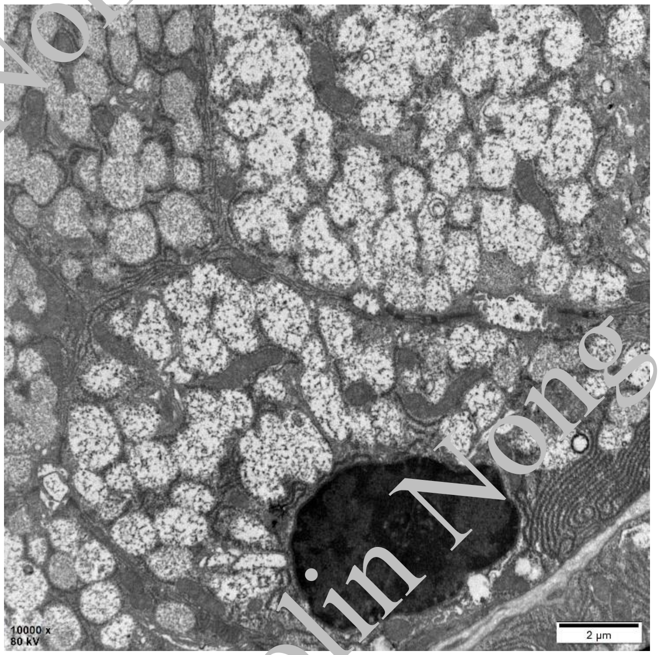

10000×-D-Met

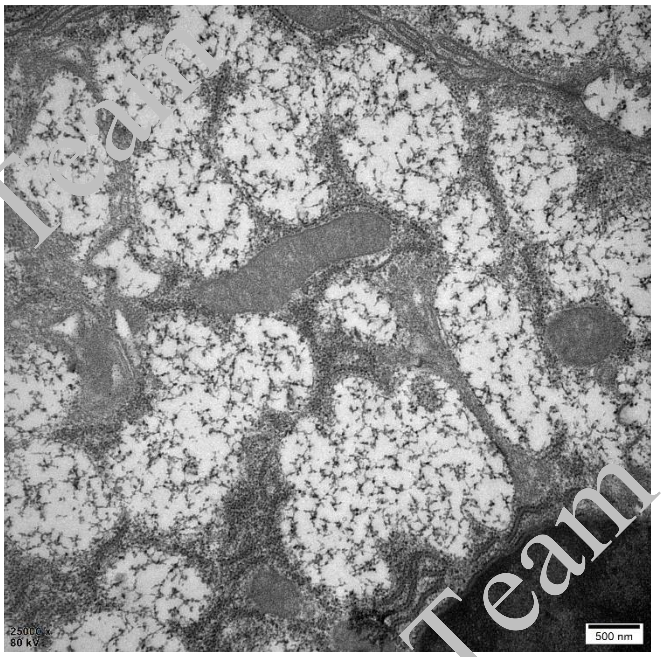

25000×-D-Met

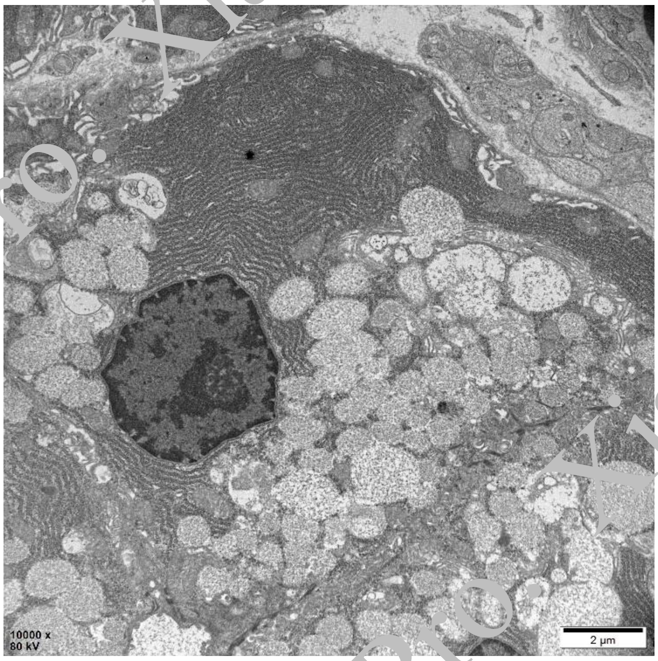

10000×-D-Com

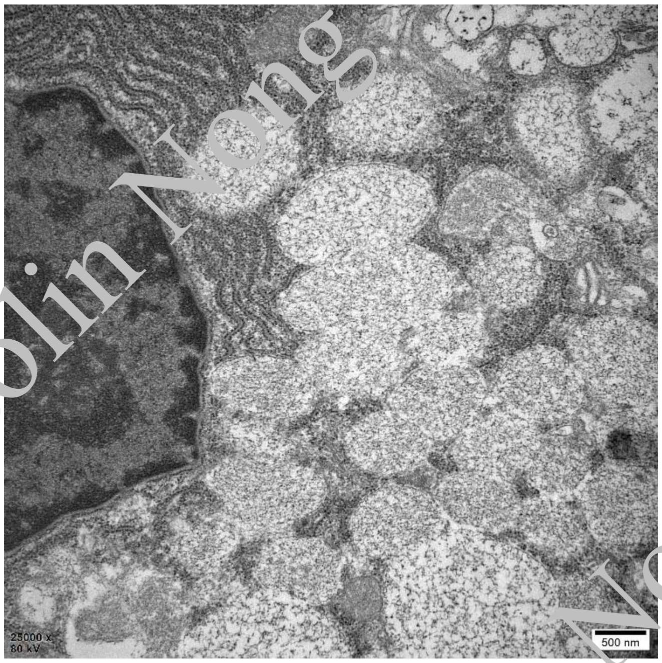

25000×-D-Com

From Pro. Xiaolin Nong Team

Electron microscopy（SSN）

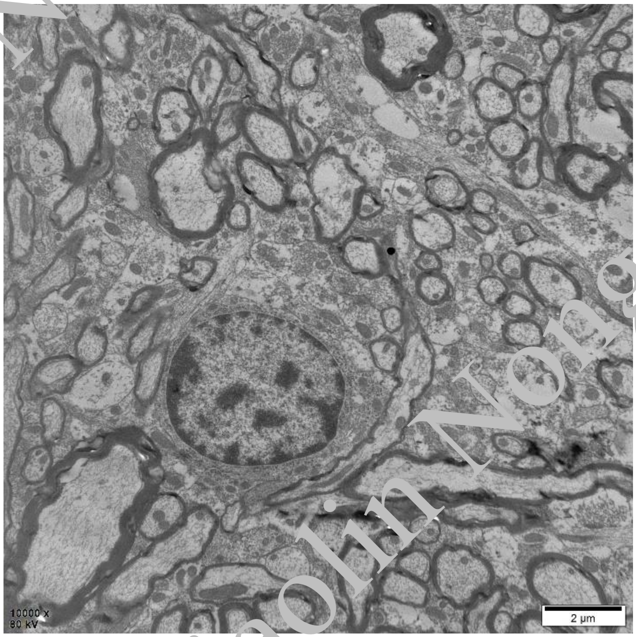

10000×-Con

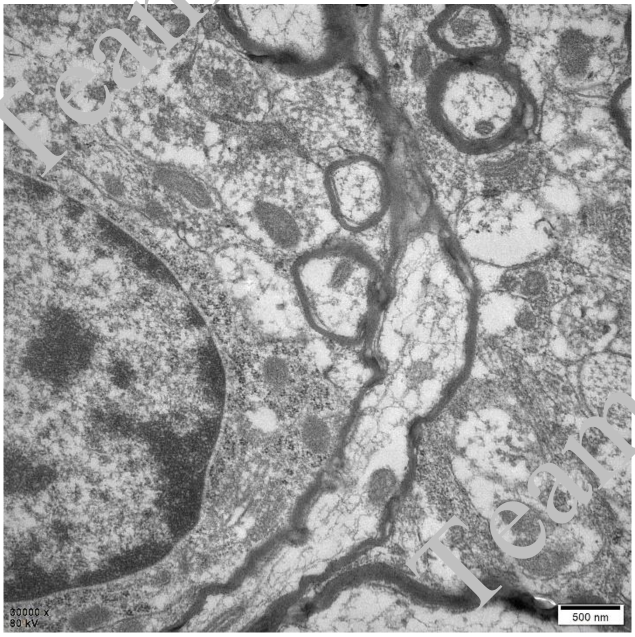

30000×-Con

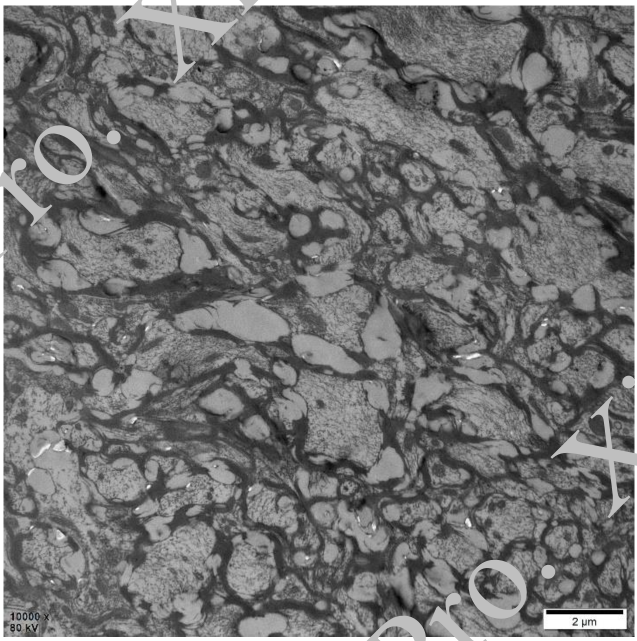

10000×-Dia

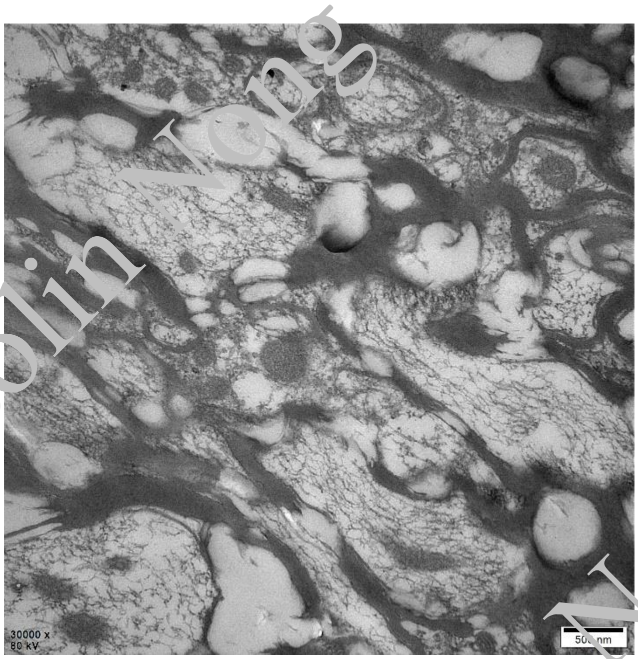

30000×-Dia

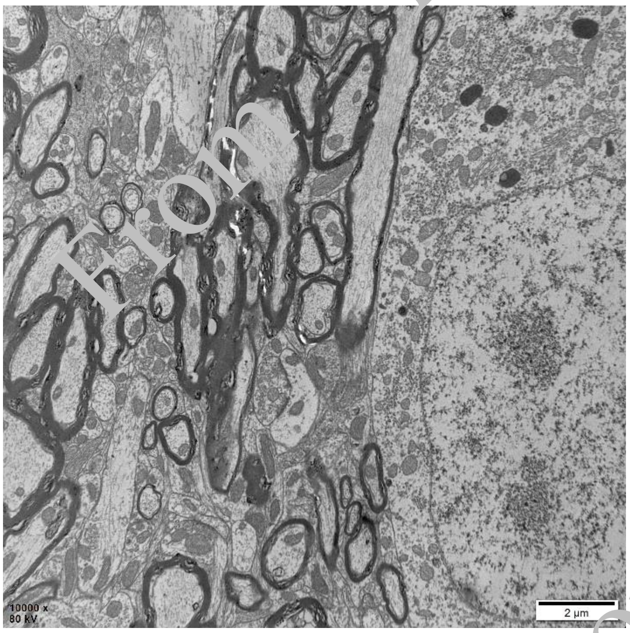

10000×-D-Art

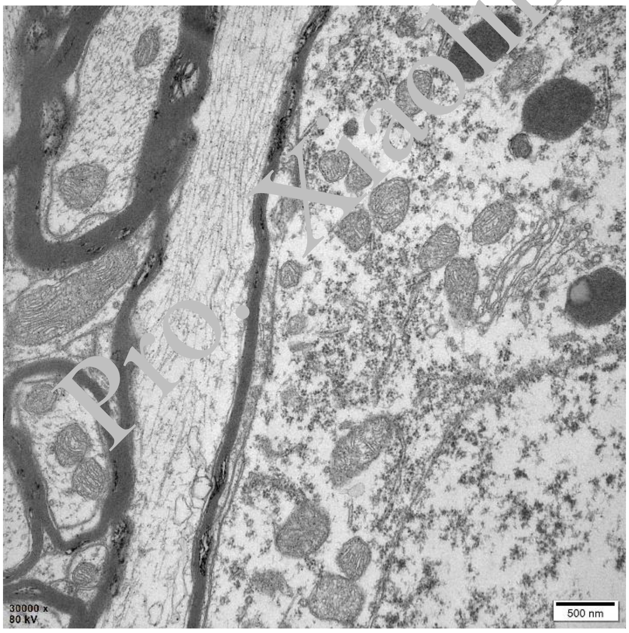

30000×-D-Art

From Pro. Xiaolin Nong Team

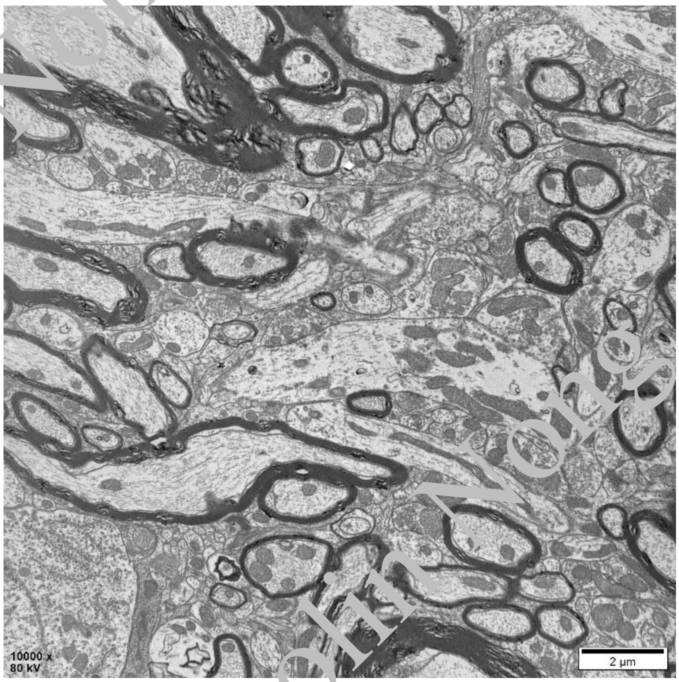

10000×-D-Met

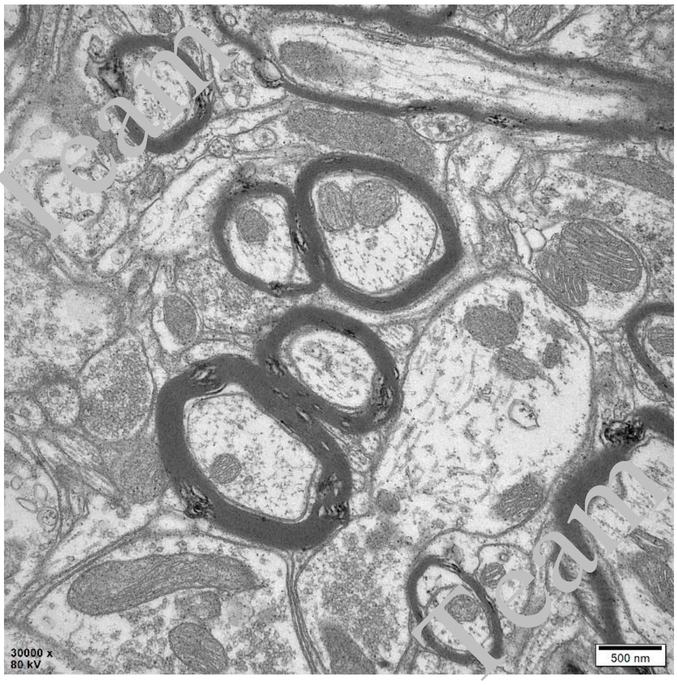

30000×-D-Met

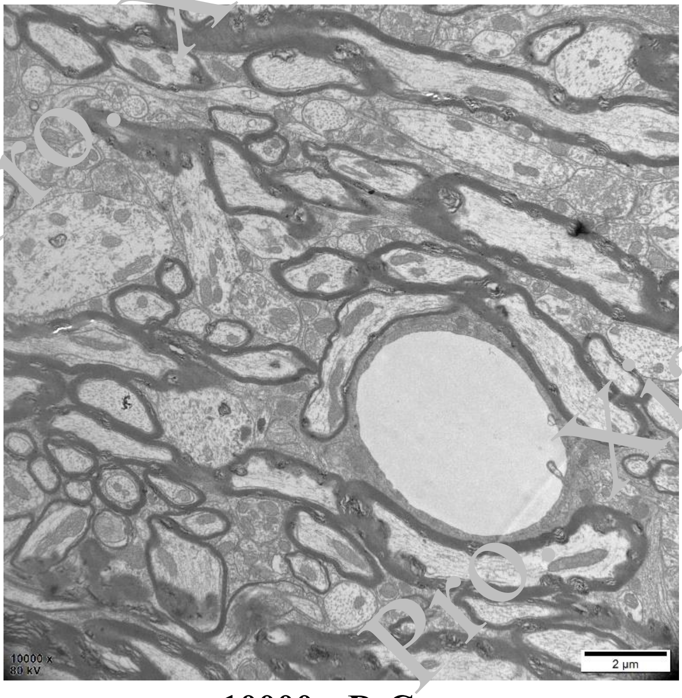

10000×-D-Com

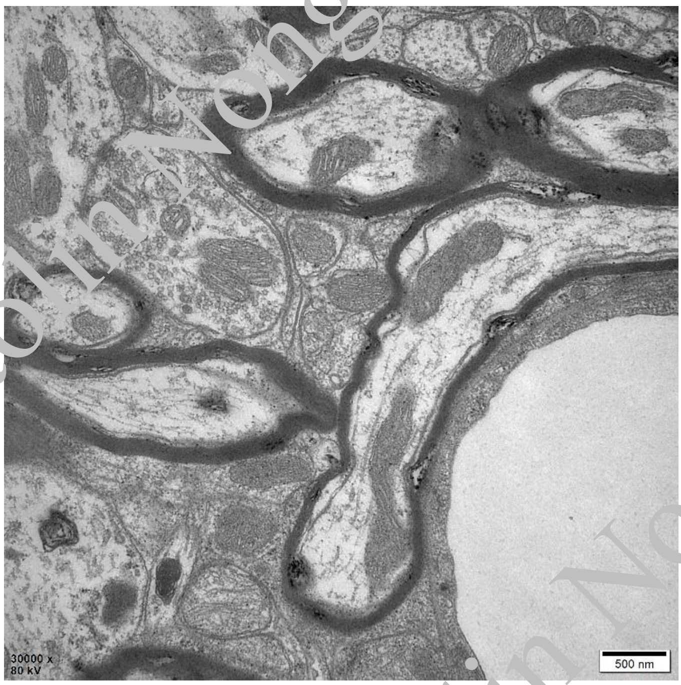

30000×-D-Com

From Pro. Xiaolin Nong Team
